# AQP5 complements LGR5 to determine the fates of gastric cancer stem cells through regulating ULK1 ubiquitination

**DOI:** 10.1186/s13046-022-02532-w

**Published:** 2022-11-14

**Authors:** Rou Zhao, Baoyu He, Qingli Bie, Jinghe Cao, Haoran Lu, Zhixin Zhang, Jing Liang, Li Wei, Huabao Xiong, Bin Zhang

**Affiliations:** 1 Department of Laboratory Medicine, Affiliated Hospital of Jining Medical University, Jining Medical University, Jining, Shandong People’s Republic of China; 2 Department of Hepatobiliary Surgery, Affiliated Hospital of Jining Medical University, Jining Medical University, Jining, Shandong People’s Republic of China; 3 Department of Gastrointestinal Surgery, Affiliated Hospital of Jining Medical University, Jining Medical University, Jining, Shandong People’s Republic of China; 4 Institute of Immunology and Molecular Medicine, Jining Medical University, Jining, Shandong People’s Republic of China; 5 Institute of Forensic Medicine and Laboratory Medicine, Jining Medical University, Jining, Shandong People’s Republic of China

**Keywords:** Cancer stem cells, AQP5, LGR5, Autophagy, ULK1, Gastric cancer

## Abstract

**Background:**

Cancer stem cells (CSCs) are regarded as the "seed cells" for tumorigenesis, metastasis, recurrence and drug resistance. However, specific surface markers of CSCs of different origins have not been documented.

**Methods:**

Single-cell sequencing was used to analyze the highly expressed genes in cancer stem cells of gastric cancer patients, and it was verified that AQP5 was specifically highly expressed in gastric cancer stem cells (GC-CSCs) in vivo and in vitro. The effect of AQP5-promoting LGR5 on the malignant biological function of GC-CSCs was investigated. The mechanism by which AQP5 affects GC-CSCs was explored through transcriptome sequencing, proteomic detection, mass spectrometry, etc.

**Results:**

We report the identification and validation of AQP5 as a potentially specific surface marker of GC-CSCs. AQP5 was significantly upregulated in CSCs isolated from gastric cancer patients and in spheroid cells, and AQP5 was coexpressed with the canonical stem marker LGR5. Biologically, AQP5 promoted the sphere formation, proliferation, migration and invasion of GC cells in vitro and enhanced tumorigenesis in vivo. Furthermore, AQP5 coordinated with LGR5 and synergistically promoted the tumorigenesis of GC-CSCs. At the mechanistic level, AQP5 activated autophagy by inducing the LC3I/LC3II transformation in GC-CSCs, which was crucial for the biological functions of AQP5. Finally, we demonstrated that AQP5 recruited the E3 ligase TRIM21 to the key autophagy protein ULK1 and induced the K63-mediated ubiquitination of ULK1.

**Conclusions:**

We elucidate a novel surface marker, AQP5, which is specifically expressed by GC-CSCs. Furthermore, our study creates a link between AQP5 and LGR5 and highlights the necessity of targeting both surface markers simultaneously as a promising approach for the treatment of gastric cancer patients.

**Supplementary Information:**

The online version contains supplementary material available at 10.1186/s13046-022-02532-w.

## Background

Gastric cancer (GC) is the third most common cause of cancer-related deaths worldwide [1]. Despite advances in diagnostic and therapeutic strategies, the clinical outcomes and prognosis of patients with GC remain unsatisfactory. Many studies have shown that dysregulated expression and accumulation of relevant genes are crucial for the development and occurrence of gastric cancer [2]. Thus, targeting these molecules has become crucial for tumor therapy in recent years. For example, cetuximab, the first monoclonal antibody that targets the epidermal growth factor receptor (EGFR) [3], is entering the clinical application stage, but the therapeutic effect has not reached the expected standard [4]. VEGF is another important target for GC treatment [5]. After treatment with monoclonal antibodies against VEGF, such as bevacizumab, patient prognosis did not reach statistical significance, and patients suffered strongly adverse reactions [6]. Therefore, elucidating novel functions of relevant genes that contribute to the pathogenesis of GC is crucial for the development of new effective therapies.

CSCs are a small subpopulation of quiescent cells with self-renewal abilities and pluripotency that can drive tumor initiation and cause relapses [7]. CSCs generate the bulk of tumors via their self-renewal and their ability to differentiate into multiple cellular subtypes [8]. Moreover, these CSCs acquire multidrug resistance, thus protecting themselves from most traditional chemotherapeutic agents. As a result, this small subpopulation of persistent cells forms more aggressive and chemoresistant tumors, resulting in the failure of cancer therapy [9]. Thus, identifying and targeting these rare cancer cells are believed to be important for understanding the etiology of cancer and developing a novel therapeutic strategy for cancer therapy [10].

The development of therapeutic strategies that target these tumor-initiating CSCs mainly relies on the use of cell surface markers to discriminate and identify CSCs [11]. For instance, leucine-rich repeat-containing G protein-coupled receptor 5 (LGR5), also known as G-protein coupled receptor 49 (GPR49), is a well-characterized stem cell surface marker that is expressed in several tissues/organs, including the stomach, small intestine, colon, and liver [12]. Accumulating evidence has demonstrated that LGR5 is a marker of resident adult epithelial stem cells at the gland base and that LGR5 + cells are multipotent stem cells that are responsible for the long-term renewal of the gastric epithelium or small intestine villus [13]. Moreover, LGR5 was recently reported to be highly upregulated in gastroenterological carcinoma [14]; the selective ablation of LGR5 + CSCs led to tumor regression, and targeting LGR5 + human colon CSCs enhanced the effects of chemotherapy [15]. Although it plays important regulatory effects in stem cells, LGR5 is highly expressed in both normal stem cells [16] and CSCs, which makes it difficult to distinguish between normal stem cells and CSCs in cancer-targeted therapy. Furthermore, LGR5 is also highly expressed in CSCs from tumors of various origins [17]. Thus, the identification of novel surface markers that can specifically identify and characterize CSCs from tumors of specific origins remains a challenge for understanding tumor biology and developing CSC-based therapeutic strategies.

Here, we examined single-cell transcriptome profiles of paired gastric mucosa tissues and gastric tumor tissues, and we identified and characterized a novel surface marker, namely, AQP5, that is more highly expressed in GC-CSCs than in gastric cancer epithelial cells and gastric mucosa stem cells (GM-SCs). The biological functions of AQP5 in gastric carcinogenesis were genetically assessed in several in vitro and in vivo models. Moreover, we demonstrated that AQP5 functions synergistically with LGR5 to determine the fates of GC-CSCs. Mechanistically, mass spectrometry combined with integrative analysis revealed that AQP5 enhances autophagy in GC-CSCs by interacting with the E3 ligase TRIM21 and promoting the ubiquitination of the key autophagy protein ULK1. Thus, our study identified a specific GC-CSC surface marker, AQP5, with biological, mechanistic, and clinical impacts on human gastric cancer. These findings highlight the importance of AQP5 in tumor biology, adding an important layer to the connection between AQP5 and gastric carcinogenesis, which can be translated into novel targeted therapies.

## Methods

### Patients

GCs and GMs were obtained from patients with gastric cancer who underwent surgery at the Affiliated Hospital of Jining Medical University. GC patients had primary, nonmetastatic gastric tumors and had not received radiotherapy or chemotherapy prior to surgery.

### Identification of cell types

Based on the single-cell reference expression quantitative public dataset, SingleR was used to calculate the correlation between the expression profiles of the cells to be identified and the reference dataset, and the cell type was determined according to the Spearman correlation coefficient.

### Screening of differentially expressed genes

The FindMarkers function in the Seurat package was used to identify differentially expressed genes, and significantly differentially expressed genes were identified based on the criteria of *p* value less than 0.05 and differential fold change greater than 2.5.

### Cell culture and transfection

The AGS (RRID: CVCL_0139) and HGC-27 (RRID: CVCL_1279) gastric cancer (GC) cell lines and the HEK 293 T (RRID: CVCL_D585) cell line were purchased from Procell. The cells were grown in DF-12 medium (Gibco) supplemented with 10% fetal bovine serum (Gibco). The cells were incubated at 37 °C in a humidified atmosphere with 5% CO_2_.

The cells were transfected with pSLenti-AQP5 and control vector according to the manufacturer's instructions. Stably transfected cell lines were obtained after selection with 1.5 μg/mL puroMycin (Gibco) for 6 days. The control and AQP5/LGR5-specific shRNA sequences are listed in Supplementary Table 4. Transfection of ULK1/TRIM21/Ubiquitin/k63R-Ubiquitin plasmid and control empty vector was performed using Lipofectamine 3000 reagent (Invitrogen) according to the manufacturer's instructions. Transfection of TRIM21/ATG7/UBB/UBC siRNA and negative control siRNA (NC) was performed using Lipofectamine 3000 reagent (Invitrogen) according to the manufacturer's instructions. The corresponding sequences are shown in Supplementary Table 4. The cells were harvested 48–72 h post-transfection for various assays.

## Results

### AQP5 is uniquely expressed in GC-CSCs and promotes gastric cancer development

**Fig. 1:**
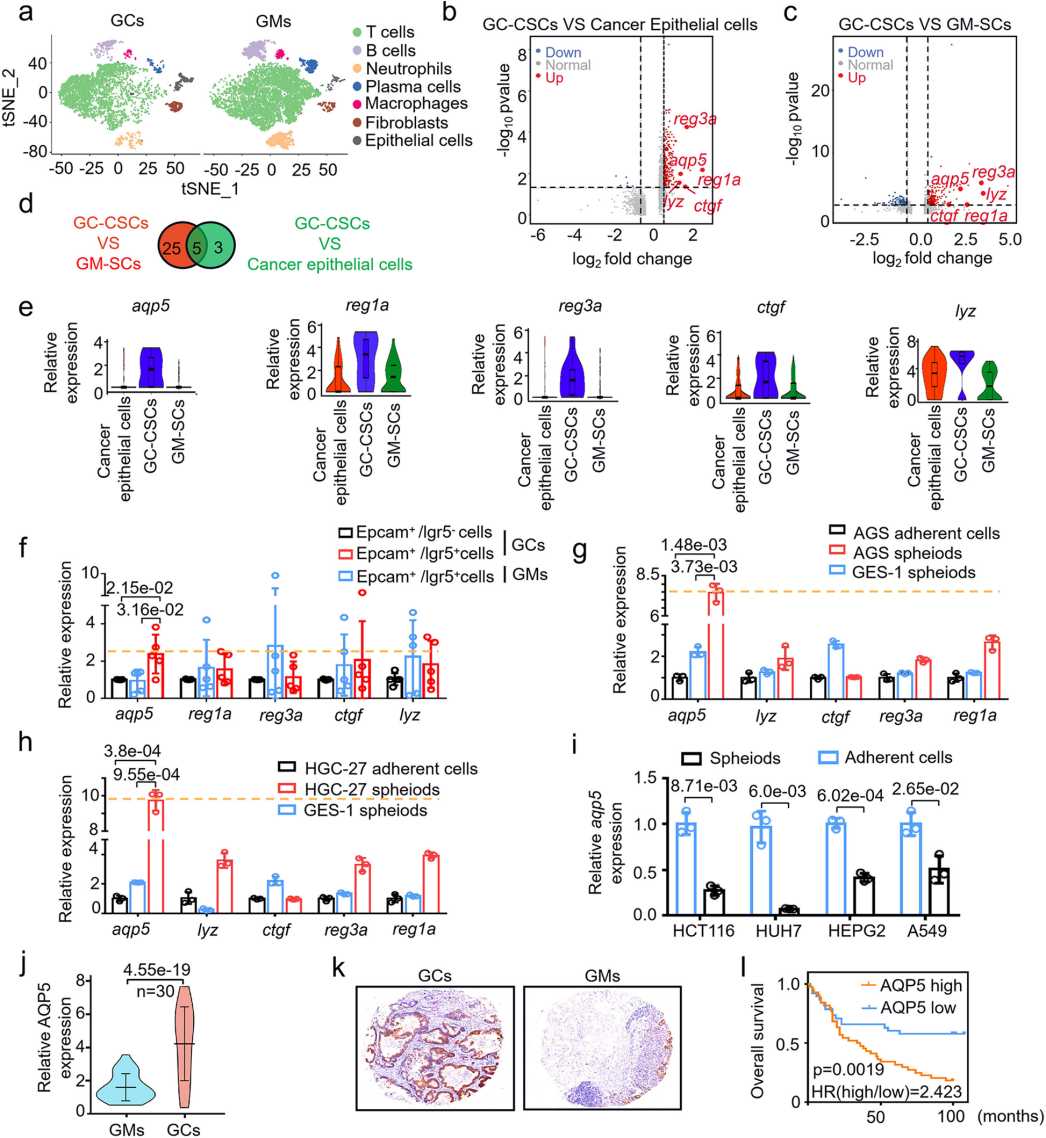
AQP5 is highly expressed in gastric cancer stem cells and has prognostic significance. **a** Cell atlas of GCs and GMs. **b** and **c** The differential gene signatures of GC-CSCs and gastric cancer epithelial cells or GM-SCs were plotted using a volcano program. **d** Overlapping candidate genes were obtained from the two differential gene signatures. **e** Violin plot of the expression of the five candidate genes. Epithelial cells were sorted with Anti-EPCAM Micobeads. Stem cells were sorted with Anti-EPCAM Micobeads and Anti-LGR5 Micobeads. **f** Expression levels of the five candidate genes were measured in cells sorted with the EPCAM and LGR5 markers. (g and h) Expression levels of the five candidate genes were measured in adherent cells and spheroids of AGS or HGC-27 (GC-CSCs) and in spheroids of GES-1 (GM-SCs). **i** HCT116, HUH7, HEPG2 and A549 cells were cultured as monolayers or under serum-free conditions as spheres. qRT‒PCR was used to assess the expression of aqp5.**j** Expression levels of aqp5 were measured in GCs and matched GMs (Cohort 1, *n* = 30, log-rank test, two-sided). **k** Immunohistochemical assessment of AQP5 expression in a microarray of GCs and matched GMs. **i** Survival was analyzed and compared between patients with high and low tumor expression of AQP5 in Cohort 2 (*n* = 82, log-rank test, two-sided). HR, hazard ratio

To explore whether there is any specific surface marker of CSCs from tumors of different origins, we performed single-cell transcriptome sequencing using gastric cancer tissues (GCs) and gastric mucosa tissues (GMs). According to canonical cell labeling, the cells were classified into seven major cell types (Fig. 1a). CD44 [18], CD24 and ALDH1A1 [19] was used to define stem cells. Among the GC-CSCs population, gastric cancer epithelial cells population and gastric mucosa stem cells (GM-SCs) population, there were 8 genes with higher expression in GC-CSCs than in gastric cancer epithelial cells (Fig. 1b) and 30 genes with higher expression in GC-CSCs than in GM-SCs (Fig. 1c) (Fc ≥ 2.5, *p* < 0.05). Five candidate genes were identified by overlapping the two gene sets, and these genes were aqp5, reg1a, reg3a, ctgf and lyz (Fig. 1d,e). LGR5 and CD133 are well-known CSCs markers [20]. Then, we identified and sorted epithelial cells (EpCAM +) [21] and stem cells (EpCAM + /CD133 + , EpCAM + /LGR5 +) from the GC and GM populations (Figure S1a, b). As shown in Fig. 1f and Figure S2c, aqp5 was ranked as the highest upregulated gene in EpCAM + /LGR5 + GC cells vesus other 4 genes, although the upregulation was not obvious in CD133 + GC cells. This finding was further confirmed in spheroids generated from AGS, HGC-27 (GC-CSCs) and GES-1 (GM-SCs) (Figure S1c-e). Consistently, among the 5 candidate genes, aqp5 expression was highest in spheroid cells (Fig. 1g, h and Figure S2a). Furthermore, we analyzed the expression of AQP5 in CSCs from tumors of other organs, including the lung, liver, intestine, etc. Interestingly, we found that AQP5 expression was restricted in GC-CSCs (Figure S2b, Fig. 1i). Thus, the results suggest that AQP5 could potentially be a specific biomarker of GC-CSCs.

### AQP5 plays an oncogenic role in gastric cancer development

Next, we examined the impact of AQP5 on gastric cancer development and analyzed AQP5 mRNA and protein levels in a cohort of 30 GCs and GMs. The mRNA and protein levels of AQP5 were significantly upregulated in the GCs compared to the GMs (Fig. 1j and Figure S3a). Then, we evaluated the pathological and clinical value of AQP5 using a gastric cancer tissue microarray. As shown in Fig. 1k and l, high levels of AQP5 expression were significantly associated with poor survival of GC patients (HR = 2.423, 95% CI 1.406 ~ 4.174, *p* = 0.0019). Moreover, multivariate regression analysis demonstrated that AQP5 expression was positively correlated with tumor grade (*p* = 0.012) in GCs (Table 1). Thus, AQP5 is highly expressed and clinically correlated with the progression of gastric cancer.

**Table 1:** Correlation between AQP5 expression and clinicopathological characteristics

Clinico-pathological features		cases	AQP5 expression		χ^2^ value	*P* value
			low	high		
Gender	female	31	14	17	0.028	0.867
	male	51	24	27		
Age	≤ 60	28	13	15	0.001	0.981
	> 60	52	24	28		
	unknown	2				
Grade	moderate differentiation	14	11	3	8.883	0.012
	poor differentiation	58	25	33		
	undifferentiation	10	2	8		
Pathological type	Borromann I	4	1	3	2.178	0.536
	Borromann II	29	14	15		
	Borromann III	30	11	19		
	Borromann IV	5	1	4		
	others	14				
T stage	T1-2	16	10	6	1.945	0.163
	T3-4	65	28	37		
	unknown	1				
N stage	N0	20	11	9	0.697	0.404
	N1-3	61	27	34		
	unknown	1				
M stage	M0	73	35	38	0.688	0.407
	M1	9	3	6		
TNM stage	1–2	32	19	13	3.298	0.069
	3–4	49	19	30		
	unknown	1				

In addition, we wanted to analyze the biological functions of AQP5 in gastric cancer development. As shown in Figure S3b–i, knockdown of AQP5 reduced cell proliferation, colony formation, and cell migration. Conversely, overexpression of AQP5 substantially enhanced cell proliferation, migration and clone formation. The oncogenic role of AQP5 was confirmed in xenograft models. The results showed that AQP5 knockdown significantly suppressed tumor growth, while AQP5 overexpression promoted tumor growth, as indicated by the xenograft tumor growth curve and tumor weight (Figure S3j and S3k). In addition, subcutaneous tumors overexpressing AQP5 exhibited higher expression of the markers Ki67, CD133 and LGR5. On the contrary, the protein expression pattern of the differentiation marker CK18 showed the opposite trend (Figure S3i, m). Together, these findings suggest that AQP5 plays a critical role in the tumorigenesis of gastric cancer.

### Biological relationship of AQP5 with classic GC-CSC markers

**Fig. 2:**
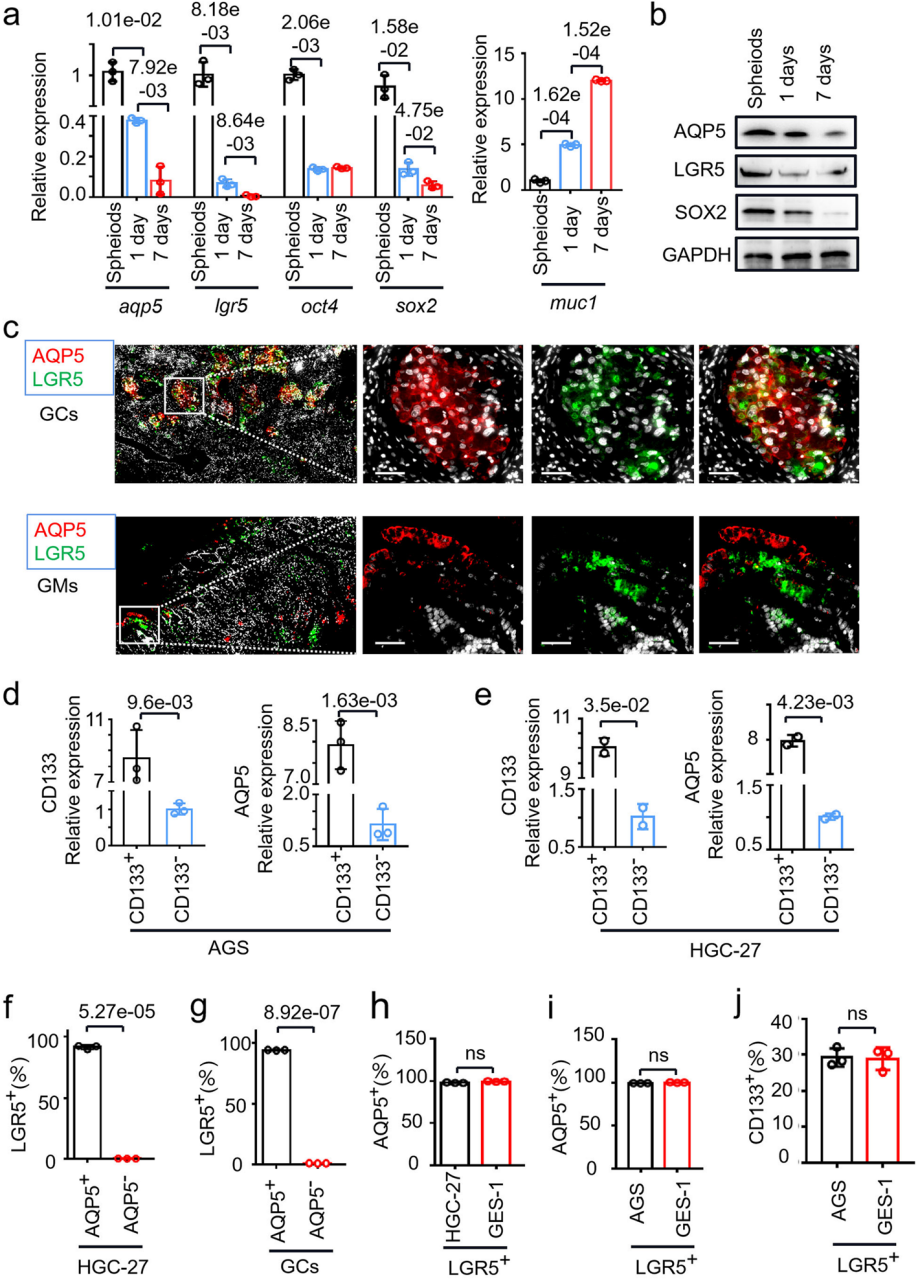
AQP5 expression correlation with stemness marker expression. **a** The mRNA expression levels of aqp5, lgr5, oct4, sox2 and muc1 were evaluated in spheroids and re-adherent cultured AGS cells. **b** The protein levels of AQP5, LGR5 and SOX2 were measured in spheroid and re-adherent cultured cells. **c** Multicolor immunofluorescence was used to assess the localization of LGR5 (green) and AQP5 (red) in GCs and GMs. **d** and **e** Expression level of AQP5 in CD133-positive (CD133^+^) and CD133-negative (CD133^−^) cells sorted from AGS (**d**) and HGC-27 (**e**) cells. (**f** and **g**) Statistical analysis of the proportion of LGR5^+^ cells in the AQP5^+^ or AQP5^−^ subgroup sorted from the HGC-27 cell line (**f**) and GC cells (**g**). **h** and **i** Statistical analysis of the proportions of AQP5^+^ cells in the LGR5^+^ subgroup sorted from HGC-27 (h), AGS (**i**) and GES-1 cells. (**j**) Statistical analysis of the proportion of CD133^+^ cells in the LGR5^+^ subgroups of AGS and GES-1 cells

To delineate the mutual interaction of AQP5 with classical stem cell markers, we examined gastric cancer data in TCGA. Correlation analysis showed that AQP5 expression was significantly correlated with the expression of CD133, OCT4, ALDH1A1 and CD24 (Figure S4a). Then, we cultured spheroid monolayers for 1 to 7 days to re-differentiate into GC-CSCs as shown in Figure S4b. The results demonstrated that with the GC-CSCs differentiation time extended, the expression level of AQP5 gradually decreased, which was consistent with the trends of changes in the expression of the stemness markers LGR5 and SOX2 (Fig. 2a, b). These data suggest that AQP5 is closely associated with these stem cell markers.

To confirm the expression pattern of AQP5 in gastric cancer, we performed multicolor immunofluorescence. As shown in Fig. 2c, AQP5 and LGR5 were colocalized in gastric cancer tissue cells. More importantly, AQP5 and LGR5 showed almost no colocalization in gastric mucosa tissue cells, further confirming that AQP5 was specifically expressed in GC-CSCs. Next, we sorted CD133-positive (CD133^+^) and CD133-negative (CD133^−^) cells and verified that the expression of AQP5 was significantly increased in CD133^+^ cells (Fig. 2d, e). These data suggest that AQP5 and LGR5/CD133 are co-expressed in gastric cancer.

Furthermore, we measured the expression of AQP5 and LGR5 in GC-CSCs that were sorted and purified from GC tissue. As shown in Fig. 2f, g and Figure S4c, d, almost all the AQP5^+^ cells harbored high LGR5 expression, while AQP5^−^ cells harbored low LGR5 expression, reconfirming that AQP5 and LGR5 were co-expressed in the same type of cancer cells. Interestingly, the expression level of AQP5 in LGR5 + GC cells (HGC-27, AGS) was as same as gastric mucosal cells (GES-1) (Fig. 2h, i, Figure S4g, h). Moreover, the expression level of AQP5 in LGR5 + cells of GCs was also the same as that of GMs (Figure S4e, f). Futhermore, there was no difference in the expression of CD133 between these two cell types (Fig. 2j, Figure S4i). Collectively, the data suggest that AQP5 is a specific biomarker for GC-CSCs.

### AQP5 promotes the self-renewal and tumorigenesis of GC-CSCs

**Fig. 3:**
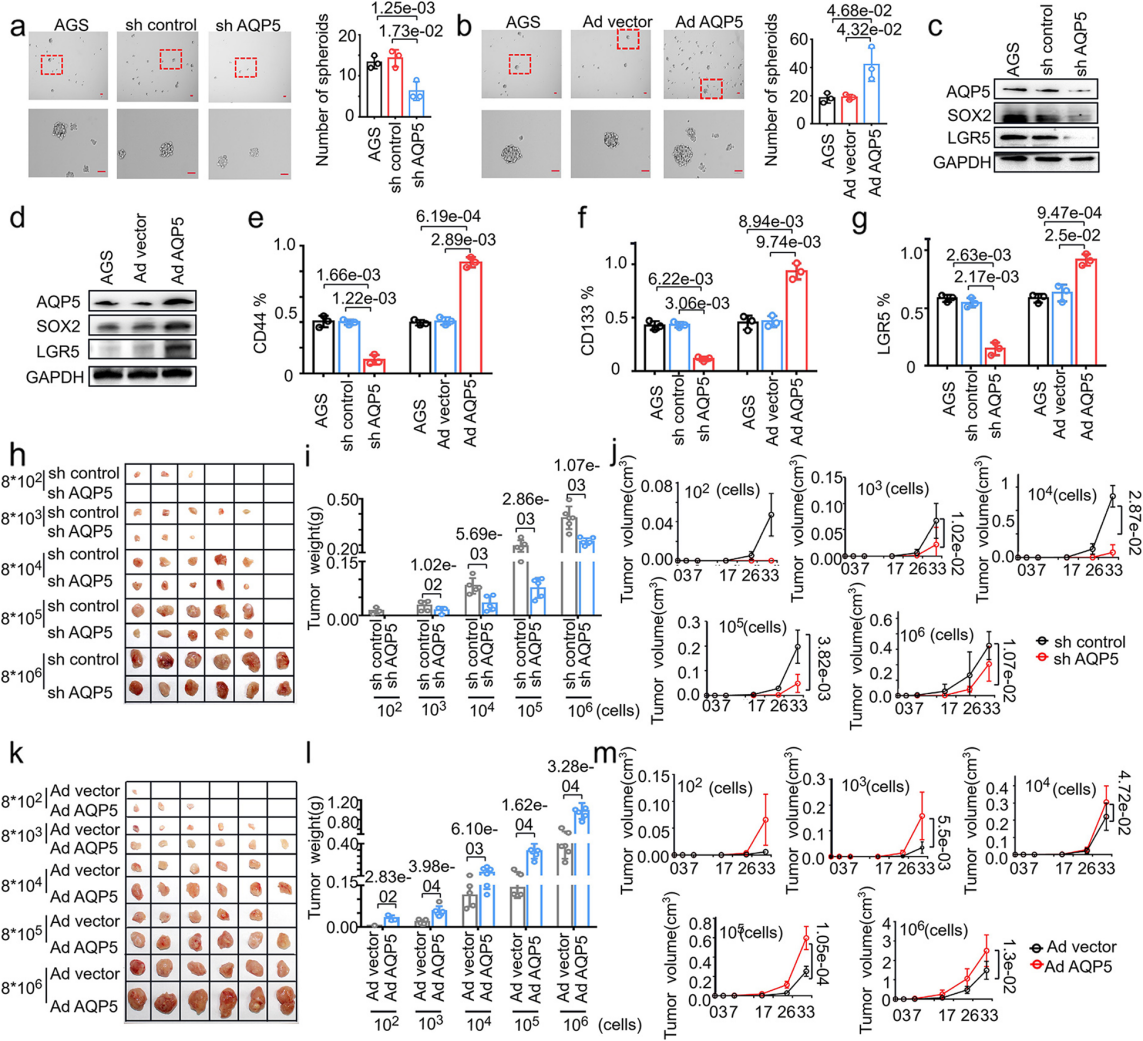
AQP5 promotes the malignant biological function of GC-CSCs in vitro and in vivo. **a** and **b** Representative images of exogenous AQP5 knockdown (**a**) or AQP5 overexpressing (**b**) AGS cells cultured in serum-free medium for 10 days. Statistical analysis was performed on the number of spheroids (diameter > 50 μm). (**c** and **d**) The expression levels of AQP5, LGR5 and SOX2 were measured using WB analyses after AQP5 overexpression and knockdown. **e**–**g** Flow cytometric analysis of CD44, CD133 and LGR5 expression in AQP5-overexpressing or AQP5-knockdown HGC-27 cells. **h**-**m** AQP5 was knocked down (**h**) or overexpressed (**k**) in HGC-27 cells. These cells were diluted and subcutaneously injected into severely immunodeficient mice. Tumors were examined over a 33-day period (*n* = 6 for each group). The tumor weight (**i**, **l**) and tumor volume (**j**, **m**) were monitored in the indicated groups and at the indicated time points

We next investigated the impacts of AQP5 on the biological functions of GC-CSCs. As expected, sphere formation was dramatically reduced upon AQP5 knockdown. Conversely, overexpression of AQP5 enhanced sphere formation (Fig. 3a, b and Figure S5a-c). To further verify the effect of AQP5 on the stemness of GC-CSCs, we measured the expression of stem cell markers. As shown in Fig. 3c, d and Figure S5d, e, knockdown of AQP5 markedly inhibited the expression of the stem cell markers LGR5 and SOX2, while overexpression of AQP5 increased the expression of LGR5 and SOX2, more importantly, knockdown of LGR5 also significantly decreased the expression of AQP5. We further verified the effect of AQP5 on the expression of stem cell markers. As shown in Fig. 3e-g and Figure S5f-h, knockdown of AQP5 decreased the proportion of CD44^+^/CD133^+^/LGR5^+^ cells, whereas overexpression of AQP5 significantly increased the proportion of CD44^+^/CD133^+^/LGR5^+^ cells. Together, these data suggest that AQP5 promotes the stemness of GC cells in vitro.

To determine the effect of AQP5 on the tumorigenicity of GC-CSCs, we established a xenograft model. Different concentrations of cells (8*10^2^–8*10^6^) were subcutaneously injected into severely immunodeficient mice. As shown in Fig. 3h-j and Table S5, knockdown of AQP5 significantly reduced the tumor formation rate of GC cells as well as the tumor weight and volume. In contrast, overexpression of AQP5 promoted the tumorigenicity of GC cells as well as the tumor weight and volume (Fig. 3k-m and Table S6). Taken together, these data indicate that AQP5 promotes the stemness and tumorigenicity of GC cells in vivo.

### Complementary effects of AQP5 and LGR5 on the tumorigenesis of GC-CSCs

**Fig. 4:**
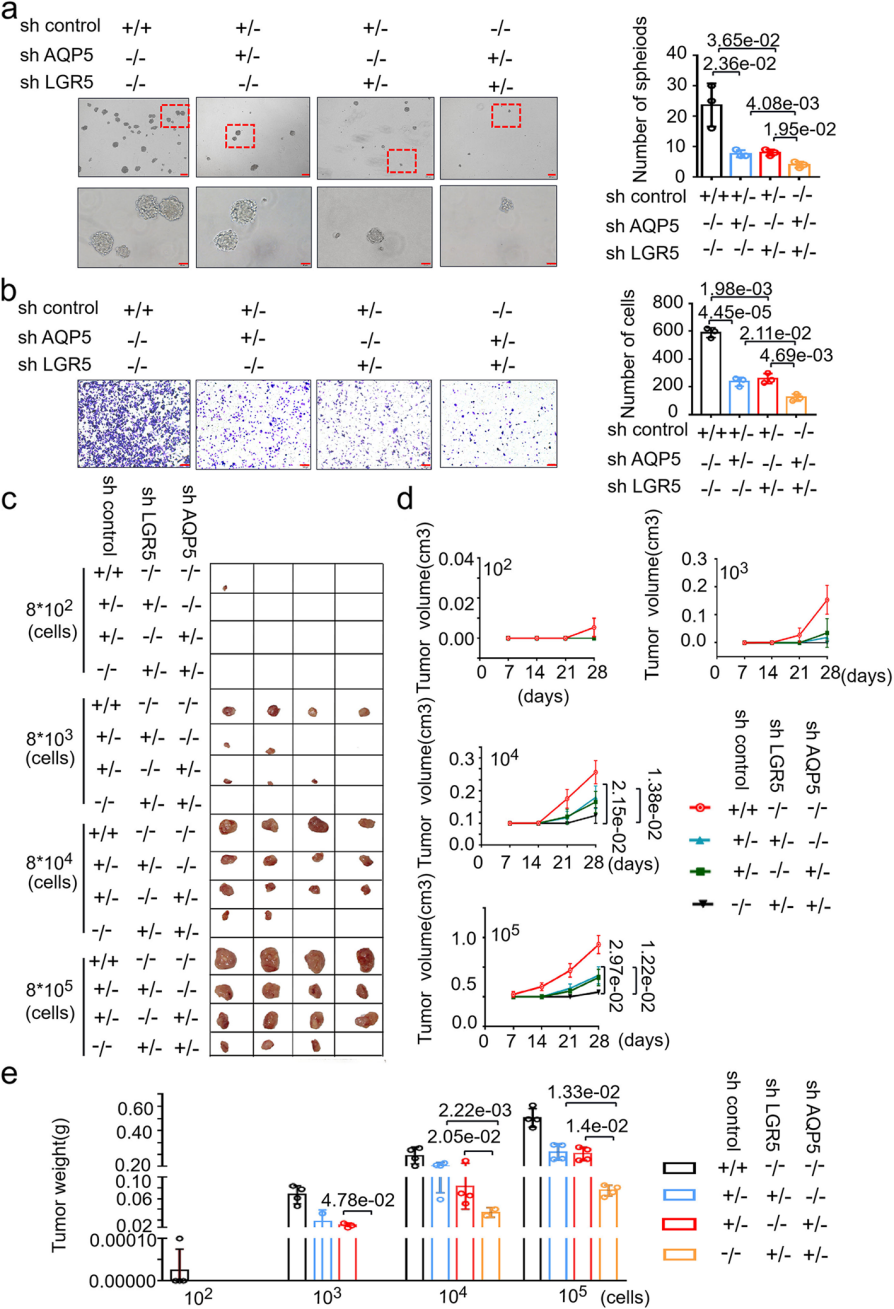
Co-knockdown of AQP5 and LGR5 significantly attenuates the malignant biological function of GC-CSCs. (a-e) AQP5 and/or LGR5 were knocked down in AGS cells, and the cells were analyzed to assess sphere formation in serum-free culture (**a**); cell migration (**b**); cells were diluted and subcutaneously injected into severely immunodeficient mice (**c**). Tumors were examined over a 28-day period (*n* = 4 for each group). The tumor volume (**d**) and tumor weight (**e**) were monitored in the indicated groups and at the indicated time points

Based on the fact that AQP5 and LGR5 are co-expressed in GC, we next investigate the functional relationship between AQP5 and LGR5. We knocked down AQP5 alone, LGR5 alone, or both in GC cells. Unexpectedly, the simultaneous knockdown of AQP5 and LGR5 significantly attenuated sphere formation and migration, whereas single shRNA treatment alone had only a moderate effect (Fig. 4a and b). And in cells that co-knockdown of both AQP5 and LGR5, the expression of LGR5 is significantly lower compared to either AQP5 knockdown or LGR5 knockdown (figure S6). Similar results were observed in vivo. As shown in Fig. 4c-e and Table S7, simultaneous knockdown of AQP5 and LGR5 significantly attenuated the tumorigenicity of GC-CSCs, and this effect was more potent than that after AQP5 or LGR5 was knocked down alone. Thus, the data indicate that AQP5 and LGR5 function synergistically to promote the tumorigenesis of GC-CSCs.

### AQP5 activates autophagy in GC-CSCs

**Fig. 5:**
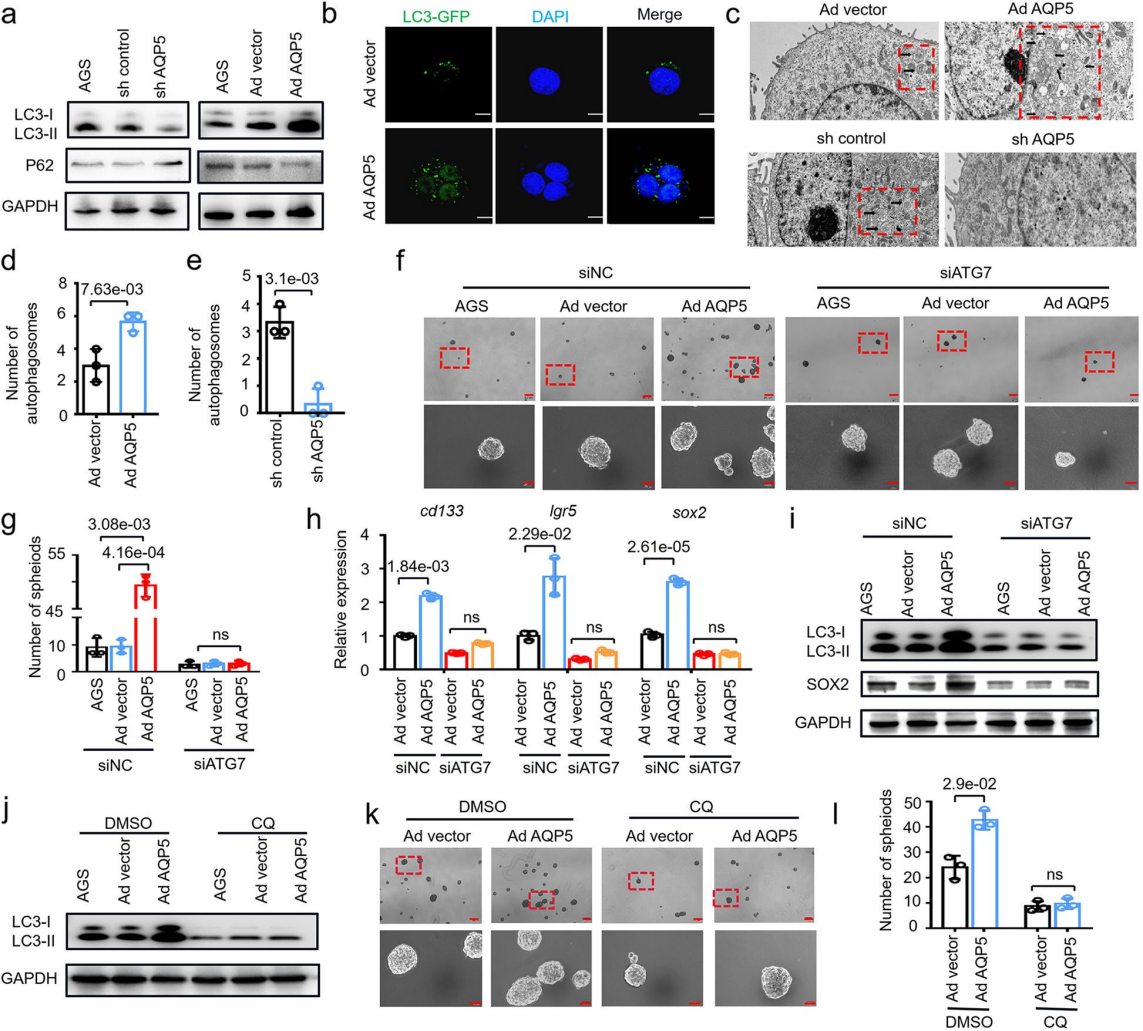
AQP5 activates GC-CSCs by inducing autophagy. **a** The expression levels of LC3 and p62 were evaluated in AQP5-knockdown or AQP5-overexpressing AGS cells. **b** AGS cells was infected with an adenovirus that expressed GFP-linked LC3 (GFP-LC3). Confocal microscopy was used to obtain fluorescent images. **c** TEM was used to assess autophagosome formation in AQP5-knockdown or AQP5-overexpressing AGS cells (red dashed boxes, black arrows indicate autophagolysosomal structures). **d** and **e** Statistical analysis of observed autophagosomes. **f** The spheroid-forming ability was evaluated after transfection of ATG7 siRNA in AQP5-overexpressing AGS cells. **g** Statistical analysis was performed on the number of spheroids (diameter > 50 μm). **h** The expression levels of cd133, lgr5 and sox2 in AQP5-overexpressing AGS cells were assessed after transfection with ATG7 siRNA. **i** Expression of LC3 and SOX2 in AQP5-overexpressing AGS cells was assessed after transfection of ATG7 siRNA. **j** LC3 expression in AGS cells overexpressing AQP5 was analyzed after CQ treatment. **k** The spheroid-forming of AGS cells overexpressing AQP5 was assessed after CQ treatment. **l** Statistical analysis was performed on the number of spheroids (diameter > 50 μm)

To elucidate the molecular mechanisms by which AQP5 regulates GC-CSCs, we performed transcriptome sequencing and proteomic analysis to analyze AQP5-regulated genes in GC-CSCs, and screened out differential genes for GSEA (Tables S8, S9, figure S7). Unexpectedly, we observed that no stem cell-related pathways were enriched; thus, we hypothesized that AQP5 may affect GC-CSCs through other mechanisms. AQP3, a member of the AQP5 homoprotein family, has been proven to promote tumor development by activating autophagy [22], which prompted us to hypothesize that AQP5 might regulate autophagy to determine the fates of GC-CSCs. Then, we examined the impacts of AQP5-mediated autophagy activation and observed that AQP5 knockdown suppressed LC3II expression and increased P62 expression, whereas overexpression of AQP5 enhanced LC3II expression and repressed P62 expression (Fig. 5a). Furthermore, we found that overexpression of AQP5 induced the formation of LC3 autophagosomes in GC-CSCs (Fig. 5b). Similarly, cell transmission electron microscopy revealed that AQP5 affected the number of autophagosomes in GC-CSCs (Fig. 5c-e). Thus, the data indicate that AQP5 activates autophagy in GC-CSCs.

We next examined whether AQP5-mediated autophagy impacted the stemness and self-renewal capacity of GC cells. To this end, we knocked down the key autophagy protein ATG7 in AQP5-overexpressing GC cells. First, knockdown of ATG7 inhibited the expression of LC3II and SOX2 (Figure S8). Then, ATG7 knockdown inhibited the AQP5-induced expression of LC3II. We further found that ATG7 knockdown significantly repressed the AQP5-induced self-renewal ability and expression of the stemness marker SOX2 (Fig. 5f-i). The autophagy inhibitor CQ was used to confirm this observation. We demonstrated that CQ indeed reversed the autophagy activation and self-renewal capacity induced by AQP5, including LC3II expression and sphere formation (Fig. 5j-l). Collectively, these results suggest that AQP5 regulates GC-CSC functions by impacting autophagy.

### *AQP5 directs GC-CSCs functions *via* K63-mediated ubiquitination of ULK1*

**Fig. 6:**
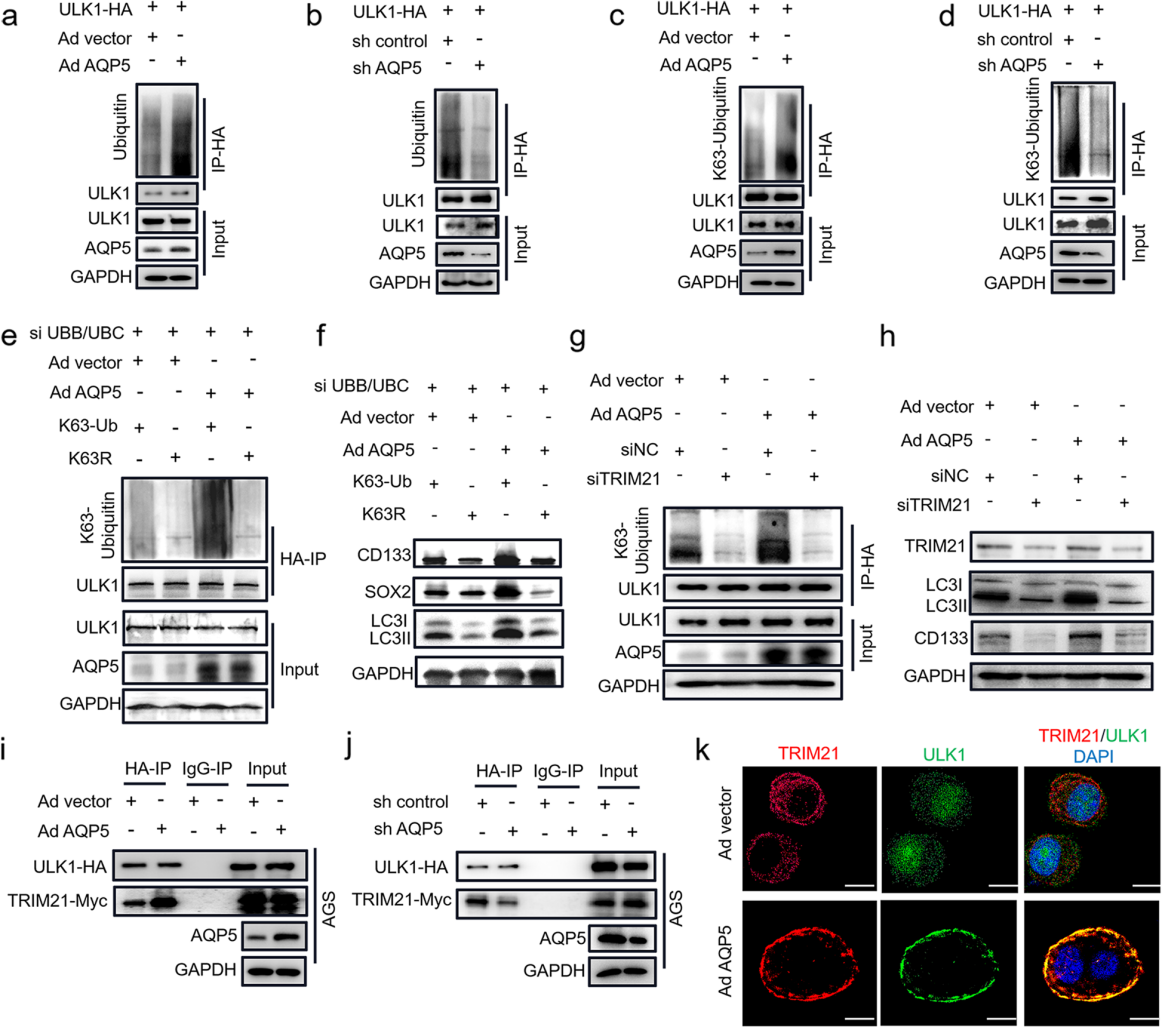
AQP5 promotes ULK1 ubiquitination via TRIM21. **a**-**d** AQP5 was overexpressed or knocked down in 293 T cells; and the cells were transfected with ULK1-HA for 48 h. The cell lysates were subjected to IP with anti-HA agarose and immunoblotted with the indicated antibodies. (e and f) AQP5 was overexpressed in AGS cells, and the cells were co-transfected with UBB/UBC siRNA, K63-Ub-HA or K63-R-HA. IP assay was performed with anti-HA agarose, followed by immunoblotting with the indicated antibodies (**e**); Cell lysates were analyzed by WB with the indicated antibodies (**h**). (**g** and **h**) AQP5 was overexpressed in AGS cells transfected with ULK1-HA and TRIM21 siRNA, and the cells were analyzed by IP assays with anti-HA agarose and immunoblotted with the indicated antibodies (**g**) or (**h**) analyzed by WB with the indicated antibodies. (**i** and **j**) AQP5-overexpressing (**i**) and AQP5-knockdown (j) AGS cells were co-transfected with ULK1-HA and TRIM21-Myc. Cell lysates were subjected to IP with anti-HA agarose and immunoblotted with the indicated antibodies. (**k**) AQP5-overexpressing AGS cells were co-transfected with ULK1-HA and TRIM21-Myc. Confocal microscopy was used to analyze the interaction between TRIM21 (red) and ULK1 (green)

To adress how AQP5 activates autophagy, we overexpressed AQP5 in AGS and 293 T cells. The results showed that overexpression of AQP5 did not impact key autophagy proteins, such as ULK1, BECLIN1, ATG5, ATG7, ATG12 and ATG16L1 (Figure S9a-f). Studies have shown that ubiquitination is a posttranslational modification that is essential for various intracellular processes, and it is involved in multiple aspects of autophagy, including the regulation of the initiation, execution, and termination of autophagy [23]. We hypothesized that AQP5 may promote autophagy activation in GC-CSCs by regulating the ubiquitination of key autophagy proteins. Notably, overexpression of AQP5 increased the ubiquitination of ULK1 without impacting the ubiquitination of other key autophagy proteins, including BECLIN1, ATG5, ATG7, ATG12 and ATG16L1 (Figure S9a-f). The findings were further validated in 293 T cells, as shown in Fig. 6a, b, overexpression of AQP5 significantly enhanced the ubiquitination of ULK1. In contrast, knockdown of AQP5 suppressed the ubiquitination of ULK1. The 7 lysines and N-terminal methionine in ubiquitin molecules can be further modified by ubiquitin molecules to form 8 types of ubiquitin chains. Among them, the K48, K63 and K27 ubiquitin chains are highly abundant and have been thoroughly studied [24]. Therefore, we further examined the K27-, K48- and K63-mediated ubiquitination of ULK1. As shown in Figure S8g-i, overexpression of AQP5 significantly promoted the K63-mediated ubiquitination of ULK1 but not the K27- or K48-mediated ubiquitination of ULK1. Similar results were observed in 293 T cells (Fig. 6c, d). Taken together, these data suggest that AQP5 regulates the ubiquitination of the key autophagy protein ULK1.

To further verify the effect of the ubiquitination of ULK1 on the role of AQP5 in regulating GC-CSCs, we knocked down the expression of endogenous ubiquitin and generated GC-CSCs that exogenously expressed HA-ubiquitin (K63-Ub) or K63R-mutant ubiquitin (K63R). The results showed that the K63-mediated ubiquitination of ULK1 was significantly inhibited after knocking down endogenous ubiquitin (Fig. 6e); while K63R-mutant ubiquitin significantly blocked the AQP5 overexpression-induced self-renewal and migration of GC cells (Figure S10a, b). Additionally, the K63R-mutant ubiquitin inhibited the AQP5 overexpression-induced upregulation of stemness markers, including CD133 and SOX2, and LC3II (Fig. 6f). Thus, the data suggest that AQP5 promotes the stemness and autophagy of GC-CSCs by regulating the K63-mediated ubiquitination of ULK1.

### AQP5 recruits TRIM21 to ULK1 and induces the ubiquitination of ULK1

To investigate how AQP5 activates the ubiquitination of ULK1, we analyzed proteins that can physically interact with AQP5 using mass spectrometry. TRIM21 was selected for further validation because this AQP5-binding protein is the only E3 ubiquitin ligase that is closely associated with ubiquitination (Table S10). Therefore, we hypothesized that AQP5 induced the ubiquitination of ULK1 via TRIM21. As expected, immunoprecipitation analyses revealed that AQP5, TRIM21 and ULK1 could bind to each other (Figure S11a-c). Moreover, TRIM21 knockdown inhibited the AQP5 overexpression-induced K63-mediated ubiquitination of ULK1 (Fig. 6g). Importantly, TRIM21 knockdown reversed the regulatory effect of AQP5 on GC-CSCs self-renewal and on the expression of the stemness marker CD133 and the key autophagy protein LC3II (Fig. 6h, Figure S12). The data indicate that AQP5 regulates the ubiquitination of ULK1 via TRIM21, promoting the autophagy and stemness of GC-CSCs.

Interestingly, AQP5 had no effect on the expression level of TRIM21 (Fig. 6h). We thus postulated that AQP5 promoted the ULK1-TRIM21 interaction. Overexpression of AQP5 significantly promoted the interaction of ULK1 and TRIM21, while knockdown of AQP5 attenuated the binding of ULK1 to TRIM21 (Fig. 6i, j and Figure S11d, e). The results were confirmed in the ULK1-TRIM21 fluorescence confocal assay (Fig. 6k). To further verify the regulation of the ULK1-TRIM21 interaction by AQP5, we overexpressed AQP5 in 293 T cells to different degrees. As shown in Figure S11f, elevated AQP5 expression significantly promoted ULK1-TRIM21 interaction and K63-mediated ubiquitination. These data suggest that AQP5 promotes the K63-mediated ubiquitination of ULK1 by recruiting the E3 ligase TRIM21 to ULK1.

## Discussion

In the present study, we identified and validated AQP5 as a novel specific surface marker of GC-CSCs by analyzing a cell atlas of GCs and GMs. To this end, we performed single-cell sequencing to probe the key marker genes of each cell cluster. By comparison with non-GC-CSCs, we found that AQP5 was a novel specific surface marker of GC-CSCs. At the functional level, we demonstrate that AQP5 promotes the self-renewal and tumorigenesis of GC-CSCs. Interestingly, AQP5 complements LGR5 to promote the tumorigenesis of GC-CSCs. The results suggest that CSCs express their own specific marker that reflects their own tumor origin, highlighting the importance of AQP5 as a specific marker of gastric cancer that could be targeted by potential novel therapeutic strategies. First, this study sheds new light on the novel biological functions of the membrane protein AQP5. Although previous studies reported AQP5 as a marker that is enriched in mouse and human adult pyloric stem cells [25], its biological functions in CSCs, especially in GC-CSCs, remain unknown. Our findings demonstrate that AQP5 is highly expressed in GCs and is clinically correlated with the progression of gastric cancer. The oncogenic role of AQP5 was functionally validated in several in vitro and in vivo experimental models. Downregulation of AQP5 markedly suppresses cell growth and tumor growth in cultured GC-CSCs and xenograft mouse models. By comparison with non-GC-CSCs, we identified and verified AQP5 as a novel specific marker of GC-CSCs, and AQP5 is co-expressed with the canonical stem cell markers LGR5. Functionally, we demonstrate that AQP5 promotes the self-renewal and tumorigenicity of GC-CSCs. Thus, the results consistently point to the notion that the AQP5 + cell compartment is an important tumor-initiating population.

Second, the present study suggests that AQP5 complements LGR5 and synergistically promotes the tumorigenesis of GC-CSCs. LGR5 is a well-characterized stem cell marker that is expressed in several tissues/organs, including the small intestine, colon, and liver. LGR5 + stem cells are involved in the process of oncogenesis, acting as tumor-initiating cells of intestinal cancer and fueling tumor growth [26]. Here, we demonstrate that AQP5 is specifically expressed in GC-CSCs and is involved in the regulation of these cells by LGR5. Our results indicate that co-knockdown of AQP5 and LGR5 substantially attenuates the self-renewal and tumorigenicity of GC-CSCs compared to knockdown of AQP5 or LGR5 alone. These results suggest that AQP5 coordinates with LGR5 to promote tumorigenesis through an unknown mechanism. Previous studies have shown that AQP5 + cells act as cells of origin for tumors [25]. Thus, we hypothesized that the AQP5 + /LGR5 + stem cell bank is the origin of gastric cancer. It is worth emphasizing that LGR5 and AQP5 are expressed in the same cells in both GCs and GMs. Interestingly, the expression of AQP5 in GC-CSCs is much higher than that in GM-CSCs. Cancer stem cell markers, such as LGR5 and CD133, are not able to distinguish CSCs from normal tissue stem cells. However, AQP5 can clearly distinguish these cell populations, suggesting that AQP5 is a more suitable target for the treatment of gastric cancer than LGR5.

Third, the results demonstrate that AQP5 is specially expressed in GC-CSCs rather than CSCs from tumors of other origins, which allows us to propose a novel concept that specific surface markers can identify the CSCs from tumors of individual origins, unlike conventional CSC markers. We found the following: 1. AQP5 is highly expressed in tissues from gastric cancer patients; 2. AQP5 is specifically expressed in GC-CSCs rather than GM-SCs or CSCs from tumors of other origins; and 3. AQP5 promotes the self-renewal and tumorigenicity of GC-CSCs. Thus, we propose that AQP5 is a specific marker for GC-CSCs. Our future study will focus on the identification of specific markers of CSCs from tumors of other origins.

Fourth, the present study reveals a previously unknown mechanism by which AQP5 regulates the autophagy and stemness of GC-CSCs. Autophagy is necessary to maintain the stemness of CSCs in various tumor types, and another aquaporin family member, AQP3, which has been shown to facilitate chemoresistance by stimulating autophagy [22]. Thus, we postulated that AQP5 may exert biological effects on GC-CSCs through autophagy. Indeed, AQP5 promotes autophagy in GC-CSCs, and knockdown of the key autophagy protein ATG7 or treatment with the autophagy inhibitor CQ reversed this effect. When we explored the mechanisms by which AQP5 affects the autophagy and stemness of GC-CSCs, we found the involvement of TRIM21. We revealed that AQP5 recruits TRIM21 to the key autophagy protein ULK1 and induces the ubiquitination of ULK1, thus activating autophagy and enhancing the stemness of GC-CSCs. This notion is supported by three lines of experimental evidence: (i) AQP5 directly binds to TRIM21 and ULK1; (ii) knockdown of TRIM21 reduces the interaction of AQP5 and ULK1 and reverses the activation of autophagy and self-renewal capacity induced by AQP5; and (iii) blocking the interaction between AQP5 and TRIM21 reverses the activation of autophagy and self-renewal capacity induced by AQP5. In accordance with our study, it has been reported that TRIM21 can act as an autophagy receptor, recruit and organize key components of the autophagic machinery, including ULK1, BECLIN1, and ATG16L1 [27]. AQP5 is embedded in the lipid bilayer of the cytoplasmic membrane and forms a tetramer. Due to the unique structure of AQP5, it plays an important role in the transmembrane transport of water and small molecular compounds [28]. Previous studies have reported that AQP5 cooperates with the calcium channel TRPV4 to regulate cell volume [29]. Moreover, AQP5 has also been shown to interact with the Na + /K + transporter ATP1A2 and the H + transporter ATP6V0A1 on the plasma membrane to regulate cells[30]. In addition, AQP5 can also interact with WNT2 [31] and PIP [32], however the specific mechanism has not been elucidated. Furthermore, we found that AQP5 can bind to TRIM21. Altogether, AQP5 can not only function as a channel protein but also involve in the ubiquitination modification via binding to E3 enzyme regulatory proteins.

## Conclusions

**Fig. 7:**
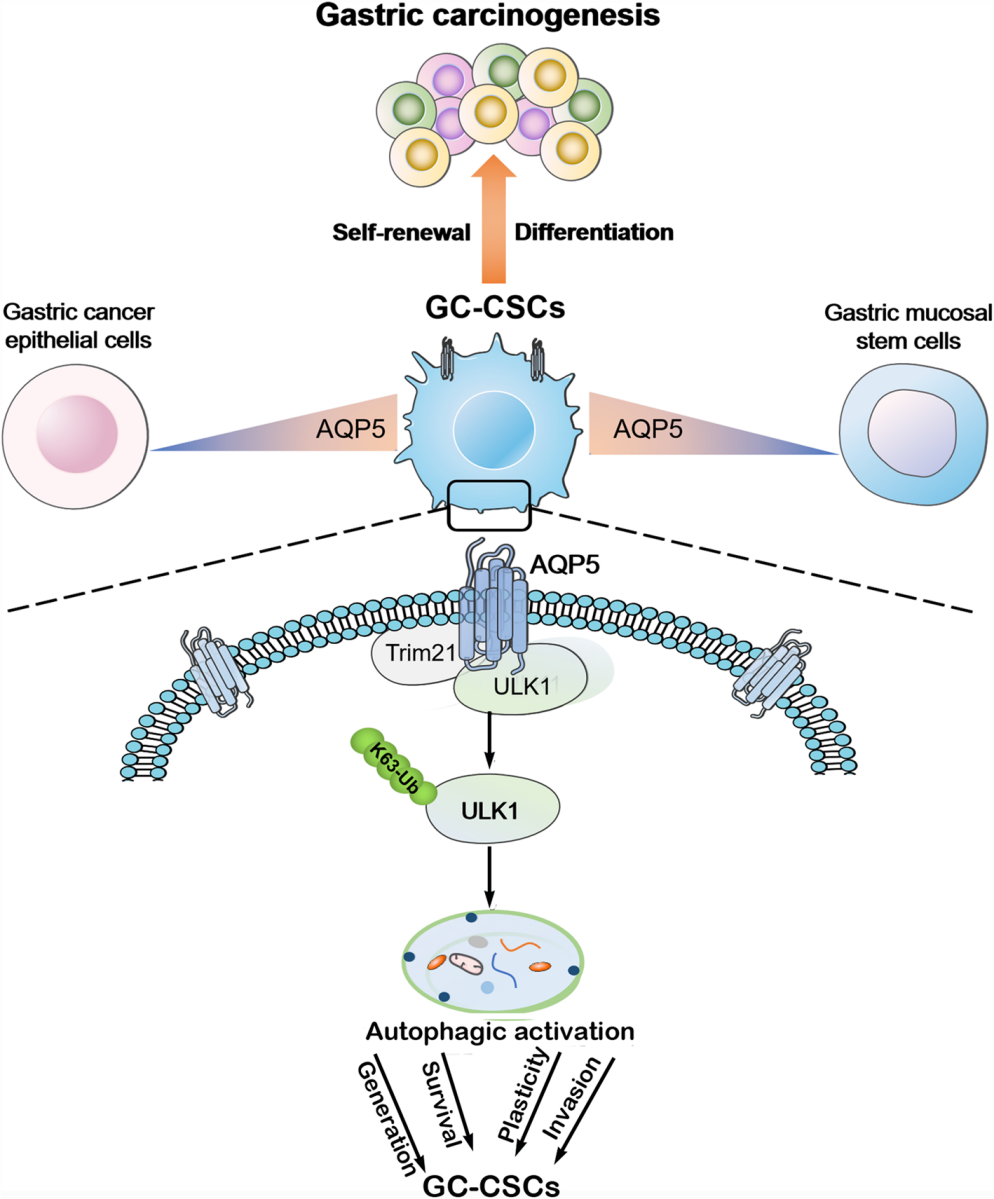
Schematic diagram of the study

We demonstrate novel biological functions of AQP5 in promoting gastric carcinogenesis. Our study proposes that AQP5 is a novel specific surface marker of GC-CSCs and identifies the mechanism by which AQP5 regulates the autophagy and malignant biological behavior of GC-CSCs (Fig. 7). These findings highlight that targeting AQP5 and its associated pathway could be an effective approach for CSC-based gastric cancer therapy.

## Supplementary Information

**Additional file 1: Figure S1.** Expression of marker genes in gastric cancer tissue epithelial/stem cellsand cultured adherent/spheroid cells. **Figure S2.** AQP5 expression in AGS/HGC-27/GES-1 spheroids and adherent cells. **Figure S3. **AQP5 promotes gastric cancer development in vitro and in vivo.** Figure S4. **Expression of AQP5 in GC-CSCs.** Figure S5. **AQP5 promotes the stemness of GC-CSCs.** Figure S6. **Effect of AQP5 on LGR5 expression.**Figure S7. **Cellular pathways affected by AQP5.** Figure S8. **ATG7 is the key regulator of GC cell autophagy.** Figure S9. **AQP5 affects key autophagy proteins.**Figure S10. **AQP5 promotes malignant behaviors of GC-CSCs by regulatingK63-mediated ubiquitination of ULK1.**Figure S11. **Interaction of AQP5, TRIM21 and ULK1.** Figure S12. **AQP5 promotes self-renewal via TRIM21 in GC-CSCs.

**Additional file 2: Supplementary Table 1.** Sequences ofreal-time PCR primers. **Supplementary Table 2.** Primary Antibodies. **Supplementary Table 3.** Second Antibodies. **Supplementary Table 4.** ShRNA or siRNA Oligonucleotides. **Supplementary Table 5.** Tumorigenicityof knockdown AQP5 and control group. **Supplementary Table 6.** Tumorigenicity of knockdown AQP5 and control group. **Supplementary Table 7.** Tumorigenicityof knockdown AQP5 and LGR5. **Supplementary Table 8.** Differentially expressed genes between exogenousoverexpression of AQP5 and control group. **Supplementary Table 9.** Differentially expressed genes between exogenousoverexpression of AQP5 and control group. **Supplementary Table 10.** Identification of the AQP5 protein complex by mass spectrometry.

**Additional file 3.**

## Data Availability

The datasets presented in this study can be found in online repositories. The names of the repository and accession number can be found below: GSE184198. The datasets for this study can be found in the TCGA (https://portal.gdc.cancer.gov/).

## Abbreviations

CSCs: Cancer stem cells
GC-CSCs: Gastric cancer stem cells
GC: Gastric cancer
GM-SCs: Gastric mucosa stem cells
GCs: Gastric cancer tissues
GMs: Gastric mucosa tissues
