# Correction: AQP5 complements LGR5 to determine the fates of gastric cancer stem cells through regulating ULK1 ubiquitination

**DOI:** 10.1186/s13046-022-02557-1

**Published:** 2022-12-13

**Authors:** Rou Zhao, Baoyu He, Qingli Bie, Jinghe Cao, Haoran Lu, Zhixin Zhang, Jing Liang, Li Wei, Huabao Xiong, Bin Zhang

**Affiliations:** 1 Department of Laboratory Medicine, Affiliated Hospital of Jining Medical University, Jining Medical University, Jining, Shandong People’s Republic of China; 2 Department of Hepatobiliary Surgery, Affiliated Hospital of Jining Medical University, Jining Medical University, Jining, Shandong People’s Republic of China; 3 Department of Gastrointestinal Surgery, Affiliated Hospital of Jining Medical University, Jining Medical University, Jining, Shandong People’s Republic of China; 4 Institute of Immunology and Molecular Medicine, Jining Medical University, Jining, Shandong People’s Republic of China; 5 Institute of Forensic Medicine and Laboratory Medicine, Jining Medical University, Jining, Shandong People’s Republic of China

**Correction: J ExpClin Cancer Res 41, 322 (2022)**

**https://doi.org/10.1186/s13046-022-02532-w**

Following the publication of the original article [1], one of the readers spotted an error in the supplementary materials. The Supplementary figures of Additional file 1 is missing and the e-files for Additional file 1 and Additional file 2 were interchanged.

The correction does not have any effect on the results or conclusions of the paper. The original article has been corrected.

## Supplementary Information

**Additional file1: Figure S1.** Expression of marker genes in gastric cancer tissue epithelial/stem cells and cultured adherent/spheroid cells. **Figure S2.** AQP5 expression in AGS/HGC-27/GES-1 spheroids and adherent cells. **Figure S3.** AQP5 promotes gastric cancer development in vitro and in vivo. **Figure S4.** Expression of AQP5 in GC-CSCs. **Figure S5.** AQP5 promotes the stemness of GC-CSCs. **Figure S6.** Effect of AQP5 on LGR5 expression. **Figure S7.** Cellular pathways affected by AQP5. **Figure S8.** ATG7 is the key regulator of GC cell autophagy. **Figure S9.** AQP5 affects key autophagy proteins. **Figure S10.** AQP5 promotes malignant behaviors of GC-CSCs by regulating K63-mediated ubiquitination of ULK1. **Figure S11.** Interaction of AQP5, TRIM21 and ULK1. **Figure S12.** AQP5 promotes self-renewal via TRIM21 in GC-CSCs.

**Additional file 2: Supplementary Table 1.** Sequences of real-time PCR primers. **Supplementary Table 2.** Primary Antibodies. **Supplementary Table 3.** Second Antibodies. **Supplementary Table 4.** ShRNA or siRNA Oligonucleotides. **Supplementary Table 5.** Tumorigenicity of knockdown AQP5 and control group. **Supplementary Table 6.** Tumorigenicity of knockdown AQP5 and control group. **Supplementary Table 7.** Tumorigenicity of knockdown AQP5 and LGR5. **Supplementary Table 8.** Differentially expressed genes between exogenous overexpression of AQP5 and control group. **Supplementary Table 9.** Differentially expressed genes between exogenous overexpression of AQP5 and control group. **Supplementary Table 10.** Identification of the AQP5 protein complex by mass spectrometry.

**Additional file 3.** 
