# Supplementary material for: AQP5 complements LGR5 to determine the fates of gastric cancer stem cells through regulating ULK1 ubiquitination
Source: J Exp Clin Cancer Res. 2022 Nov 14;41:322. doi: 10.1186/s13046-022-02532-w (PMC9661769; doi:10.1186/s13046-022-02532-w)
Supplement: Supplementary file 1 — Additional file 1: Figure S1. Expression of marker genes in gastric cancer tissue epithelial/stem cellsand cultured adherent/spheroid cells. Figure S2. AQP5 expression in AGS/HGC-27/GES-1 spheroids and adherent cells. Figure S3. AQP5 promotes gastric cancer development in vitro and in vivo. Figure S4. Expression of AQP5 in GC-CSCs. Figure S5. AQP5 promotes the stemness of GC-CSCs. Figure S6. Effect of AQP5 on LGR5 expression.Figure S7. Cellular pathways affected by AQP5. Figure S8. ATG7 is the key regulator of GC cell autophagy. Figure S9. AQP5 affects key autophagy proteins.Figure S10. AQP5 promotes malignant behaviors of GC-CSCs by regulatingK63-mediated ubiquitination of ULK1.Figure S11. Interaction of AQP5, TRIM21 and ULK1. Figure S12. AQP5 promotes self-renewal via TRIM21 in GC-CSCs. [file 13046_2022_2532_MOESM1_ESM.zip › 13046_2022_2532_MOESM1_ESM/13046_2022_2532_MOESM1_ESM.docx]

**Supplementary Figure legend**

**Figure S1. Expression of marker genes in gastric cancer tissue epithelial/stem cells and cultured adherent/spheroid cells**

(a and b) Gastric cancer tissue epithelial cells were sorted using Anti-EPCAM Micobeads. Stem cells were sorted using Anti-EPCAM Micobeads and Anti-CD133 Micobeads or Anti-LGR5 Micobeads. qRT‒PCR was used to assess the expression of cd133 (a) or lgr5 (b). (c and d) lgr5 is a stemness marker, and ck18 is a differentiation marker. AGS (c), HGC-27 (d) and GES-1 (e) cells were cultured as monolayers or under serum-free conditions as spheres. qRT‒PCR was used to assess the expression of lgr5 and ck18.

**Figure S2. AQP5 expression in AGS/HGC-27/GES-1 spheroids and adherent cells**

(a) AGS, HGC-27 and GES-1 cells were cultured as monolayers or under serum-free conditions as spheres. The expression of AQP5 was measured by WB, with GAPDH as the internal reference. (b) HCT116, HUH7, HEPG2 and A549 cells were cultured as monolayers or under serum-free conditions as spheres. qRT‒PCR was used to assess the expression of lgr5, oct4, cd133. (c) Expression levels of the five candidate genes were measured in cells sorted with the EPCAM and CD133 markers.

**Figure S3. AQP5 promotes gastric cancer development in vitro and in vivo**

1. The protein levels of AQP5 were evaluated in 10 paired GCs and GMs by Western blotting analyses. (b-e) AQP5 was knocked down or overexpressed in the AGS or HGC-27 cell lines, and the cells were analyzed to measure cell proliferation (b-e); cell migration (f, g); colony formation (h, i) and xenograft tumor (4 tumors/group) growth (j and k) The weight and volume of xenograft tumors were measured. Immunohistochemical staining of CK18, LGR5, CD133 and KI67 in xenograft tumors (i, m).

**Figure S4. Expression of AQP5 in GC-CSCs**

(a) The correlation between AQP5 transcript levels and CD133, OCT4, ALDH1A1 or CD24 mRNA levels was measured in the TCGA dataset. (b) Workflow. Spheroids were reseeded in medium supplemented with 10% FBS and cultured for 1 or 7 days. Then, RNA and protein fractions were harvested at the indicated time points. (c and d) Representative flow cytometric results for Figure 2f and Figure 2g. (e) Representative flow cytometric results for Figure S4f. (f) Statistical analysis of the proportion of AQP5^+^ cells in the LGR5^+^ subgroup of GCs and GMs. (g and h) Representative flow cytometric results of the expression of AQP5 and LGR5 in HGC-27 (g) (Figure 2h) or AGS (h) (Figure 2i) and GES-1 cells. (i) Representative flow cytometric results of the expression of CD133 and LGR5 in AGS and GES-1 cells (Figure 2j).

**Figure S5. AQP5 promotes the stemness of GC-CSCs**

1. c) Exogenous AQP5-overexpressing or AQP5-knockdown HGC-27 cells were cultured in serum-free medium for 10 days. Statistical analysis of the number of spheroids (diameter > 50 μm). (d and e) qRT‒PCR was performed to measure lgr5, sox2 and aqp5 expression in AQP5-overexpressing (d) or AQP5-knockdown, LGR5-knockdown (e) AGS cells. (f-h) Representative flow cytometry results of CD133 (f), LGR5 (g) and CD44 (h) expression in AQP5-overexpressing or AQP5-knockdown HGC-27 cells (correlated with Figure 3e-g).

**Figure S6. Effect of AQP5 on LGR5 expression**

AQP5 and/or LGR5 were knocked down in AGS cells, the expression levels of LGR5 were measured using qPCR(a) or WB(b) analyses.

**Figure S7 Cellular pathways affected by AQP5**

RNA sequencing of AQP5-related genes, GESA plots of genes affected by AQP5 knockdown (a) or AQP5 overexpression (b). (c) AQP5 was overexpressed in AGS cells, and cell lysates were used for proteome detection. GESA plots of protein affected by AQP5 overexpression.

**Figure S8 ATG7 is the key regulator of GC cell autophagy**

The protein levels of ATG7, LC3 and SOX2 were measured in AGS cells transfected with ATG7 siRNA.

**Figure S9 AQP5 affects key autophagy proteins**

(a-f) AQP5 was overexpressed in AGS cells. IP assays were performed with anti-ULK1 (a), anti-BECLIN1 (b), anti-ATG5 (c), anti-ATG7 (d), anti-ATG12 (e) or anti-ATG16L1 (f) antibodies, followed by immunoblotting with the indicated antibodies. (i-k) AQP5 was overexpressed in 293T cells, and the cells were transfected with ULK1-HA for 48 hours. The cell lysates were subjected to IP with anti-HA agarose and immunoblotted with the indicated antibodies.

**Figure S10 AQP5 promotes malignant behaviors of GC-CSCs by regulating K63-mediated ubiquitination of ULK1**

(a and b) AQP5 was overexpressed in AGS cells, and the cells were co-transfected with UBB/UBC siRNA, K63-Ub-HA or K63-R-HA. Cells were cultured to assess spheres formation (diameter > 50 μm) (a) and cell migratory ability (b).

**Figure S11 Interaction of AQP5, TRIM21 and ULK1**

1. c) 293T cells were cotransfected with AQP5-Flag, TRIM21-Myc and ULK1-HA plasmids. The cell lysates were subjected to an IP assay with anti-Flag, anti-Myc or anti-HA agarose, followed by immunoblotting with the indicated antibodies. (d and e) AQP5 was overexpressed (d) or knocked down (e) in 293T cells, and the cells were transfected with TRIM21-Myc and ULK1-HA plasmids. An IP assay was performed with anti-HA agarose, followed by immunoblotting with the indicated antibodies. (f) Gradient overexpression of AQP5 in 293T cells co-transfected with TRIM21-Myc and ULK1-HA. The cell lysates were subjected to an IP assay with anti-HA agarose and immunoblotted with the indicated antibodies.

**Figure S12 AQP5 promotes self-renewal via TRIM21 in GC-CSCs**

(a and b) The spheroid-forming ability of AQP5-overexpressing AGS cells was evaluated after transfection of TRIM21 siRNA. (diameter > 50 μm).
