# Supplementary figures and images for: AQP5 complements LGR5 to determine the fates of gastric cancer stem cells through regulating ULK1 ubiquitination
Source: J Exp Clin Cancer Res. 2022 Nov 14;41:322. doi: 10.1186/s13046-022-02532-w (PMC9661769; doi:10.1186/s13046-022-02532-w)

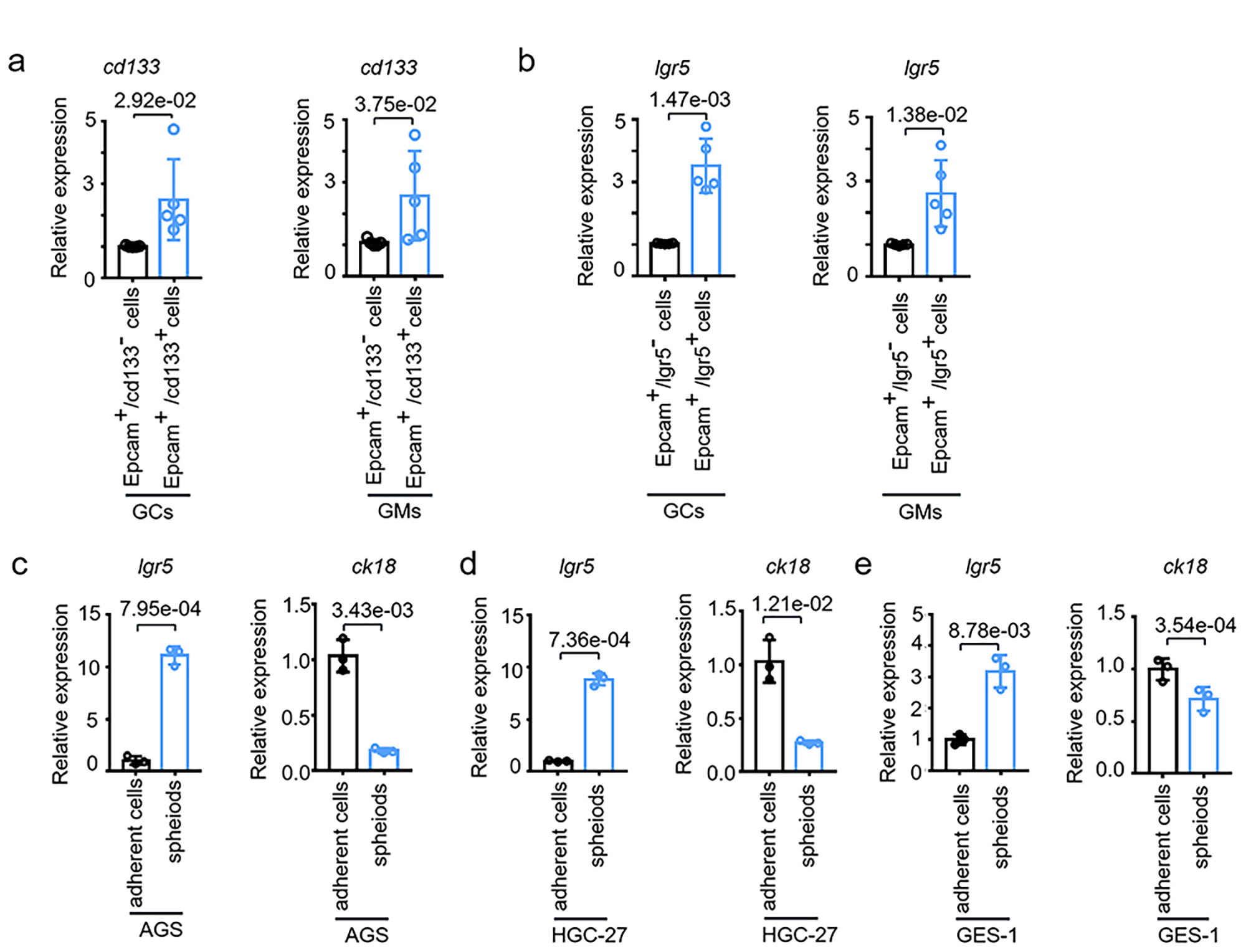

Supplement: Supplementary file 1 — Additional file 1: Figure S1. Expression of marker genes in gastric cancer tissue epithelial/stem cellsand cultured adherent/spheroid cells. Figure S2. AQP5 expression in AGS/HGC-27/GES-1 spheroids and adherent cells. Figure S3. AQP5 promotes gastric cancer development in vitro and in vivo. Figure S4. Expression of AQP5 in GC-CSCs. Figure S5. AQP5 promotes the stemness of GC-CSCs. Figure S6. Effect of AQP5 on LGR5 expression.Figure S7. Cellular pathways affected by AQP5. Figure S8. ATG7 is the key regulator of GC cell autophagy. Figure S9. AQP5 affects key autophagy proteins.Figure S10. AQP5 promotes malignant behaviors of GC-CSCs by regulatingK63-mediated ubiquitination of ULK1.Figure S11. Interaction of AQP5, TRIM21 and ULK1. Figure S12. AQP5 promotes self-renewal via TRIM21 in GC-CSCs. [file 13046_2022_2532_MOESM1_ESM.zip › 13046_2022_2532_MOESM1_ESM/figure S1.tif]

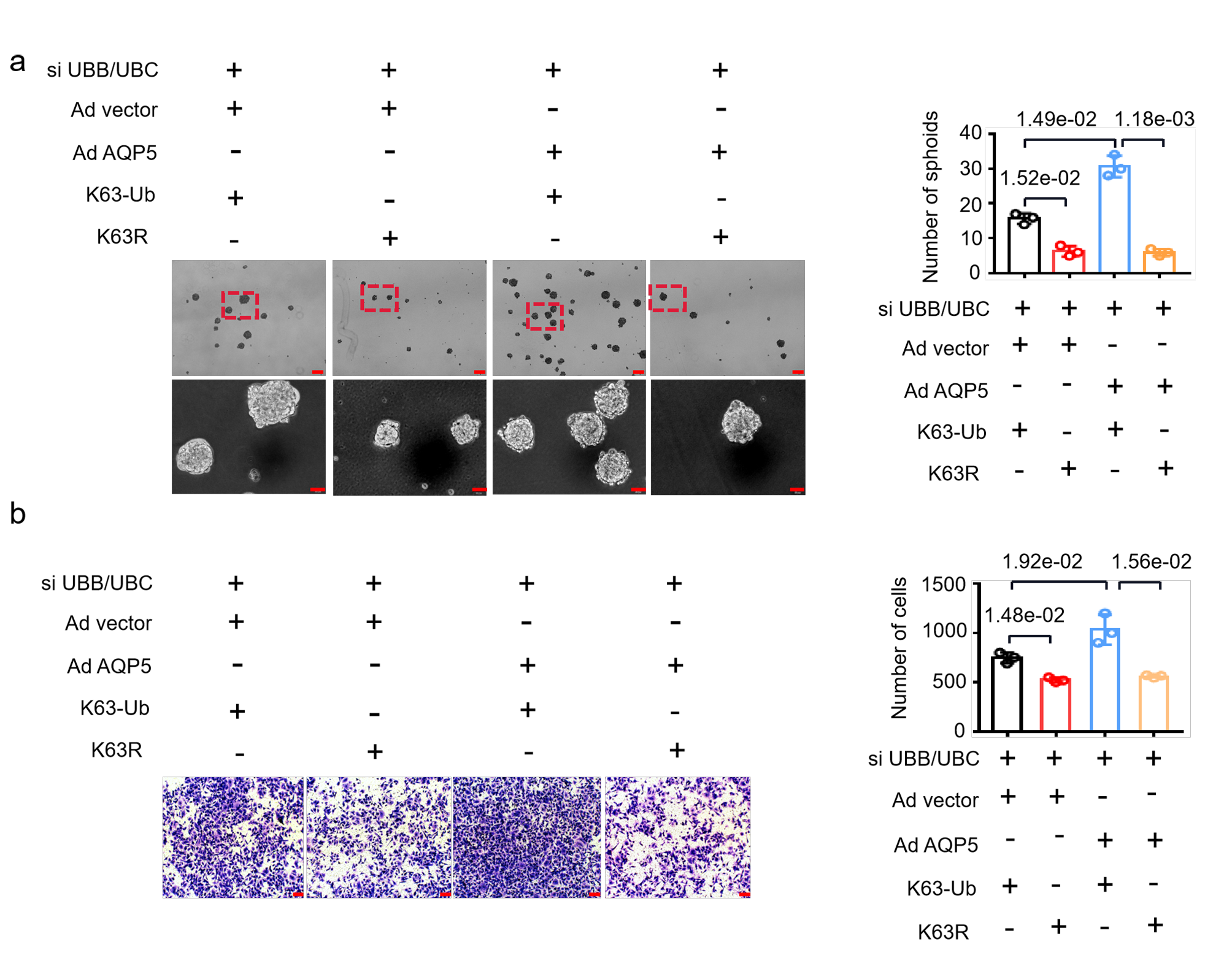

Supplement: Supplementary file 1 — Additional file 1: Figure S1. Expression of marker genes in gastric cancer tissue epithelial/stem cellsand cultured adherent/spheroid cells. Figure S2. AQP5 expression in AGS/HGC-27/GES-1 spheroids and adherent cells. Figure S3. AQP5 promotes gastric cancer development in vitro and in vivo. Figure S4. Expression of AQP5 in GC-CSCs. Figure S5. AQP5 promotes the stemness of GC-CSCs. Figure S6. Effect of AQP5 on LGR5 expression.Figure S7. Cellular pathways affected by AQP5. Figure S8. ATG7 is the key regulator of GC cell autophagy. Figure S9. AQP5 affects key autophagy proteins.Figure S10. AQP5 promotes malignant behaviors of GC-CSCs by regulatingK63-mediated ubiquitination of ULK1.Figure S11. Interaction of AQP5, TRIM21 and ULK1. Figure S12. AQP5 promotes self-renewal via TRIM21 in GC-CSCs. [file 13046_2022_2532_MOESM1_ESM.zip › 13046_2022_2532_MOESM1_ESM/figure S10.tif]

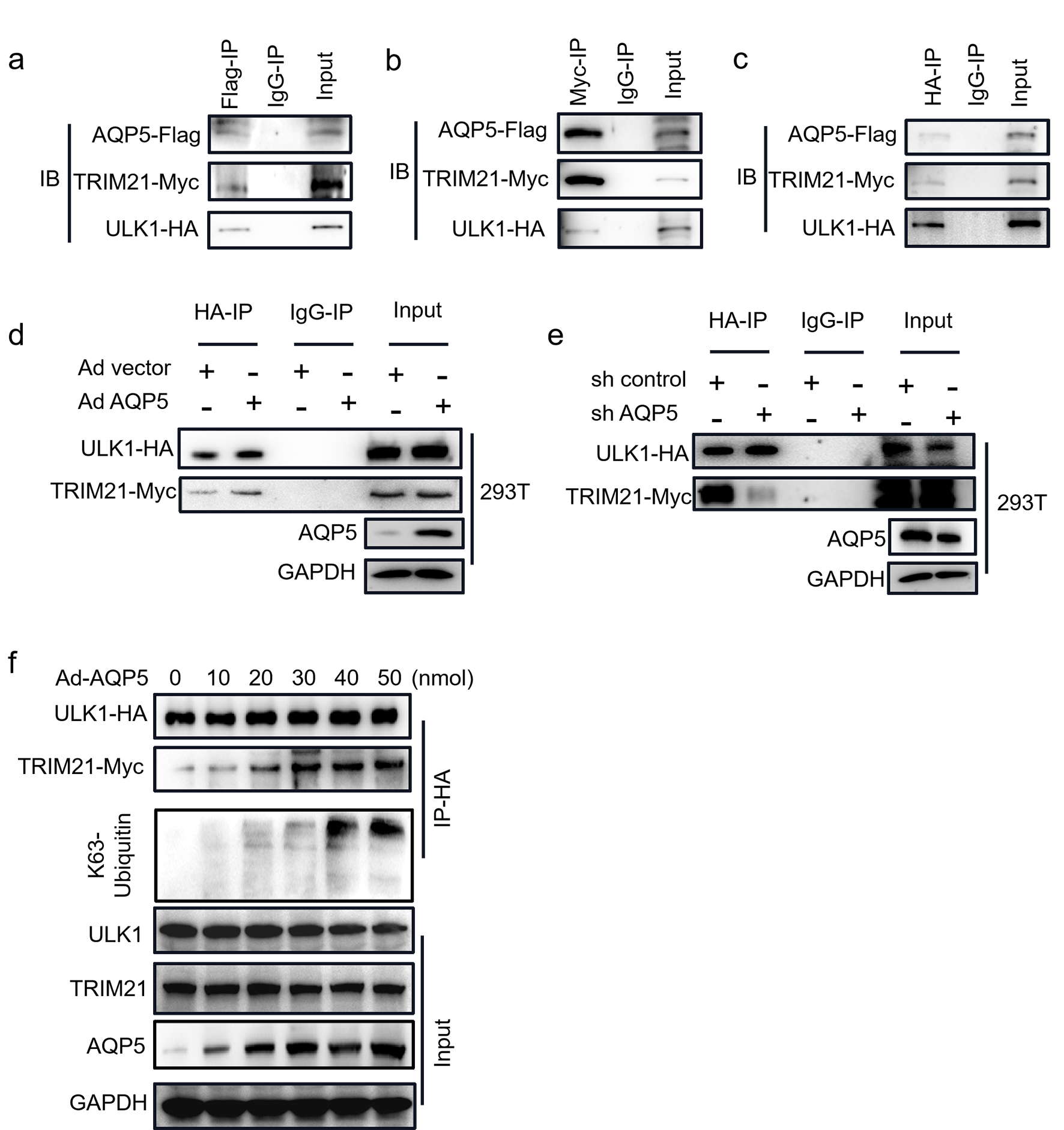

Supplement: Supplementary file 1 — Additional file 1: Figure S1. Expression of marker genes in gastric cancer tissue epithelial/stem cellsand cultured adherent/spheroid cells. Figure S2. AQP5 expression in AGS/HGC-27/GES-1 spheroids and adherent cells. Figure S3. AQP5 promotes gastric cancer development in vitro and in vivo. Figure S4. Expression of AQP5 in GC-CSCs. Figure S5. AQP5 promotes the stemness of GC-CSCs. Figure S6. Effect of AQP5 on LGR5 expression.Figure S7. Cellular pathways affected by AQP5. Figure S8. ATG7 is the key regulator of GC cell autophagy. Figure S9. AQP5 affects key autophagy proteins.Figure S10. AQP5 promotes malignant behaviors of GC-CSCs by regulatingK63-mediated ubiquitination of ULK1.Figure S11. Interaction of AQP5, TRIM21 and ULK1. Figure S12. AQP5 promotes self-renewal via TRIM21 in GC-CSCs. [file 13046_2022_2532_MOESM1_ESM.zip › 13046_2022_2532_MOESM1_ESM/figure S11.tif]

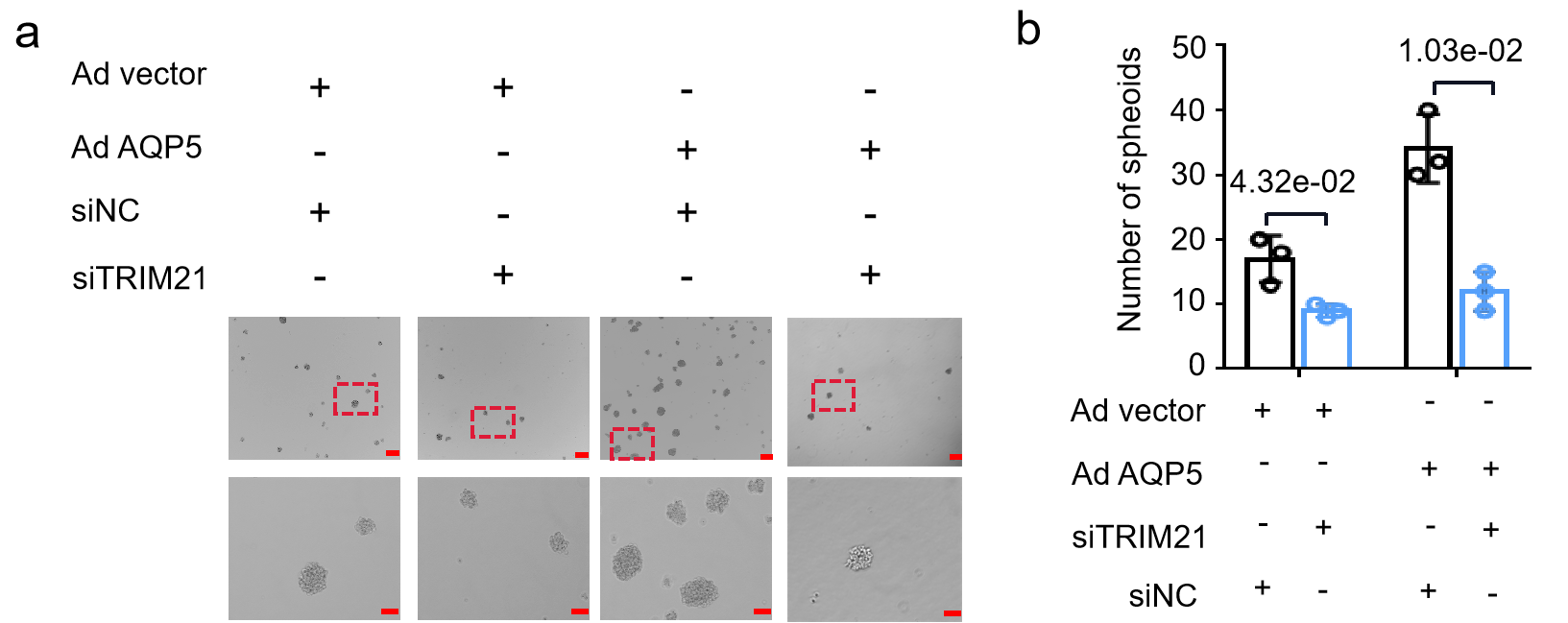

Supplement: Supplementary file 1 — Additional file 1: Figure S1. Expression of marker genes in gastric cancer tissue epithelial/stem cellsand cultured adherent/spheroid cells. Figure S2. AQP5 expression in AGS/HGC-27/GES-1 spheroids and adherent cells. Figure S3. AQP5 promotes gastric cancer development in vitro and in vivo. Figure S4. Expression of AQP5 in GC-CSCs. Figure S5. AQP5 promotes the stemness of GC-CSCs. Figure S6. Effect of AQP5 on LGR5 expression.Figure S7. Cellular pathways affected by AQP5. Figure S8. ATG7 is the key regulator of GC cell autophagy. Figure S9. AQP5 affects key autophagy proteins.Figure S10. AQP5 promotes malignant behaviors of GC-CSCs by regulatingK63-mediated ubiquitination of ULK1.Figure S11. Interaction of AQP5, TRIM21 and ULK1. Figure S12. AQP5 promotes self-renewal via TRIM21 in GC-CSCs. [file 13046_2022_2532_MOESM1_ESM.zip › 13046_2022_2532_MOESM1_ESM/figure S12.tif]

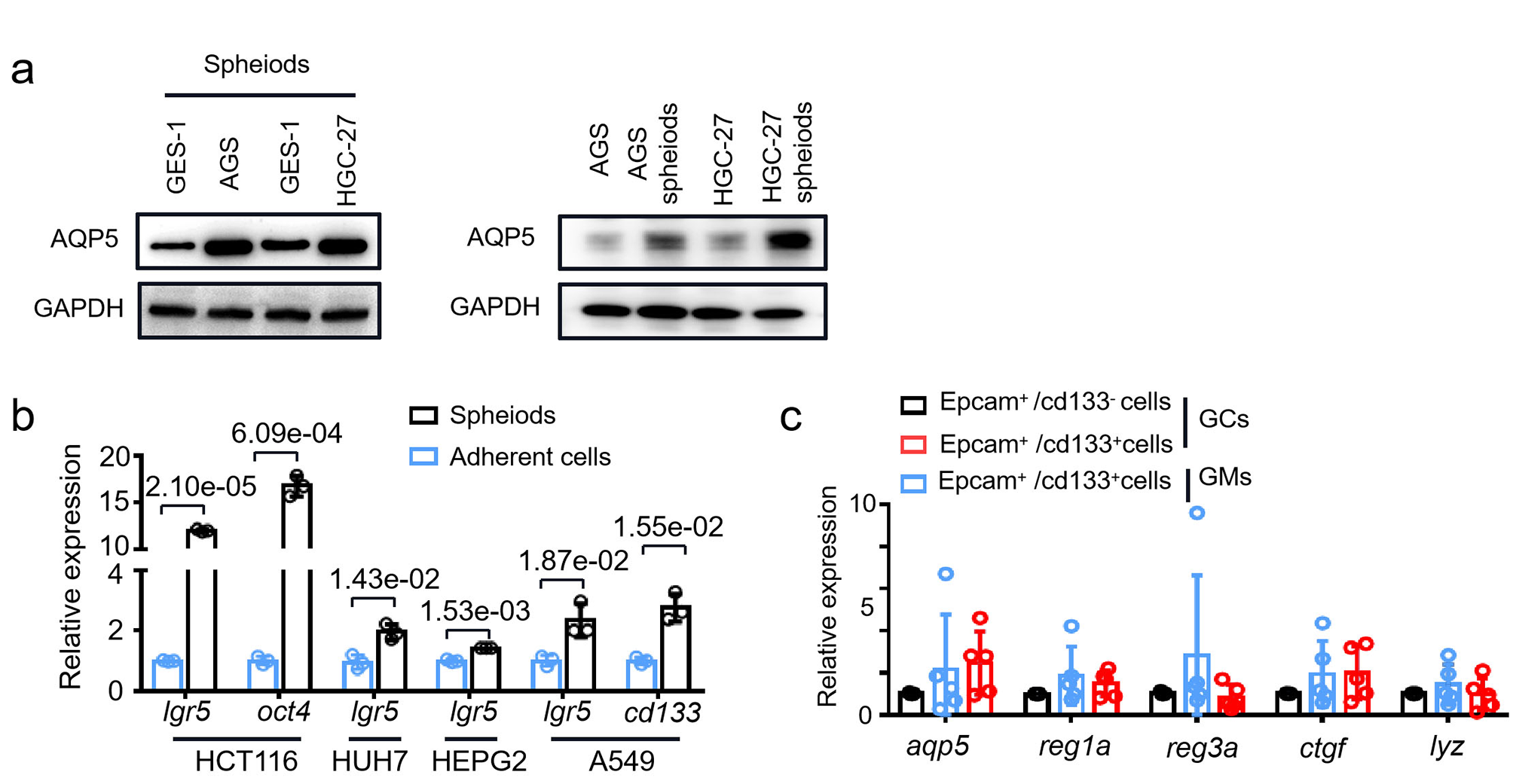

Supplement: Supplementary file 1 — Additional file 1: Figure S1. Expression of marker genes in gastric cancer tissue epithelial/stem cellsand cultured adherent/spheroid cells. Figure S2. AQP5 expression in AGS/HGC-27/GES-1 spheroids and adherent cells. Figure S3. AQP5 promotes gastric cancer development in vitro and in vivo. Figure S4. Expression of AQP5 in GC-CSCs. Figure S5. AQP5 promotes the stemness of GC-CSCs. Figure S6. Effect of AQP5 on LGR5 expression.Figure S7. Cellular pathways affected by AQP5. Figure S8. ATG7 is the key regulator of GC cell autophagy. Figure S9. AQP5 affects key autophagy proteins.Figure S10. AQP5 promotes malignant behaviors of GC-CSCs by regulatingK63-mediated ubiquitination of ULK1.Figure S11. Interaction of AQP5, TRIM21 and ULK1. Figure S12. AQP5 promotes self-renewal via TRIM21 in GC-CSCs. [file 13046_2022_2532_MOESM1_ESM.zip › 13046_2022_2532_MOESM1_ESM/figure S2.tif]

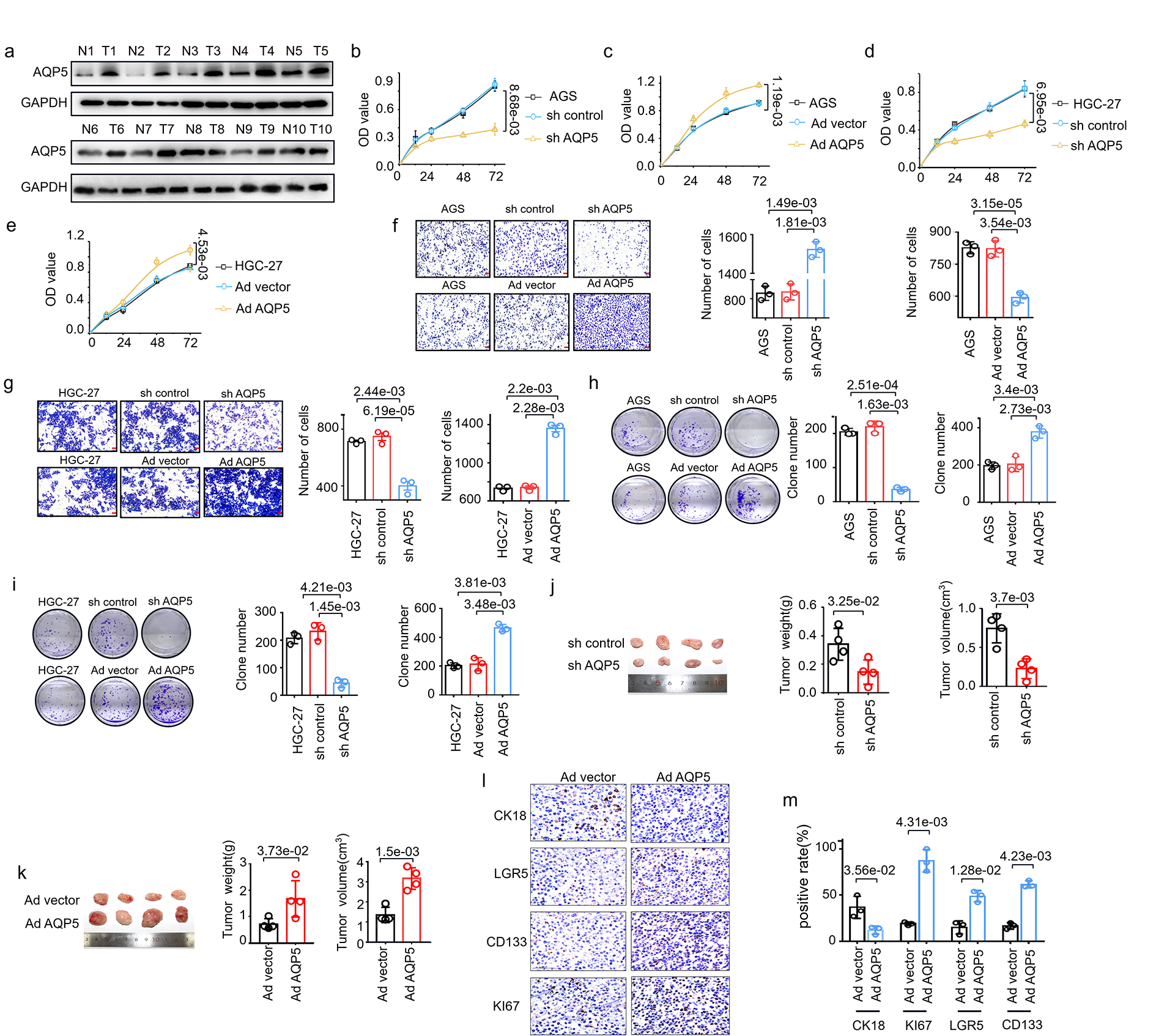

Supplement: Supplementary file 1 — Additional file 1: Figure S1. Expression of marker genes in gastric cancer tissue epithelial/stem cellsand cultured adherent/spheroid cells. Figure S2. AQP5 expression in AGS/HGC-27/GES-1 spheroids and adherent cells. Figure S3. AQP5 promotes gastric cancer development in vitro and in vivo. Figure S4. Expression of AQP5 in GC-CSCs. Figure S5. AQP5 promotes the stemness of GC-CSCs. Figure S6. Effect of AQP5 on LGR5 expression.Figure S7. Cellular pathways affected by AQP5. Figure S8. ATG7 is the key regulator of GC cell autophagy. Figure S9. AQP5 affects key autophagy proteins.Figure S10. AQP5 promotes malignant behaviors of GC-CSCs by regulatingK63-mediated ubiquitination of ULK1.Figure S11. Interaction of AQP5, TRIM21 and ULK1. Figure S12. AQP5 promotes self-renewal via TRIM21 in GC-CSCs. [file 13046_2022_2532_MOESM1_ESM.zip › 13046_2022_2532_MOESM1_ESM/figure S3.tif]

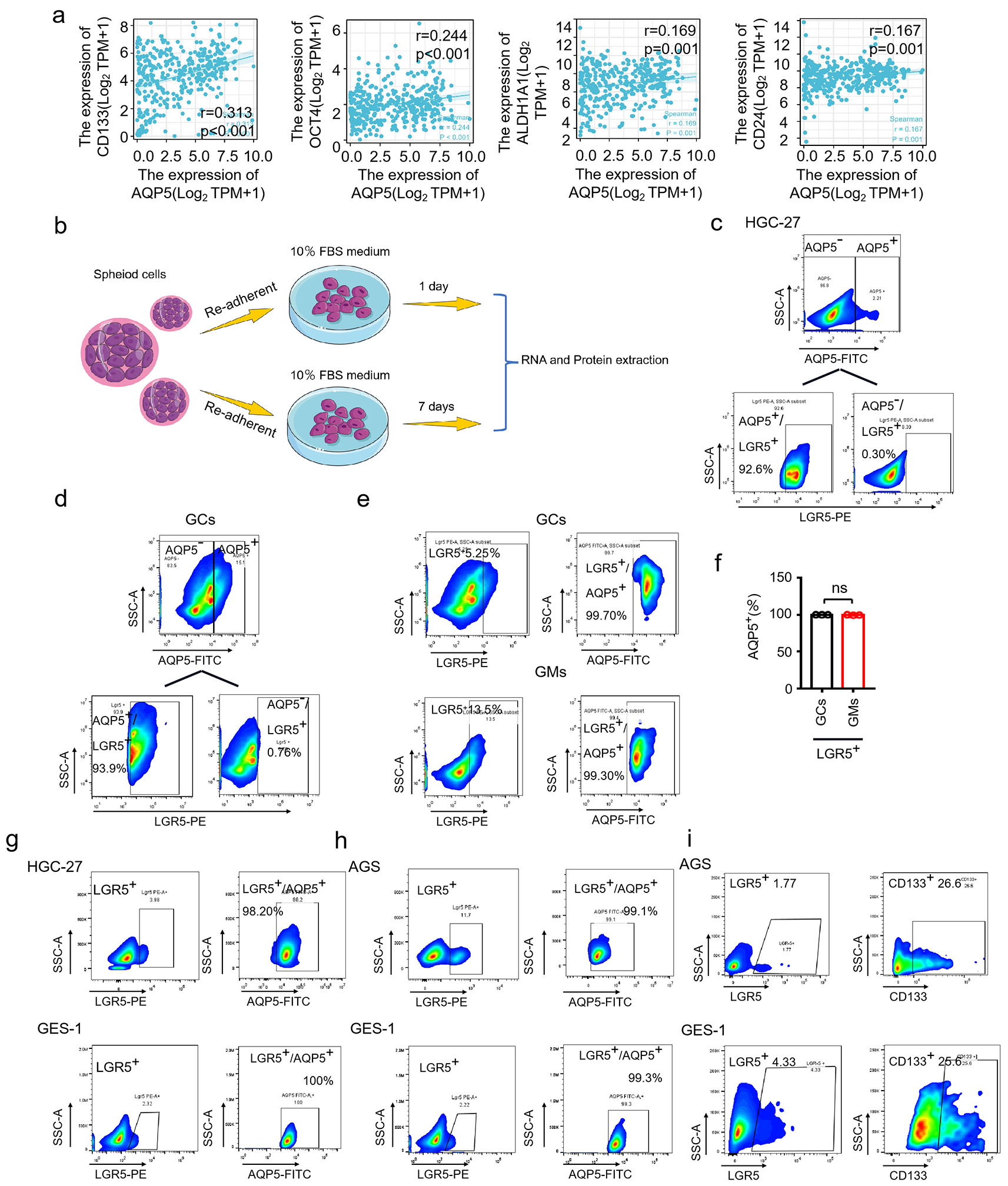

Supplement: Supplementary file 1 — Additional file 1: Figure S1. Expression of marker genes in gastric cancer tissue epithelial/stem cellsand cultured adherent/spheroid cells. Figure S2. AQP5 expression in AGS/HGC-27/GES-1 spheroids and adherent cells. Figure S3. AQP5 promotes gastric cancer development in vitro and in vivo. Figure S4. Expression of AQP5 in GC-CSCs. Figure S5. AQP5 promotes the stemness of GC-CSCs. Figure S6. Effect of AQP5 on LGR5 expression.Figure S7. Cellular pathways affected by AQP5. Figure S8. ATG7 is the key regulator of GC cell autophagy. Figure S9. AQP5 affects key autophagy proteins.Figure S10. AQP5 promotes malignant behaviors of GC-CSCs by regulatingK63-mediated ubiquitination of ULK1.Figure S11. Interaction of AQP5, TRIM21 and ULK1. Figure S12. AQP5 promotes self-renewal via TRIM21 in GC-CSCs. [file 13046_2022_2532_MOESM1_ESM.zip › 13046_2022_2532_MOESM1_ESM/figure S4.tif]

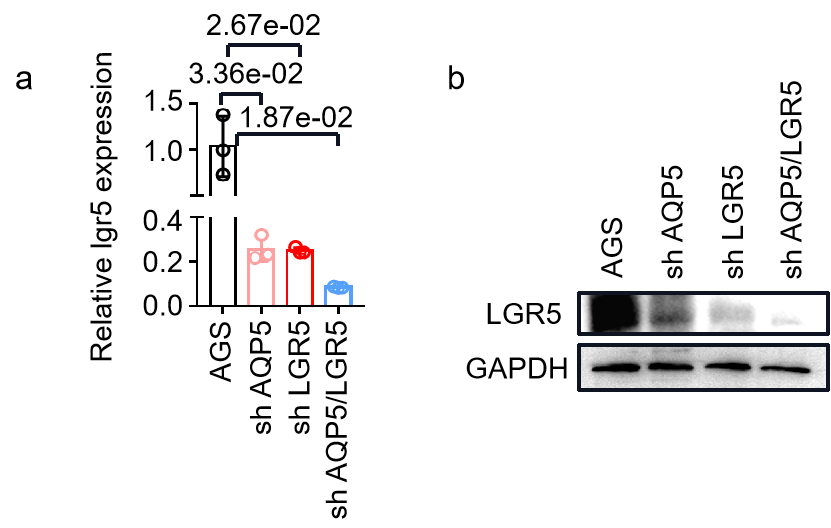

Supplement: Supplementary file 1 — Additional file 1: Figure S1. Expression of marker genes in gastric cancer tissue epithelial/stem cellsand cultured adherent/spheroid cells. Figure S2. AQP5 expression in AGS/HGC-27/GES-1 spheroids and adherent cells. Figure S3. AQP5 promotes gastric cancer development in vitro and in vivo. Figure S4. Expression of AQP5 in GC-CSCs. Figure S5. AQP5 promotes the stemness of GC-CSCs. Figure S6. Effect of AQP5 on LGR5 expression.Figure S7. Cellular pathways affected by AQP5. Figure S8. ATG7 is the key regulator of GC cell autophagy. Figure S9. AQP5 affects key autophagy proteins.Figure S10. AQP5 promotes malignant behaviors of GC-CSCs by regulatingK63-mediated ubiquitination of ULK1.Figure S11. Interaction of AQP5, TRIM21 and ULK1. Figure S12. AQP5 promotes self-renewal via TRIM21 in GC-CSCs. [file 13046_2022_2532_MOESM1_ESM.zip › 13046_2022_2532_MOESM1_ESM/figure S6.tif]

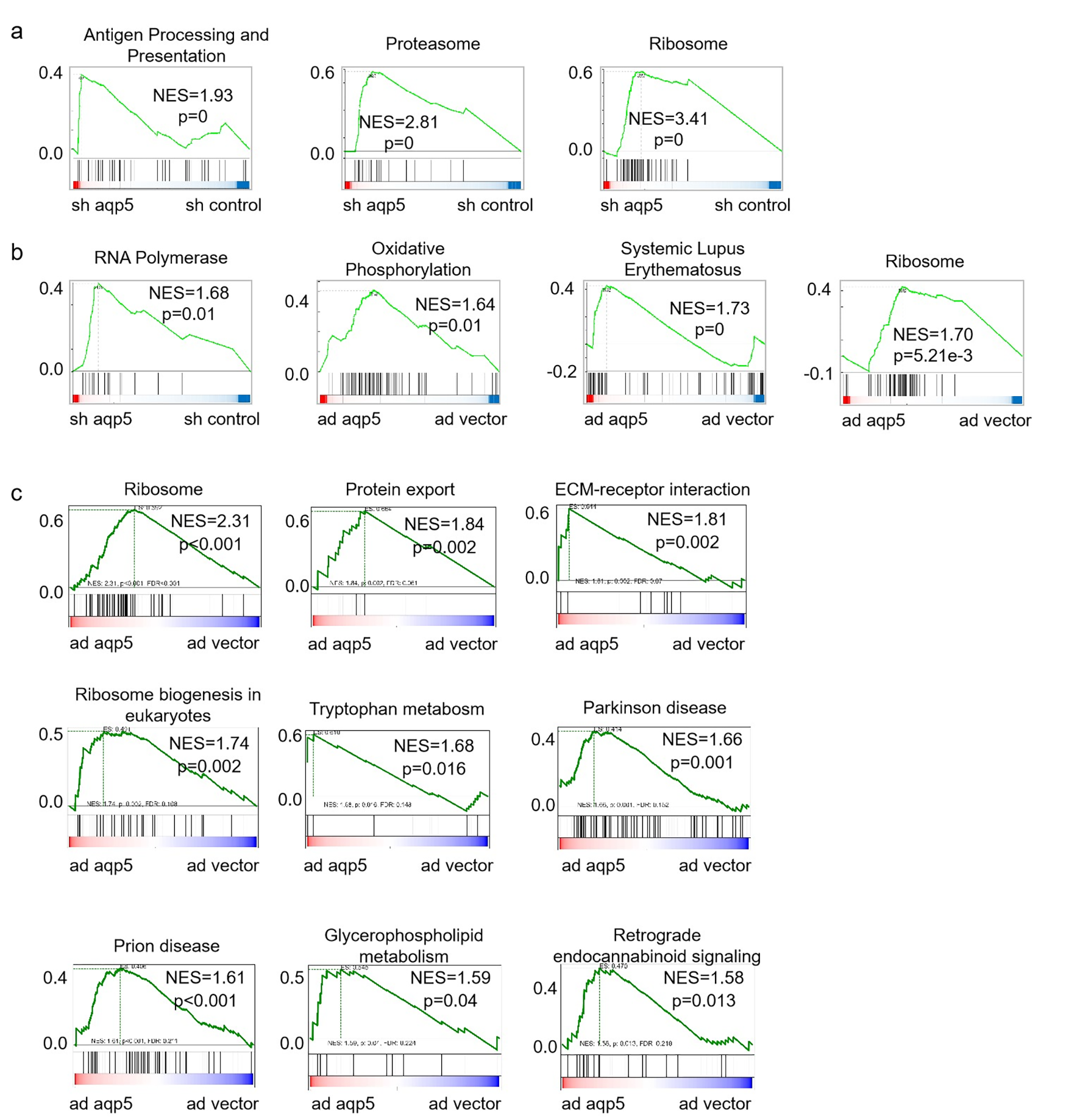

Supplement: Supplementary file 1 — Additional file 1: Figure S1. Expression of marker genes in gastric cancer tissue epithelial/stem cellsand cultured adherent/spheroid cells. Figure S2. AQP5 expression in AGS/HGC-27/GES-1 spheroids and adherent cells. Figure S3. AQP5 promotes gastric cancer development in vitro and in vivo. Figure S4. Expression of AQP5 in GC-CSCs. Figure S5. AQP5 promotes the stemness of GC-CSCs. Figure S6. Effect of AQP5 on LGR5 expression.Figure S7. Cellular pathways affected by AQP5. Figure S8. ATG7 is the key regulator of GC cell autophagy. Figure S9. AQP5 affects key autophagy proteins.Figure S10. AQP5 promotes malignant behaviors of GC-CSCs by regulatingK63-mediated ubiquitination of ULK1.Figure S11. Interaction of AQP5, TRIM21 and ULK1. Figure S12. AQP5 promotes self-renewal via TRIM21 in GC-CSCs. [file 13046_2022_2532_MOESM1_ESM.zip › 13046_2022_2532_MOESM1_ESM/figure S7.tif]

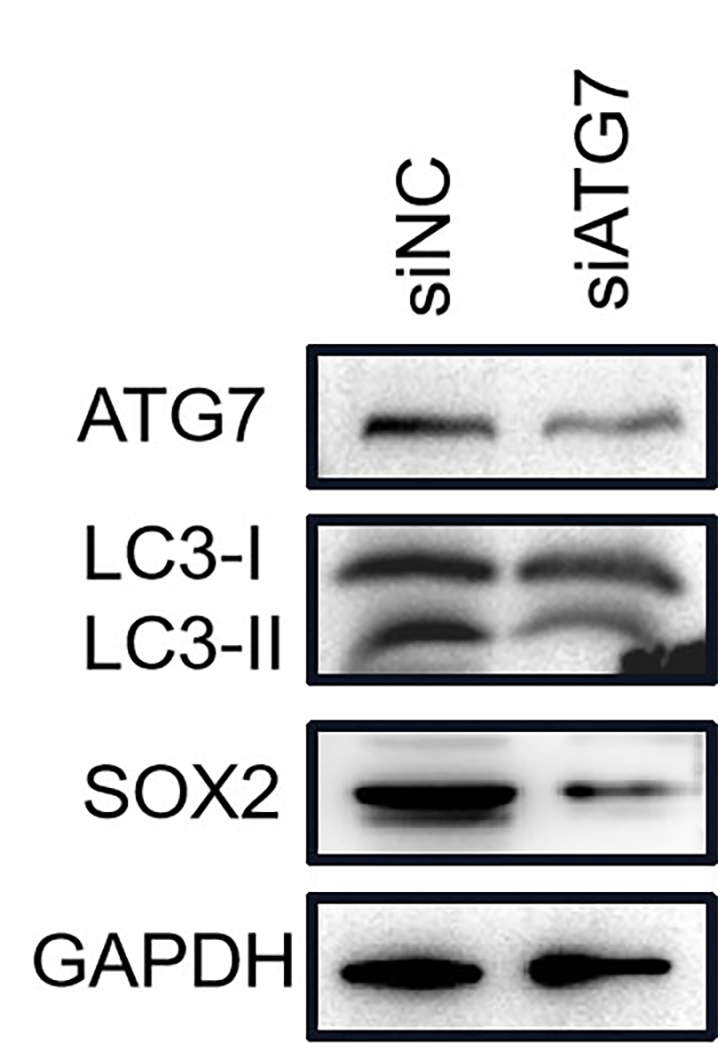

Supplement: Supplementary file 1 — Additional file 1: Figure S1. Expression of marker genes in gastric cancer tissue epithelial/stem cellsand cultured adherent/spheroid cells. Figure S2. AQP5 expression in AGS/HGC-27/GES-1 spheroids and adherent cells. Figure S3. AQP5 promotes gastric cancer development in vitro and in vivo. Figure S4. Expression of AQP5 in GC-CSCs. Figure S5. AQP5 promotes the stemness of GC-CSCs. Figure S6. Effect of AQP5 on LGR5 expression.Figure S7. Cellular pathways affected by AQP5. Figure S8. ATG7 is the key regulator of GC cell autophagy. Figure S9. AQP5 affects key autophagy proteins.Figure S10. AQP5 promotes malignant behaviors of GC-CSCs by regulatingK63-mediated ubiquitination of ULK1.Figure S11. Interaction of AQP5, TRIM21 and ULK1. Figure S12. AQP5 promotes self-renewal via TRIM21 in GC-CSCs. [file 13046_2022_2532_MOESM1_ESM.zip › 13046_2022_2532_MOESM1_ESM/figure S8.tif]

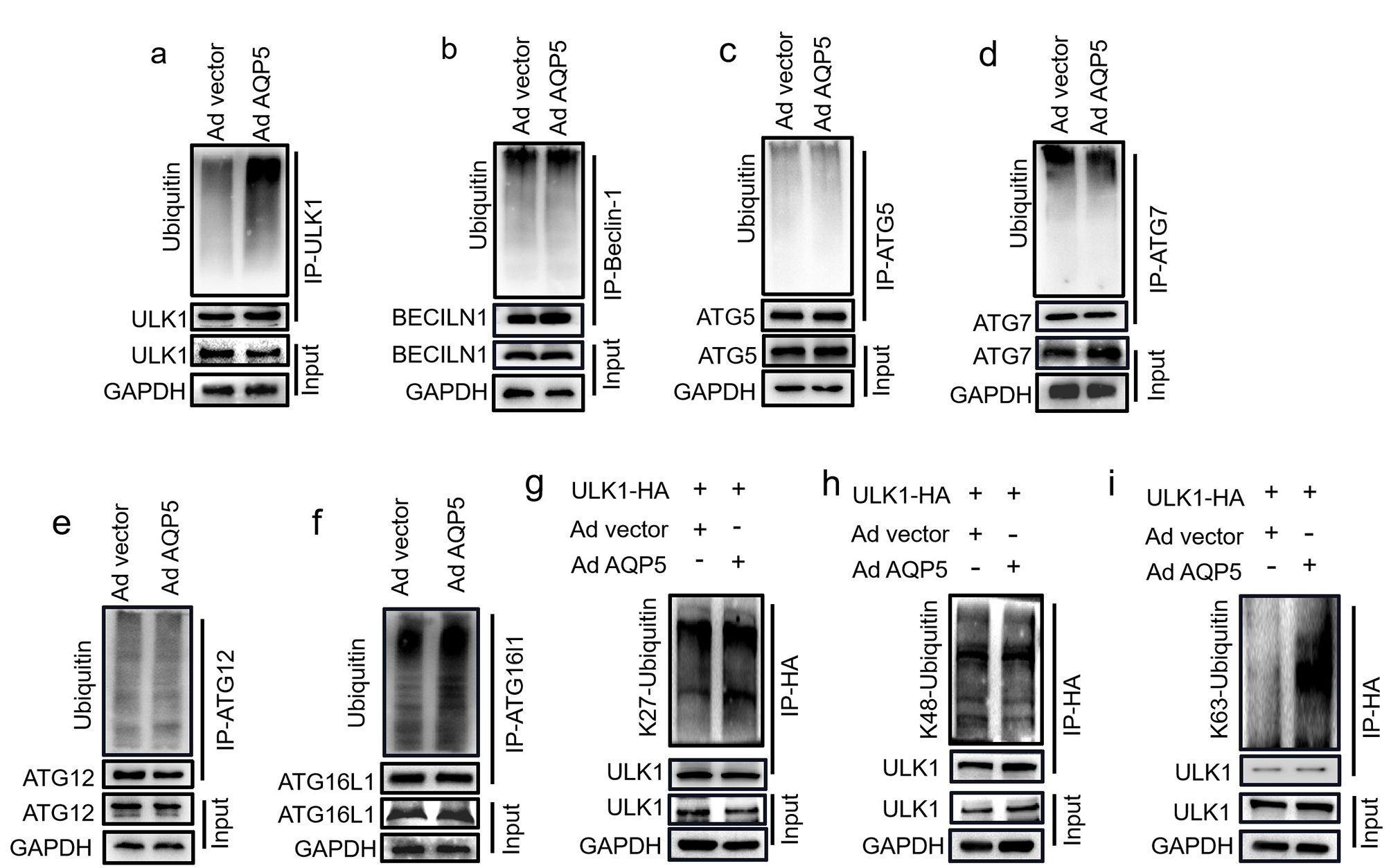

Supplement: Supplementary file 1 — Additional file 1: Figure S1. Expression of marker genes in gastric cancer tissue epithelial/stem cellsand cultured adherent/spheroid cells. Figure S2. AQP5 expression in AGS/HGC-27/GES-1 spheroids and adherent cells. Figure S3. AQP5 promotes gastric cancer development in vitro and in vivo. Figure S4. Expression of AQP5 in GC-CSCs. Figure S5. AQP5 promotes the stemness of GC-CSCs. Figure S6. Effect of AQP5 on LGR5 expression.Figure S7. Cellular pathways affected by AQP5. Figure S8. ATG7 is the key regulator of GC cell autophagy. Figure S9. AQP5 affects key autophagy proteins.Figure S10. AQP5 promotes malignant behaviors of GC-CSCs by regulatingK63-mediated ubiquitination of ULK1.Figure S11. Interaction of AQP5, TRIM21 and ULK1. Figure S12. AQP5 promotes self-renewal via TRIM21 in GC-CSCs. [file 13046_2022_2532_MOESM1_ESM.zip › 13046_2022_2532_MOESM1_ESM/figure S9.tif]
