# Supplementary material for: AQP5 complements LGR5 to determine the fates of gastric cancer stem cells through regulating ULK1 ubiquitination
Source: J Exp Clin Cancer Res. 2022 Nov 14;41:322. doi: 10.1186/s13046-022-02532-w (PMC9661769; doi:10.1186/s13046-022-02532-w)
Supplement: Supplementary file 2 — Additional file 2: Supplementary Table 1. Sequences ofreal-time PCR primers. Supplementary Table 2. Primary Antibodies. Supplementary Table 3. Second Antibodies. Supplementary Table 4. ShRNA or siRNA Oligonucleotides. Supplementary Table 5. Tumorigenicityof knockdown AQP5 and control group. Supplementary Table 6. Tumorigenicity of knockdown AQP5 and control group. Supplementary Table 7. Tumorigenicityof knockdown AQP5 and LGR5. Supplementary Table 8. Differentially expressed genes between exogenousoverexpression of AQP5 and control group. Supplementary Table 9. Differentially expressed genes between exogenousoverexpression of AQP5 and control group. Supplementary Table 10. Identification of the AQP5 protein complex by mass spectrometry. [file 13046_2022_2532_MOESM2_ESM.docx]

Supplementary Table 1. Sequences of real-time PCR primers

| mRNA | Primer | Sequences (5′-3 ′) | Annealing temperature |
| --- | --- | --- | --- |
| Human-*aqp5* | Forward | TCCATTGGCCTGTCTGTCAC | 60°C |
|  | Reverse | GTCCTCGTCAGGCTCATACG |  |
| Human- *lyz* | Forward | TCAGCCTAGCAAACTGGATGT | 56°C |
|  | Reverse | ACAAGCTACAGCATCAGCGA |  |
| Human- *reg1a* | Forward | ATGACCCCAAAAAGAACCGC | 58°C |
|  | Reverse | AGTTGGAGAGATGGTCCGGT |  |
| Human- *reg3a* | Forward | CCATATCCCACCAGAGAGTGAC | 57°C |
|  | Reverse | GCCAGATCTGCATCTGTCCA |  |
| Human-*ctgf* | Forward | ATTCTGTGGAGTATGTACCGAC | 54°C |
|  | Reverse | GTCTCCGTACATCTTCCTGTAG |  |
| Human-*cd133* | Forward | GAGCTAAGGGAAGGGCGG | 60°C |
|  | Reverse | TTCTGTCTGAGGCTGGCTTG |  |
| Human-*nanog* | Forward | CCTGATTCTTCCACCAGTCC | 60°C |
|  | Reverse | TGCTATTCTTCGGCCAGTTG |  |
| Human-*lgr5* | Forward | CATCAGCTATGTGCCCCCAA | 62°C |
|  | Reverse | TGTGGAGCCCATCAAAGCAT |  |
| Human-*β-actin* | Forward | GACCTGTACGCCAACACAGT | 59°C |
|  | Reverse | CTCAGGAGGAGCAATGATCT |  |
| Human-*ck18* | Forward | CTGTCCTTTCTCTCTCCCCG | 60°C |
|  | Reverse | CGTTCAGGCTTTGCATGGTC |  |
| Human-*oct18* | Forward | TTGAGGCTCTGCAGCTTAG | 60°C |
|  | Reverse | GCCGGTTACAGAACCACAC |  |
| Human-*muc1* | Forward | TCTCAGACGTCAGCGTGAGT | 60°C |
|  | Reverse | CTCGCTCATAGGATGGTAGG |  |
| Human-*sox2* | Forward | ACACCAATCCCATCCACACT | 60°C |
|  | Reverse | GCAAACTTCCTGCAAAGCTC |  |

Supplementary Table 2. Primary Antibodies

| Primary Antibody | Source | Detection and dilution ratio | Manufacturer |
| --- | --- | --- | --- |
| GAPDH | Rabbit | WB1:10000 | Bioworld(RRID:AB_2651132) |
| β-Actin | Rabbit | WB1:1000 | Abclonal（RRID:AB_2737399） |
| AQP5 | Rabbit | WB1:1000 | Abways |
| AQP5 | Rabbit | FC 1:200 | Absin |
| LGR5 | Rabbit | WB1:1000/IHC1:100 | Abclonal（RRID:AB_2759834） |
| LC3 | Rabbit | WB1:1000 | Cell signaling technology |
| P62 | Mice | WB1:1000 | Cell signaling technology（RRID:AB_2890160） |
| CD133 | Rabbit | WB1:1000 | Abclonal |
| Sox2 | Mice | WB1:1000/IHC1:100 | Cell signaling technology（RRID:AB_2195767） |
| ULK1 | Rabbit | WB1:500 | Abways |
| BECLIN1 | Rabbit | IHC1:50 | Bioworld |
| HA-tag | Rabbit | WB1:1000 | Cell signaling technology（RRID:AB_2890916） |
| FLAG-tag | Rabbit | WB1:1000 | Cell signaling technology |
| MYC-tag | Goat | WB1:1000 | Cell signaling technology（RRID:AB_10692100） |
| Ubiquitin | Mice | WB1:1000 | Cell signaling technology（RRID:AB_331292） |
| K63-Ubiquitin | Mice | WB1:1000 | Cell signaling technology |
| ATG5 | Rabbit | WB1:1000 | Cell signaling technology (RRID:AB_2630393) |
| PE anti-human CD133 Antibody | Rabbit | Flow 1:200 | Biolegend(RRID:AB_2734477) |
| PE anti-human LGR5 Antibody | Rabbit | FC 1:200 | Biolegend(RRID:AB_2922590) |
| PE anti-human CD44 Antibody | Rabbit | FC 1:200 | Biolegend(RRID:AB_2260222) |
| PE Mouse IgG1, kappa Isotype | Rabbit | FC 1:200 | Biolegend(RRID:AB_326435) |
| ATG7 | Rabbit | WB1:1000 | Cell signaling technology (RRID:AB_2227783) |
| ATG12 | Rabbit | WB1:1000 | Cell signaling technology (RRID:AB_2059086) |
| ATG16L1 | Rabbit | WB1:1000 | Cell signaling technology (RRID:AB_10950320) |
| K48-Ubiquitin | Rabbit | WB1:1000 | Abclonal |
| K27-Ubiquitin | Rabbit | WB1:1000 | Abclonal |
| TRIM21 | Sheep | WB1:400 | R&d Systems |
| PE Mouse IgG1, kappa Isotype | Rabbit | Flow 1:200 | Biolegend(RRID:AB_326435) |

Supplementary Table 3. Second Antibodies

| Second Antibody | Detection and dilution ratio | Manufacturer |
| --- | --- | --- |
| Anti-Rabbit antibody-HRP | WB1:2000 | Cell signaling technology |
| Anti-Mice antibody-HRP | WB1:2000 | Cell signaling technology |
| Anti-Sheep antibody-HRP | WB1:2000 | Abcam(RRID:AB_955383) |
| Anti-Goat antibody-HRP | WB1:2000 | Cell signaling technology |
| Anti-sheep antibody-HRP | WB1:2000 | Abcam(RRID:AB_955383) |
| Anti-Mice antibody-Alexa Fluor 488 | IF 1:1000 | Cell signaling technology |
| Anti-Goat antibody-Alexa Fluor 555 | IF 1:1000 | Abcam |
| Anti-Rabbit antibody-Alexa Fluor 488 | FC 1:200 | Absin |

Supplementary Table 4. ShRNA or siRNA Oligonucleotides

| Target Gene | Sequences (5′-3 ′) |
| --- | --- |
| Human-AQP5 | Forward CcggCCGTGTTCGCAGAGTTCTTcTCAAGAGAAAGAACTCTGCGAACACGGTTTTTTg |
|  | Reverse aattcaaaaaaCCGTGTTCGCAGAGTTCTTTCTCTTGAgAAGAACTCTGCGAACACGG |
| Human-ATG7(siRNA) | Forward CCAACACACUCGAGUCUUUdUdUdTdT |
|  | Reverse AAAGACUCGAGUGUGUUGGdUdUdTdT |
| Human-TRIM21(siRNA) | Forward GCAGCACGCUUGACAAUGAdTdT |
|  | Reverse UCAUUGUCAAGCGUGCUGCdTdT |
| Negative control(siRNA) | Forward UUCUCCGAACGUGUCACGUdTdT |
|  | Reverse ACGUGACACGUUCGGAGAAdTdT |
| Human-UBB(siRNA) | Forward GUGAAGGCCAAGAUCCAAGAUdTdT |
|  | Reverse AUCUUGGAUCUUGGCCUUCACdTdT |
| Human-UBC(siRNA) | Forward CGAGAAUGUCAAGGCAAAGAUdTdT |
|  | Reverse AUCUUUGCCUUGACAUUCUCGdTdT |
| Human-LGR5 | Forward  CcggTGCGGGAAACGCTCTGACATCTCGAGTATGTCAGAGCGTTTCCCGCATTTTTg |
|  | Reverse  GATCCAAAAATGCGGGAAACGCTCTGACATA CTCGAGTATGTCAGAGCGTTTCCCGCA |

Supplementary Table 5. Tumorigenicity of knockdown AQP5 and control group

|  | 10^2^  (cells) | 10^3^  (cells) | 10^4^  (cells) | 10^5^  (cells) | 10^6^  (cells) |
| --- | --- | --- | --- | --- | --- |
| sh control | n=3/6 | n=5/6 | n=5/6 | n=5/6 | n=6/6 |
| sh AQP5 | n=0/6 | n=3/6 | n=5/6 | n=5/6 | n=6/6 |

Supplementary Table 6. Tumorigenicity of knockdown AQP5 and control group

|  | 10^2^  (cells) | 10^3^  (cells) | 10^4^  (cells) | 10^5^  (cells) | 10^6^  (cells) |
| --- | --- | --- | --- | --- | --- |
| Ad vector | n=1/6 | n=5/6 | n=5/6 | n=5/6 | n=6/6 |
| Ad AQP5 | n=3/6 | n=6/6 | n=5/6 | n=6/6 | n=6/6 |

Supplementary Table 7. Tumorigenicity of knockdown AQP5 and LGR5

|  | 10^2^  (cells) | 10^3^  (cells) | 10^4^  (cells) | 10^5^  (cells) |
| --- | --- | --- | --- | --- |
| sh control | n=1/4 | n=4/4 | n=4/4 | n=4/4 |
| sh LGR5 | n=0/4 | n=2/4 | n=4/4 | n=4/4 |
| sh AQP5 | n=0/4 | n=3/4 | n=4/4 | n=4/4 |
| sh AQP5/LGR5 | n=0/4 | n=0/4 | n=2/4 | n=4/4 |

Supplementary Table 8. Differentially expressed genes between exogenous overexpression of AQP5 and control group

| Gene Symbol | log2 (Ad aqp5 / Ad vector) | FDR (Ad aqp5 / Ad vector) |
| --- | --- | --- |
| 'AQP5' | 10.62976296 | 0 |
| 'LYZ' | 1.164697279 | 0 |
| 'PCDH7' | 1.575058377 | 1.29E-195 |
| 'FGG' | 2.029559798 | 1.64E-171 |
| 'DYNLRB1' | 1.197199974 | 2.31E-163 |
| 'FGB' | 2.130123993 | 1.15E-129 |
| 'SLC7A8' | 1.061580843 | 8.72E-119 |
| 'LOC112267876' | 1.339806666 | 2.60E-103 |
| 'PRSS2' | 1.013539009 | 3.48E-102 |
| 'CLDN2' | 1.121128204 | 5.83E-91 |
| 'FGA' | 1.265242166 | 1.20E-87 |
| 'ALPP' | -1.357756174 | 6.00E-87 |
| 'IGFBP5' | 1.704290901 | 5.29E-82 |
| 'FLG' | -1.211504105 | 2.75E-68 |
| 'HOTS' | 1.368820442 | 1.77E-63 |
| 'FRAS1' | -1.018513298 | 7.52E-61 |
| 'TENM3' | -1.256098359 | 1.44E-60 |
| 'RPL17-C18orf32' | 5.977279923 | 1.64E-48 |
| 'CHAC1' | -1.119874832 | 1.69E-47 |
| 'RHOBTB1' | 2.552296312 | 2.54E-45 |
| 'KIAA0754' | -1.529024329 | 5.97E-40 |
| 'GNE' | -1.475652782 | 4.99E-36 |
| 'REG1A' | 1.153083359 | 7.65E-36 |
| 'TMEM189-UBE2V1' | -7.807354922 | 3.62E-35 |
| 'PTCD1' | -1.445411148 | 1.48E-34 |
| 'LOC102724200' | 7.294620749 | 6.99E-27 |
| 'PMCH' | 1.573978194 | 7.30E-26 |
| 'ANK1' | -1.350497247 | 5.43E-23 |
| 'PLAT' | -1.417767292 | 2.35E-20 |
| 'SCEL' | -1.00507099 | 1.72E-19 |
| 'POC1B-GALNT4' | 1.738996129 | 9.42E-19 |
| 'CDRT4' | -7.118941073 | 1.47E-18 |
| 'GPX5' | 1.563003248 | 7.35E-18 |
| 'HR' | 1.077637411 | 1.00E-17 |
| 'SARM1' | -1.26934818 | 4.19E-17 |
| 'MATN2' | -1.988318195 | 7.27E-17 |
| 'FAM72C' | -1.358971437 | 1.19E-16 |
| 'PGC' | 2.569263814 | 3.24E-16 |
| 'GPR75-ASB3' | -2.561655127 | 3.80E-16 |
| 'PRDM1' | -1.078777113 | 6.72E-16 |
| 'RND1' | 1.076272741 | 1.59E-15 |
| 'COL17A1' | -1.650925763 | 2.02E-15 |
| 'CCDC80' | -1.527247003 | 2.40E-15 |
| 'VGLL1' | -1.625461863 | 3.09E-15 |
| 'C7orf55-LUC7L2' | 1.133050758 | 3.13E-15 |
| 'PRAF2' | 1.142132849 | 1.63E-14 |
| 'CORO7-PAM16' | -6.209453366 | 2.22E-14 |
| 'GALNT4' | -1.321928095 | 2.57E-13 |
| 'IHH' | 1.319501357 | 2.73E-13 |
| 'LGALS9' | 1.479093866 | 5.42E-13 |
| 'LRRC37A' | -1.222392421 | 5.88E-13 |
| 'MMP7' | 1.961005868 | 2.41E-12 |
| 'HABP2' | 2.216811389 | 2.62E-12 |
| 'HSPE1-MOB4' | -5.672425342 | 1.05E-11 |
| 'MPP1' | 2.425484287 | 1.40E-11 |
| 'TTN' | -2.700439718 | 1.74E-11 |
| 'NFE2' | 1.082989365 | 3.82E-11 |
| 'TNFAIP8L2-SCNM1' | -6.781359714 | 4.11E-11 |
| 'CAPN8' | -1.153570389 | 4.89E-11 |
| 'LOC101928589' | 2.286881148 | 6.45E-11 |
| 'TBC1D3C' | 2.599037686 | 9.62E-11 |
| 'RPS10-NUDT3' | -6.303780748 | 1.58E-10 |
| 'TCN1' | 1.721760723 | 3.21E-10 |
| 'ABHD14A-ACY1' | -6.658211483 | 6.09E-10 |
| 'TIAF1' | 3.724892762 | 1.34E-09 |
| 'IFI44L' | 1.35614381 | 2.66E-09 |
| 'FGF20' | 1.235783041 | 6.03E-09 |
| 'EAF2' | 1.036592053 | 6.31E-09 |
| 'C18orf32' | -1.040434864 | 1.02E-08 |
| 'C8orf44-SGK3' | -2.067114196 | 1.91E-08 |
| 'NPY4R' | 1.868755467 | 1.66E-07 |
| 'LDLRAD2' | 1.064705689 | 1.90E-07 |
| 'BUB1B-PAK6' | 5.169925001 | 3.04E-07 |
| 'PXDN' | -2.662965013 | 3.19E-07 |
| 'SPDEF' | 1.281850656 | 3.61E-07 |
| 'TVP23C-CDRT4' | 1.00877021 | 3.69E-07 |
| 'RGS8' | -1.750021747 | 4.06E-07 |
| 'HIST1H4K' | 8.693486957 | 5.74E-07 |
| 'NPY4R2' | -2.222392421 | 8.14E-07 |
| 'PTPRO' | 2.021061616 | 9.88E-07 |
| 'ABCA12' | -1.444784843 | 1.22E-06 |
| 'KRT6A' | -2.247927513 | 1.67E-06 |
| 'SHC3' | -1.08246216 | 1.68E-06 |
| 'TOX3' | 2.263034406 | 1.96E-06 |
| 'C6orf222' | 1.01720929 | 1.96E-06 |
| 'TUBB6' | -2.502500341 | 2.92E-06 |
| 'AGAP5' | 1.944290567 | 3.66E-06 |
| 'NT5E' | -3.201633861 | 4.01E-06 |
| 'CFB' | 1.062284278 | 4.66E-06 |
| 'ADAMTSL2' | 1.092118202 | 5.34E-06 |
| 'RASA4B' | -1.309684499 | 5.89E-06 |
| 'HIST1H4J' | -8.409390936 | 7.09E-06 |
| 'LOC101059949' | -2.23878686 | 1.90E-05 |
| 'TBC1D3H' | -1.371968777 | 2.04E-05 |
| 'ACVR1C' | 1.624490865 | 2.24E-05 |
| 'LOC644634' | 4.906890596 | 2.49E-05 |
| 'DUOX2' | -1.74723393 | 2.59E-05 |
| 'S100A9' | -1.984503664 | 2.70E-05 |
| 'ALPG' | -1.302028537 | 3.93E-05 |
| 'TGIF2-RAB5IF' | -6.459431619 | 5.09E-05 |
| '112267923' | -5.129283017 | 5.09E-05 |
| 'SPINK1' | 1.226068079 | 6.91E-05 |
| 'MCAM' | -1.716207034 | 7.58E-05 |
| 'LOC102724951' | -2.371968777 | 7.98E-05 |
| 'LOC107987477' | 5.087462841 | 8.72E-05 |
| 'DGCR6' | 6.339850003 | 8.72E-05 |
| 'TTLL3' | -1.519374159 | 8.81E-05 |
| 'SCHIP1' | -5.832890014 | 9.79E-05 |
| 'ANKRD20A4' | 2.963474124 | 1.04E-04 |
| 'KCNC3' | -1.174774401 | 1.18E-04 |
| 'PAK6' | -2.201633861 | 1.33E-04 |
| 'GIF' | 5.832890014 | 1.61E-04 |
| 'LOC388436' | 5.491853096 | 1.61E-04 |
| 'CEMP1' | 4.044394119 | 1.61E-04 |
| 'FADS1' | -1.719892081 | 1.73E-04 |
| 'NPPB' | -1.160077215 | 2.30E-04 |
| 'SERPINE1' | -1.267933205 | 2.55E-04 |
| 'MRPL23' | -1.79970135 | 2.58E-04 |
| 'TICAM2' | 4.700439718 | 2.97E-04 |
| 'ELF5' | -3.321928095 | 3.57E-04 |
| 'SNRPN' | -1.650253961 | 4.52E-04 |
| 'LOC101928841' | -1.237039197 | 5.00E-04 |
| 'ITPR1' | -1.432959407 | 5.42E-04 |
| 'CDKN1C' | 1.04405518 | 6.30E-04 |
| 'FAM43A' | -4.523561956 | 6.76E-04 |
| 'PPARGC1A' | 1.06871275 | 7.57E-04 |
| 'IGFBP7' | -4 | 7.65E-04 |
| 'IL1B' | -3.906890596 | 7.65E-04 |
| 'SBSPON' | -2.039528364 | 7.67E-04 |
| 'ALOXE3' | -1.678071905 | 8.47E-04 |
| 'PIK3IP1' | 1.025237291 | 8.77E-04 |

Supplementary Table 9. Differentially expressed genes between exogenous overexpression of AQP5 and control group

| Gene Symbol | log2 (sh aqp5 / shcontrol) | FDR (sh aqp5 / shcontrol) |
| --- | --- | --- |
| 'SIK1B' | -1.382899576 | 0 |
| 'MYL9' | 1.147537983 | 0 |
| 'HSPH1' | 1.052850241 | 0 |
| 'ADRM1' | 1.172384025 | 0 |
| 'SYNPO' | -2.881617732 | 0 |
| 'CLTB' | 1.040449311 | 0 |
| 'S100A16' | 1.048817735 | 0 |
| 'DAG1' | -1.14165405 | 0 |
| 'EGR1' | -4.569830255 | 0 |
| 'SERPINB1' | -1.294229832 | 0 |
| 'FASN' | -1.16380944 | 0 |
| 'DKK1' | 1.018729739 | 0 |
| 'FOXC1' | -1.264280436 | 0 |
| 'ATP11A' | -1.235527471 | 0 |
| 'SLC39A14' | -1.236122238 | 0 |
| 'CABIN1' | -1.39475922 | 0 |
| 'SRRM2' | -1.02034772 | 0 |
| 'FOS' | -6.046057169 | 0 |
| 'FOSB' | -5.395311284 | 0 |
| 'GATM' | -1.685320465 | 0 |
| 'HSPB8' | 1.549308746 | 0 |
| 'SFN' | 1.537998017 | 0 |
| 'GPX3' | 1.618858997 | 0 |
| 'HMOX1' | 1.358516218 | 0 |
| 'NR4A1' | -2.242120003 | 0 |
| 'HES1' | -2.456110814 | 0 |
| 'HSPA1B' | 2.659301023 | 0 |
| 'HSPA8' | 1.479678936 | 0 |
| 'DNAJB1' | 1.350964399 | 0 |
| 'HSPG2' | -1.387248359 | 0 |
| 'KRT6A' | 5.685271342 | 0 |
| 'LAMA5' | -1.228502515 | 0 |
| 'BCAM' | -1.334330321 | 0 |
| 'MYL6' | 1.011278924 | 0 |
| 'SERPINB2' | 5.34021846 | 0 |
| 'JPT1' | 1.125797457 | 0 |
| 'PSMB5' | 1.062072689 | 0 |
| 'RRBP1' | -1.337888401 | 0 |
| 'SCD' | -1.899289791 | 0 |
| 'C19orf33' | 1.265592545 | 0 |
| 'SLC12A2' | -1.044380597 | 0 |
| 'BMPR2' | -1.627787689 | 0 |
| 'SOX9' | -1.438556271 | 0 |
| 'TAGLN' | 1.918843398 | 0 |
| 'ZFP36' | -1.424800328 | 0 |
| 'MYH14' | -1.117603966 | 0 |
| 'ZNF703' | -1.53837801 | 0 |
| 'AXIN2' | -1.289167258 | 0 |
| 'ENC1' | -1.071993337 | 0 |
| 'LGR5' | -1.313971606 | 0 |
| 'SQSTM1' | 1.267824271 | 0 |
| 'IL32' | 1.938668157 | 0 |
| 'FOXQ1' | -1.115506787 | 0 |
| 'CTGF' | -1.369372152 | 6.46E-301 |
| 'CHD3' | -1.5742143 | 5.49E-295 |
| 'TXNRD1' | 1.253167376 | 1.28E-291 |
| 'IDH1' | -1.369885337 | 7.34E-291 |
| 'PLK2' | -1.366739721 | 1.65E-287 |
| 'NAV2' | -2.829224102 | 2.81E-286 |
| 'NID1' | -3.363935621 | 6.99E-285 |
| 'SRSF5' | -1.302461655 | 2.06E-283 |
| 'IRF2BP2' | -1.284588287 | 1.32E-275 |
| 'ATP1B1' | -1.162024798 | 3.92E-274 |
| 'MYEOV' | 1.086615642 | 5.96E-270 |
| 'SDCBP2' | 2.27806221 | 3.49E-269 |
| 'TNFRSF12A' | 1.01583151 | 1.23E-267 |
| 'HSPA1A' | 2.570400207 | 1.67E-266 |
| 'SRSF6' | -1.12259915 | 2.02E-265 |
| 'ATP9A' | -1.229396809 | 8.19E-260 |
| 'LRP1' | -2.035792262 | 9.59E-260 |
| 'C1GALT1' | -1.338849035 | 2.10E-253 |
| 'MUC1' | -1.552187078 | 2.17E-249 |
| 'RDH11' | -1.819303325 | 1.04E-241 |
| 'SPRR2D' | 6.367025551 | 9.87E-241 |
| 'PGM1' | -1.322585584 | 5.91E-233 |
| 'SLC7A8' | -2.838309455 | 7.68E-232 |
| 'BHLHE40' | -1.231149135 | 5.43E-230 |
| 'HSPA6' | 7.056057037 | 1.16E-229 |
| 'DEDD2' | 1.606722477 | 1.18E-226 |
| 'GPX2' | 1.249383738 | 7.37E-225 |
| 'MICAL2' | -1.132376215 | 3.35E-224 |
| 'TM7SF3' | -1.121470407 | 9.22E-222 |
| 'CLU' | 2.249181422 | 1.61E-221 |
| 'TM4SF1' | 1.383185763 | 2.78E-216 |
| 'RUNX1' | -1.291320403 | 1.68E-214 |
| 'IER2' | -1.198115265 | 1.78E-209 |
| 'H2AFX' | 1.27735743 | 2.40E-208 |
| 'C4BPB' | 1.657916433 | 2.71E-207 |
| 'DYNLRB1' | 1.306139209 | 3.98E-207 |
| 'FOSL1' | 1.244293579 | 1.50E-196 |
| 'SCARB2' | -1.13629143 | 5.87E-194 |
| 'CLDN2' | -3.584962501 | 6.27E-194 |
| 'TNRC18' | -1.308394939 | 1.09E-193 |
| 'RNF213' | -1.510000443 | 9.80E-193 |
| 'SIPA1L3' | -1.020288923 | 7.73E-191 |
| 'SREBF1' | -1.177801427 | 5.05E-190 |
| 'CTSH' | -1.046559401 | 1.40E-188 |
| 'LENG8' | -1.210114092 | 2.29E-185 |
| 'FPR3' | -1.265149485 | 6.98E-183 |
| 'UNC5CL' | -1.447626918 | 3.48E-182 |
| 'TRIM29' | 3.137740245 | 1.21E-175 |
| 'EMP3' | 1.151859094 | 1.46E-173 |
| 'ATF3' | -1.393446381 | 1.54E-171 |
| 'ZFP36L2' | -1.129382179 | 3.83E-170 |
| 'NUMA1' | -1.136002615 | 1.67E-168 |
| 'FOXP1' | -1.491156632 | 1.15E-163 |
| 'FGA' | -4.62388149 | 1.47E-161 |
| 'REC8' | -1.027844102 | 5.54E-159 |
| 'SERINC5' | -1.390244168 | 8.78E-159 |
| 'JUNB' | -1.311567268 | 1.42E-157 |
| 'ETS2' | -1.096437874 | 9.00E-157 |
| 'GCLM' | 1.364585992 | 7.92E-155 |
| 'LGR4' | -1.087560721 | 8.69E-154 |
| 'PTPRU' | -1.210640281 | 1.00E-149 |
| 'VEGFA' | -1.492935624 | 4.81E-149 |
| 'TMEM63A' | -1.033440967 | 3.23E-148 |
| 'VTN' | -1.190300009 | 1.37E-144 |
| 'SCNN1A' | -3.75227865 | 4.44E-143 |
| 'FUT1' | -1.50286374 | 1.98E-142 |
| 'EDAR' | -1.501334889 | 5.60E-141 |
| 'MOV10' | -1.011758884 | 1.95E-140 |
| 'ARID5B' | -1.474706325 | 4.33E-139 |
| 'GNPAT' | -1.163853593 | 1.03E-138 |
| 'CLSTN3' | -1.404479308 | 1.61E-137 |
| 'PRSS3' | 1.65422085 | 1.65E-137 |
| 'ID2' | -2.225382464 | 5.81E-137 |
| 'TRIM47' | 1.579233325 | 6.09E-136 |
| 'SYNE2' | -1.49990215 | 1.20E-132 |
| 'ROR1' | -1.582268392 | 4.28E-131 |
| 'KRT17' | 1.193131516 | 4.68E-128 |
| 'GJB3' | 1.149178264 | 6.53E-127 |
| 'ZC3H4' | -1.256877546 | 7.19E-127 |
| 'HMGCR' | -1.112084635 | 5.62E-126 |
| 'OSGIN1' | 1.944025481 | 6.47E-126 |
| 'CDH17' | -1.386870481 | 2.25E-125 |
| 'NIPSNAP1' | -1.604777236 | 8.81E-123 |
| 'PHC1' | -2.048744334 | 1.80E-122 |
| 'PHLDB2' | 1.54616023 | 5.05E-122 |
| 'CEACAM1' | -1.188987211 | 5.29E-122 |
| 'GDE1' | -1.476671824 | 5.35E-121 |
| 'ZMYM3' | -1.310651476 | 1.27E-120 |
| 'MORC4' | -1.202635791 | 9.56E-120 |
| 'C1orf198' | -1.324404825 | 2.14E-119 |
| 'SORL1' | -1.521988584 | 7.04E-119 |
| 'APBB2' | -1.332374225 | 4.22E-118 |
| 'GNS' | -1.115477217 | 2.25E-116 |
| 'NPPB' | 3.042996062 | 2.52E-115 |
| 'PSG11' | -2.273300326 | 3.68E-115 |
| 'TGM2' | -2.04000235 | 9.71E-115 |
| 'FZD7' | -1.347972703 | 1.39E-114 |
| 'TBC1D2' | 1.678071905 | 7.08E-114 |
| 'LIPG' | -1.075568823 | 1.92E-113 |
| 'CDX2' | -1.118914972 | 7.28E-113 |
| 'TM7SF2' | -1.381001175 | 9.46E-111 |
| 'CFTR' | -1.729074809 | 4.14E-109 |
| 'GALNT5' | 1.805756368 | 1.92E-107 |
| 'ID1' | -1.071095686 | 2.42E-107 |
| 'LOC107986554' | -1.206242681 | 5.65E-107 |
| 'ANXA1' | 2.31753484 | 1.17E-106 |
| 'KIAA1217' | -1.370076931 | 1.49E-106 |
| 'TJP3' | -1.718561014 | 4.13E-105 |
| 'FAM171A1' | -1.269060481 | 1.70E-103 |
| 'PEAK1' | -1.079163518 | 1.37E-100 |
| 'TP53INP1' | -1.827392675 | 4.04E-100 |
| 'ZMIZ1' | -1.316837223 | 3.01E-99 |
| 'F5' | -1.899473124 | 4.37E-99 |
| 'TMEM265' | 1.518854953 | 2.34E-98 |
| 'ADD3' | -1.001348523 | 5.02E-98 |
| 'APOBEC3B' | 1.014304117 | 9.97E-98 |
| 'KLHL21' | 1.449884185 | 1.36E-97 |
| 'TMC5' | -1.763865098 | 2.29E-96 |
| 'SP5' | -1.002466849 | 2.60E-96 |
| 'ZNF687' | -1.153474481 | 9.66E-96 |
| 'HSD17B10' | 1.109071699 | 1.99E-95 |
| 'COL27A1' | -1.934046927 | 2.02E-95 |
| 'KDM4B' | -1.202517054 | 2.62E-95 |
| 'SEMA3B' | -1.206962381 | 7.25E-94 |
| 'ARHGAP18' | -1.03234786 | 2.71E-93 |
| 'PBX1' | -2.024585638 | 1.51E-90 |
| 'ARRB1' | -1.061280155 | 1.75E-90 |
| 'PSAT1' | -1.042463112 | 2.26E-89 |
| 'ALCAM' | -1.138599484 | 4.44E-89 |
| 'H6PD' | -1.537724137 | 3.71E-88 |
| 'HID1' | -1.433230616 | 1.08E-87 |
| 'RALGAPA2' | -1.067648692 | 1.54E-87 |
| 'SLC9A7' | -1.465223256 | 1.61E-87 |
| 'NFIX' | -1.141146495 | 1.62E-87 |
| 'VPS13C' | -1.239223445 | 7.57E-87 |
| 'CACHD1' | -2.233059346 | 7.61E-87 |
| 'PCSK9' | -1.632915724 | 1.80E-86 |
| 'MYO18A' | -1.421359787 | 2.25E-86 |
| 'ZNF532' | -1.710220739 | 1.15E-85 |
| 'SIPA1L1' | -1.13843011 | 3.89E-85 |
| 'EPHA4' | -1.296734563 | 1.55E-83 |
| 'IGFBP4' | -1.01278944 | 3.40E-82 |
| 'KLF2' | -1.38600616 | 7.25E-82 |
| 'HERPUD1' | 1.197826305 | 3.85E-80 |
| 'PIK3C2B' | -1.036321514 | 4.22E-80 |
| 'IGFBP5' | -5.209453366 | 7.10E-79 |
| 'SGK1' | -1.234899735 | 1.10E-78 |
| 'GLA' | 1.034853587 | 1.52E-78 |
| 'GPT2' | -1.020148219 | 2.80E-78 |
| 'PRKACA' | -1 | 1.68E-77 |
| 'SOX12' | -1.270350953 | 2.05E-77 |
| 'MSRB1' | 1.036846724 | 7.72E-77 |
| 'KRT81' | 2.323634417 | 2.79E-76 |
| 'ECM1' | 1.495197767 | 3.31E-76 |
| 'TRMT61A' | 1.078002512 | 7.20E-75 |
| 'PAQR8' | -2.482534688 | 7.75E-74 |
| 'MAP3K1' | -1.136235778 | 2.17E-72 |
| 'RGL3' | -1.858437328 | 6.14E-72 |
| 'FUT8' | -1.086661171 | 5.57E-71 |
| 'S100A2' | 1.827485877 | 8.64E-71 |
| 'GLUL' | -1.250517963 | 2.35E-70 |
| 'NHS' | -1.982033453 | 7.47E-70 |
| 'MT2A' | 1.271900629 | 1.14E-69 |
| 'SMIM24' | -1.204306078 | 1.74E-69 |
| 'SSBP3' | -1.130094558 | 1.95E-69 |
| 'RALGPS1' | -1.087036324 | 2.18E-69 |
| 'DTNB' | -1.249887507 | 3.80E-69 |
| 'SNTB1' | -1.135191369 | 4.33E-69 |
| 'IGFBP6' | 1.054320722 | 7.15E-69 |
| 'KLHL12' | -1.401921999 | 3.50E-67 |
| 'CELSR2' | -1.28764696 | 3.93E-67 |
| 'HAPLN3' | 1.247181852 | 1.10E-66 |
| 'CHAF1A' | -1.187106161 | 1.51E-66 |
| 'SERTAD1' | 1.02530115 | 2.10E-66 |
| 'TXNIP' | -1.507231197 | 5.96E-66 |
| 'ZFAND2A' | 2.541709303 | 6.01E-66 |
| 'STAT2' | -1.225113633 | 9.63E-66 |
| 'TCF7L2' | -1.182530119 | 1.23E-65 |
| 'CPTP' | 1.19523009 | 1.51E-65 |
| 'RELB' | 1.656877683 | 2.27E-65 |
| 'DUSP1' | -1.071019463 | 1.51E-64 |
| 'ABCA7' | -1.090889141 | 2.07E-64 |
| 'PELI2' | -1.948217458 | 3.47E-64 |
| 'EPAS1' | -1.27583168 | 8.78E-64 |
| 'GNE' | -2.172508162 | 9.09E-64 |
| 'RASIP1' | -2.366381693 | 1.34E-63 |
| 'TP53INP2' | -1.055271287 | 2.07E-63 |
| 'DUSP6' | -1.263505182 | 2.17E-63 |
| 'RIPK4' | -1.307714236 | 5.70E-63 |
| 'TLN2' | -1.286100016 | 1.33E-62 |
| 'SPPL2B' | -1.195787065 | 1.85E-62 |
| 'PBXIP1' | -1.034901075 | 3.11E-62 |
| 'HEG1' | -1.03925943 | 4.63E-62 |
| 'CNTNAP2' | -1.093360548 | 9.74E-62 |
| 'STEAP3' | -1.043027284 | 1.65E-61 |
| 'PPP1R18' | 1.227068909 | 2.09E-61 |
| 'NEK9' | -1.277704095 | 2.29E-61 |
| 'PDK3' | -1.415037499 | 2.42E-61 |
| 'UTRN' | -1.141797262 | 2.79E-61 |
| 'RAB11FIP4' | -1.182765347 | 5.26E-61 |
| 'ZNF618' | -1.376932894 | 6.01E-61 |
| 'CDK18' | -1.761511892 | 6.44E-61 |
| 'SEMA6B' | -1.652834614 | 1.18E-60 |
| 'ZNF385A' | 1.561617411 | 2.53E-60 |
| 'TMEM87B' | -1.117968919 | 2.82E-60 |
| 'ANK3' | -1.689659879 | 3.07E-60 |
| 'FGG' | -2.650764559 | 4.64E-60 |
| 'MICB' | 1.2891689 | 1.26E-59 |
| 'SIPA1L2' | -1.546965237 | 3.58E-59 |
| 'SEMA3F' | -1.893084796 | 3.66E-59 |
| 'PRSS2' | -1.093774938 | 8.72E-59 |
| 'PPM1H' | -1.462432025 | 1.42E-58 |
| 'ARNT' | -1.148270636 | 3.26E-58 |
| 'HS2ST1' | -1.012065066 | 5.23E-58 |
| 'NFKB2' | 1.001883414 | 5.70E-58 |
| 'TBL1X' | -1.093327186 | 9.40E-58 |
| 'KLF11' | -1.527646985 | 2.21E-57 |
| 'COL4A1' | -2.282553857 | 1.52E-56 |
| 'KIFC2' | -1.333977658 | 1.80E-56 |
| 'COL1A1' | -1.377804593 | 1.81E-56 |
| 'NCEH1' | 1.303669616 | 1.59E-55 |
| 'NLGN2' | -1.375039431 | 1.78E-55 |
| 'FMOD' | -1.802931735 | 4.51E-55 |
| 'TNKS2' | -1.08061846 | 6.05E-55 |
| 'PROM2' | -1.800368492 | 6.28E-55 |
| 'ANK1' | -2.502500341 | 8.89E-55 |
| 'JMJD6' | 1.324855805 | 7.37E-54 |
| 'MYLIP' | -1.913232122 | 1.21E-53 |
| 'TUBA4A' | 2.08788499 | 1.72E-53 |
| 'BMF' | -2.017671026 | 2.11E-53 |
| 'RFX5' | -1.200794787 | 4.68E-53 |
| 'TSPAN1' | 1.48370831 | 4.74E-53 |
| 'CYFIP2' | -1.654636029 | 4.99E-53 |
| 'SMYD2' | -1.302001391 | 1.91E-52 |
| 'SARM1' | -2.932313192 | 2.26E-52 |
| 'BIRC3' | 2.426897654 | 2.35E-52 |
| 'TMEM94' | -1.085792832 | 2.48E-52 |
| 'MYL7' | 2.923544494 | 5.01E-52 |
| 'PELI1' | -1.309124747 | 6.93E-52 |
| 'DNMT3A' | -1.178282418 | 8.75E-52 |
| 'SUSD4' | -2.017487427 | 1.39E-51 |
| 'FLG' | -1.005053228 | 1.54E-51 |
| 'ZNRF3' | -1.007948753 | 1.55E-51 |
| 'ANO9' | -1.275468784 | 1.74E-51 |
| 'SGSM2' | -1.043541427 | 2.59E-51 |
| 'C9orf152' | -2.119801522 | 8.27E-51 |
| 'GALNT3' | 1.28617807 | 2.01E-50 |
| 'RASGRP1' | -1.539158811 | 3.02E-50 |
| 'ZNF66' | -1.040133187 | 3.69E-50 |
| 'SYNPR' | -1.709240499 | 5.06E-50 |
| 'CAMK2D' | -1.121548206 | 5.33E-50 |
| 'GATD3B' | -1.173468521 | 7.78E-50 |
| 'ABCA1' | -2.736965594 | 1.16E-49 |
| 'ZNF362' | -1.279223644 | 2.25E-49 |
| 'TBC1D5' | -1.579359794 | 2.23E-48 |
| 'ACSF2' | -1.334342265 | 5.51E-48 |
| 'ZMYND8' | -1.102783641 | 3.49E-47 |
| 'KREMEN1' | -1.096945556 | 4.49E-47 |
| 'TLE2' | -1.589848856 | 6.20E-47 |
| 'ASNS' | -1.224749592 | 8.07E-47 |
| 'MED12' | -1.044699807 | 1.25E-46 |
| 'KLHDC2' | -1.216590448 | 1.51E-46 |
| 'LRIG1' | -1.029443778 | 1.54E-46 |
| 'MERTK' | -1.691877705 | 1.11E-45 |
| 'TMEM135' | -1.611323087 | 1.63E-45 |
| 'PSG1' | -2.007847862 | 5.39E-45 |
| 'SPRR1A' | 4.170751051 | 7.04E-45 |
| 'MECOM' | -1.55359833 | 1.20E-44 |
| 'GPC4' | -1.162938571 | 1.27E-44 |
| 'MAB21L4' | 1 | 1.50E-44 |
| 'SNCAIP' | -3.356693513 | 1.80E-44 |
| 'PCED1B' | -2.085836169 | 2.98E-44 |
| 'HABP4' | -1.394710475 | 3.06E-44 |
| 'FGB' | -2.88678939 | 4.42E-44 |
| 'UPP1' | 1.597227215 | 5.40E-44 |
| 'HDAC6' | -1.413043449 | 9.53E-44 |
| 'IL17RE' | -1.314613193 | 1.00E-43 |
| 'TRAPPC9' | -1.17531826 | 1.04E-43 |
| 'FAM210B' | -1.085450433 | 1.49E-43 |
| 'PGGHG' | -1.525293675 | 1.53E-43 |
| 'RHBDF2' | -1.209084776 | 5.80E-43 |
| 'SULT2B1' | -1.533604195 | 6.58E-43 |
| 'EP300' | -1.033735638 | 8.67E-43 |
| 'SHC2' | -1.85160816 | 1.45E-42 |
| 'KIAA0319L' | -1.012508995 | 1.77E-42 |
| 'NFKBIZ' | -2.241840184 | 1.78E-42 |
| 'RASSF1' | 1.55817752 | 1.87E-42 |
| 'NR1D2' | -1.207181171 | 2.59E-42 |
| 'SBK1' | -1.56315813 | 3.52E-42 |
| 'ID3' | -1.169735733 | 5.14E-42 |
| 'TMPRSS2' | -2.215563083 | 1.41E-41 |
| 'SMPDL3B' | -1.255060335 | 1.71E-41 |
| 'CADM1' | -1.318645511 | 2.36E-41 |
| 'TMEM229B' | -2.50371218 | 2.38E-40 |
| 'ATP8A1' | -1.488618632 | 2.55E-40 |
| 'TERF1' | -1.451106754 | 4.79E-40 |
| 'HRNR' | -1.590015728 | 6.39E-40 |
| 'JOSD2' | 1.062823396 | 1.26E-39 |
| 'WEE1' | -1.017680308 | 1.60E-39 |
| 'SLCO2B1' | -2 | 1.61E-39 |
| 'SLC39A10' | -1.028216242 | 1.71E-39 |
| 'MAP3K14' | 1.411024437 | 2.05E-39 |
| 'RIMKLB' | -1.97783835 | 7.65E-39 |
| 'ETS1' | 1.311280851 | 1.04E-38 |
| 'R3HDM2' | -1.273018494 | 1.55E-38 |
| 'SFI1' | -1.91186909 | 4.98E-38 |
| 'PABPC1L' | -1.86090439 | 5.15E-38 |
| 'EMP1' | 1.156881726 | 6.59E-38 |
| 'ETV6' | -1.170945656 | 7.70E-38 |
| 'ARHGEF17' | -1.217523382 | 9.33E-38 |
| 'MANBA' | -1.152814671 | 1.05E-37 |
| 'PLEKHH1' | -1.305345211 | 1.95E-37 |
| 'TNFAIP3' | 1.736965594 | 2.86E-37 |
| 'PSG2' | -1.604290762 | 2.94E-37 |
| 'PLEKHA7' | -1.060855205 | 4.74E-37 |
| 'CASP7' | -1.351628329 | 5.44E-37 |
| 'C19orf54' | -1.125862956 | 5.95E-37 |
| 'SIK1' | -2.785495488 | 1.04E-36 |
| 'TMEM45B' | -1.54320138 | 1.04E-36 |
| 'SMAD6' | -1.69653073 | 1.44E-36 |
| 'VSIG10' | -1.037357078 | 2.81E-36 |
| 'BAHCC1' | -1.756728849 | 3.84E-36 |
| 'FZD6' | -1.235247226 | 5.84E-36 |
| 'CHI3L1' | 1.070041732 | 7.84E-36 |
| 'RELL1' | -1.158531497 | 9.05E-36 |
| 'PLXNB3' | -1.536405681 | 1.63E-35 |
| 'CA11' | -1.514141164 | 1.68E-35 |
| 'KIF26B' | -1.504994196 | 1.88E-35 |
| 'ANXA9' | -1.181025995 | 2.08E-35 |
| 'MROH6' | -1.146280574 | 3.40E-35 |
| 'CADPS2' | -1.596233613 | 3.78E-35 |
| 'PCED1A' | -1.178713747 | 5.14E-35 |
| 'MNT' | -1.101086125 | 5.78E-35 |
| 'UBE2L6' | -1.072839374 | 5.78E-35 |
| 'TSPYL4' | -1.476925886 | 6.85E-35 |
| 'MTHFR' | -1.146976276 | 9.72E-35 |
| 'FGD3' | -1.555061015 | 1.40E-34 |
| 'STK40' | 1.050281754 | 3.00E-34 |
| 'MAML3' | -2.593230117 | 3.33E-34 |
| 'ENOX1' | -1.018430705 | 3.79E-34 |
| 'NTM' | -3.416646752 | 5.45E-34 |
| 'PALM3' | -1.51653736 | 5.69E-34 |
| 'FGFR2' | -1.04677976 | 6.91E-34 |
| 'AMACR' | -1.182052692 | 1.10E-33 |
| 'PLAT' | -1.928182839 | 1.40E-33 |
| 'PDCD4' | -1.712576697 | 3.20E-33 |
| 'YPEL3' | -1.192169177 | 3.33E-33 |
| 'CPT2' | -1.233919855 | 4.25E-33 |
| 'IL1RN' | 1.635260166 | 6.07E-33 |
| 'DBP' | -1.479596228 | 6.40E-33 |
| 'PRR15L' | -2.090288885 | 1.68E-32 |
| 'PSG6' | -2.931765264 | 2.78E-32 |
| 'LRRC56' | -1.022331367 | 3.39E-32 |
| 'MRS2' | -1.044937405 | 4.25E-32 |
| 'RHPN1' | -2.595158268 | 4.96E-32 |
| 'LRG1' | -1.711202498 | 5.83E-32 |
| 'NTN4' | 1.803199293 | 7.43E-32 |
| 'C19orf66' | -1.026391499 | 8.25E-32 |
| 'PLEKHH2' | -1.392317423 | 1.85E-31 |
| 'CUL9' | -1.58024009 | 1.89E-31 |
| 'C1RL' | -1.512175563 | 2.37E-31 |
| 'MCCC1' | -1.272286719 | 3.12E-31 |
| 'ATG4D' | 1.031813002 | 5.92E-31 |
| 'CNNM2' | -1.43718554 | 9.96E-31 |
| 'NTN1' | -1.392317423 | 1.35E-30 |
| 'SPRR2A' | 4.347558695 | 1.87E-30 |
| 'DEGS2' | -4.10433666 | 2.65E-30 |
| 'DHRS2' | 1.179050917 | 9.20E-30 |
| 'MAP3K8' | -1.181555439 | 9.44E-30 |
| 'SPG11' | -1.078988296 | 9.71E-30 |
| 'BRF2' | 1.232660757 | 1.20E-29 |
| 'ASAP3' | -2.331843564 | 1.40E-29 |
| 'DOCK11' | -1.35799735 | 2.43E-29 |
| 'DEPP1' | -1.84434913 | 3.22E-29 |
| 'PLEKHA4' | -1.166293751 | 4.03E-29 |
| 'SEMA3C' | -1.260000346 | 8.43E-29 |
| 'DMBT1' | -3.498250868 | 8.70E-29 |
| 'PHF21A' | -1.157852169 | 8.84E-29 |
| 'ALDH6A1' | -1.194728392 | 9.47E-29 |
| 'PROSER3' | -1.055577348 | 1.04E-28 |
| 'BRWD3' | -1.082687282 | 1.41E-28 |
| 'PPFIBP2' | -1.106915204 | 1.49E-28 |
| 'PLPP3' | -1.443606651 | 1.64E-28 |
| 'MAN2B2' | -1.034995421 | 1.78E-28 |
| 'PSG9' | -2.431049817 | 1.94E-28 |
| 'SLC7A11' | -1.592342031 | 1.99E-28 |
| 'IQSEC1' | -1.012600037 | 2.39E-28 |
| 'LCP1' | 1.996116566 | 4.52E-28 |
| 'NR1D1' | -1.130597123 | 5.74E-28 |
| 'NCF2' | 1.777891099 | 6.39E-28 |
| 'NOTCH3' | -1.572736198 | 8.24E-28 |
| 'BCAS3' | -1.384359901 | 8.61E-28 |
| 'PCMTD2' | -1.321928095 | 1.20E-27 |
| 'NFKBIE' | 1.310872906 | 1.36E-27 |
| 'ARHGAP29' | 1.103669929 | 1.42E-27 |
| 'KCNQ1' | -1.745595899 | 1.93E-27 |
| 'UNKL' | 1.199982235 | 2.42E-27 |
| 'MANSC1' | -1.192967648 | 3.41E-27 |
| 'MAP2K6' | -2.738642172 | 5.11E-27 |
| 'DNAJC22' | -1.153185146 | 5.75E-27 |
| 'PHLPP1' | -1.529160709 | 6.74E-27 |
| 'MBNL3' | -1.157541277 | 8.90E-27 |
| 'BNIP3L' | -1.113604109 | 1.21E-26 |
| 'SLC27A1' | -1.791943196 | 1.40E-26 |
| 'OAS2' | -1.520422249 | 1.57E-26 |
| 'ACBD4' | -1.770753908 | 3.13E-26 |
| 'RFLNA' | 2.155517578 | 3.20E-26 |
| 'ORAI3' | -1.883485991 | 3.92E-26 |
| 'CH507-42P11.6' | -1.906890596 | 4.05E-26 |
| 'TMC4' | -1.068493605 | 4.23E-26 |
| 'TSPYL2' | -1.56358885 | 4.27E-26 |
| 'FAM241B' | 1.434937057 | 5.50E-26 |
| 'SYNGAP1' | -1.534174391 | 7.14E-26 |
| 'PSD3' | -1.305249354 | 8.78E-26 |
| 'HK1' | -1.286089642 | 9.82E-26 |
| 'DMXL2' | -1.008664861 | 1.17E-25 |
| 'S100P' | 1.343713378 | 3.01E-25 |
| 'GPAT3' | 2.168122759 | 4.12E-25 |
| 'SLCO2A1' | -1.891623839 | 4.22E-25 |
| 'THRA' | -1.158380663 | 8.56E-25 |
| 'C6orf223' | -3.502500341 | 8.63E-25 |
| 'ACTA2' | -1.501061243 | 1.19E-24 |
| 'SLC4A8' | -1.904652123 | 1.48E-24 |
| 'ALDH5A1' | -1.079955305 | 3.59E-24 |
| 'PLEKHB1' | -1.381391255 | 3.92E-24 |
| 'BMP4' | -1.288009269 | 4.71E-24 |
| 'SOX21' | -2.181897643 | 5.90E-24 |
| 'TEF' | -1.119695066 | 6.76E-24 |
| 'ZNF296' | 1.083926954 | 8.25E-24 |
| 'TBCK' | -1.457303226 | 8.34E-24 |
| 'FAM214B' | 1.199122642 | 9.05E-24 |
| 'ICA1' | -1.034473527 | 1.63E-23 |
| 'PARP10' | -2.150362735 | 1.68E-23 |
| 'CPA4' | 1.937478915 | 1.84E-23 |
| 'PRKCG' | -2.089531222 | 2.23E-23 |
| 'GRK3' | -1.126335733 | 2.28E-23 |
| 'SLC2A10' | -2.276331228 | 2.78E-23 |
| 'SORBS2' | -1.694935343 | 4.03E-23 |
| 'IKBKB' | -1.114152307 | 4.39E-23 |
| 'ACER2' | -1.397822944 | 5.00E-23 |
| 'SMIM14' | -1.356303674 | 5.43E-23 |
| 'ANO8' | -1.31495854 | 6.25E-23 |
| 'ICAM4' | -1.226229378 | 7.57E-23 |
| 'LGALS4' | -2.180081616 | 8.15E-23 |
| 'BDH2' | -1.501163274 | 8.55E-23 |
| 'NDRG1' | 1.011705501 | 9.54E-23 |
| 'LYPD3' | 1.461534162 | 1.19E-22 |
| 'SRCIN1' | -1.567040593 | 1.34E-22 |
| 'ZNF462' | -1.078002512 | 1.56E-22 |
| 'RBBP8NL' | -1.300321639 | 1.78E-22 |
| 'FIG4' | -1.301090632 | 1.95E-22 |
| 'IFITM2' | -2.326429487 | 2.39E-22 |
| 'DIS3L2' | -1.065227623 | 4.13E-22 |
| 'HEXD' | -1.497622835 | 9.42E-22 |
| 'PCCA' | -1.193331476 | 9.76E-22 |
| 'ANKRD36B' | -1.3742835 | 1.31E-21 |
| 'SEMA4G' | -1.162528273 | 2.09E-21 |
| 'VIM' | -1.099384914 | 2.58E-21 |
| 'METTL7A' | -3.079727192 | 2.83E-21 |
| 'ARHGEF19' | -2.624490865 | 3.08E-21 |
| 'HOXB3' | -1.031194622 | 3.93E-21 |
| 'SPSB1' | 1.1734164 | 4.33E-21 |
| 'PER3' | -1.061400545 | 5.69E-21 |
| 'SPRY2' | -1.075948853 | 6.95E-21 |
| 'TMEM8B' | -2.152951923 | 7.76E-21 |
| 'PSG7' | -2.335733894 | 8.24E-21 |
| 'E2F2' | -1.417611443 | 8.26E-21 |
| 'BRSK1' | -1.206172069 | 9.09E-21 |
| 'PAQR6' | -2.790431619 | 9.24E-21 |
| 'MX2' | -1.187509314 | 9.24E-21 |
| 'LCK' | -1.430041924 | 9.77E-21 |
| 'KDM7A' | -1.406424369 | 1.05E-20 |
| 'IQCB1' | -1.013079827 | 1.65E-20 |
| 'ACPP' | -3 | 2.25E-20 |
| 'KIAA0556' | -1.091315167 | 2.83E-20 |
| 'PCDHB13' | -1.671674134 | 2.99E-20 |
| 'PIP5K1B' | -1.077593121 | 3.09E-20 |
| 'TENT5B' | 1.006194492 | 3.84E-20 |
| 'PHOSPHO2-KLHL23' | -2.142957954 | 3.86E-20 |
| 'UNK' | -1.134301092 | 5.26E-20 |
| 'GCNT1' | -1.889817082 | 5.33E-20 |
| 'NRBP2' | -1.490682553 | 9.86E-20 |
| 'BCL9' | -1.006313774 | 1.16E-19 |
| 'PPAN-P2RY11' | -1.190632483 | 1.24E-19 |
| 'CDRT4' | -7.118941073 | 1.32E-19 |
| 'TMCO6' | -1.122436076 | 1.47E-19 |
| 'RPS6KA2' | -1.331676071 | 1.60E-19 |
| 'ATP7A' | -1.325575872 | 1.64E-19 |
| 'GOLGA6L9' | -1.670353992 | 1.66E-19 |
| 'CASTOR3' | -1.433756697 | 1.79E-19 |
| 'IGFL2' | 1.118019333 | 1.87E-19 |
| 'LRRC37A' | -1.557376669 | 2.00E-19 |
| 'ZSWIM5' | -1.551576332 | 2.35E-19 |
| 'CAPN8' | -1.754300205 | 2.44E-19 |
| 'TLE4' | -1.290255875 | 3.19E-19 |
| 'ITGA7' | -1.386468347 | 3.46E-19 |
| 'TENT5C' | -1.267651034 | 4.15E-19 |
| 'RIC3' | -1.206278296 | 5.04E-19 |
| 'HPN' | -3.106199404 | 5.85E-19 |
| 'LOC100130370' | -2.371968777 | 5.87E-19 |
| 'LOC101059906' | -1.819253505 | 6.04E-19 |
| 'NPIPB2' | -1.452311872 | 9.04E-19 |
| 'GALNT4' | -1.623956632 | 9.11E-19 |
| 'ST6GALNAC1' | -2.584962501 | 9.14E-19 |
| 'SLC9A6' | -1.002573944 | 1.06E-18 |
| 'SHANK2' | -1.149513541 | 1.24E-18 |
| 'NRDE2' | -1.009429413 | 1.42E-18 |
| 'CYP2J2' | -1.215587243 | 1.59E-18 |
| 'CCDC144A' | -1.006426269 | 1.62E-18 |
| 'SPRR2F' | 5.977279923 | 1.76E-18 |
| 'ABCA3' | -1.225881407 | 2.03E-18 |
| 'DHTKD1' | -1.045596866 | 2.23E-18 |
| 'DES' | -2.042041771 | 3.16E-18 |
| 'RNF8' | -1.043501639 | 3.46E-18 |
| 'MMAB' | -1.131023651 | 3.83E-18 |
| 'ATP8B3' | -1.985692163 | 3.94E-18 |
| 'EGR2' | -5.011227255 | 4.51E-18 |
| 'CRYAB' | 3.464467209 | 4.68E-18 |
| 'SCRN2' | -1.34533795 | 5.78E-18 |
| 'PRPF40B' | -1.555742362 | 5.98E-18 |
| 'BCKDHB' | -1.104182038 | 9.58E-18 |
| 'SPRY1' | -1.349491734 | 9.68E-18 |
| 'C1R' | -1.761840263 | 1.09E-17 |
| 'GLCCI1' | -1.13058411 | 1.15E-17 |
| 'LOC107984841' | -1.472923855 | 1.20E-17 |
| 'CRELD1' | -1.211940057 | 1.21E-17 |
| 'ARPIN' | -1.027783157 | 1.64E-17 |
| 'NFE2' | -2.547487795 | 1.73E-17 |
| 'LTB4R' | -1.379315577 | 1.77E-17 |
| 'PARP15' | -1.112894056 | 2.79E-17 |
| 'TTN' | -2.700439718 | 3.36E-17 |
| 'TECPR1' | -1.235316987 | 3.47E-17 |
| 'GGT6' | -2.547065893 | 4.31E-17 |
| 'SHC3' | -1.732519689 | 4.43E-17 |
| 'U2AF1L5' | -2.088968524 | 4.72E-17 |
| 'ITPR2' | -1.111361109 | 5.26E-17 |
| 'RGS5' | -2.493539473 | 5.49E-17 |
| 'AMOT' | -3.655351829 | 5.79E-17 |
| 'PKD2' | -1.059706246 | 6.26E-17 |
| 'TRAPPC6A' | -1.318237048 | 6.45E-17 |
| 'KCNJ4' | -1.719055396 | 7.27E-17 |
| 'THBS3' | -1.489714186 | 7.99E-17 |
| 'TBC1D3L' | -1.520961501 | 8.12E-17 |
| 'IMPACT' | -1.190822342 | 8.72E-17 |
| 'KLHL25' | 1.029747343 | 1.07E-16 |
| 'MEIS3' | -1.057898384 | 2.02E-16 |
| 'LRRC75A' | -1.229822627 | 2.13E-16 |
| 'OLFM2' | -1.681715734 | 2.31E-16 |
| 'USH1C' | -1.092691898 | 2.46E-16 |
| 'SPRR1B' | 8.985841937 | 2.50E-16 |
| 'DUSP22' | -1.388301117 | 2.94E-16 |
| 'ANKRD36' | -1.128324097 | 3.53E-16 |
| 'MAPRE3' | 1.269366655 | 4.38E-16 |
| 'BTN3A2' | -1.010077073 | 4.78E-16 |
| 'KAT6B' | -1 | 4.81E-16 |
| 'MAMDC4' | -1.813403967 | 6.57E-16 |
| 'MBTD1' | -1.098403704 | 8.85E-16 |
| 'KCNC3' | -2.897240426 | 1.03E-15 |
| 'APOL1' | -1.275341301 | 1.05E-15 |
| 'MGAM2' | -1.444302094 | 1.09E-15 |
| 'GABBR1' | -1.477321778 | 1.26E-15 |
| 'PIWIL2' | -2.137503524 | 1.27E-15 |
| 'OGDHL' | -2.417852515 | 1.31E-15 |
| 'TMEM44' | -1.078656944 | 1.86E-15 |
| 'OPRL1' | -2.298341275 | 2.38E-15 |
| 'ASIC1' | -1.419903254 | 2.45E-15 |
| 'CORO7-PAM16' | -6.209453366 | 2.65E-15 |
| 'UVSSA' | -1.274916029 | 4.63E-15 |
| 'PROX1' | -1.370837695 | 4.88E-15 |
| 'OSBPL7' | -1.316796987 | 5.14E-15 |
| 'RGS8' | -3.209453366 | 6.15E-15 |
| 'KBTBD8' | 1.9224634 | 6.16E-15 |
| 'TBC1D3I' | -1.711874613 | 6.77E-15 |
| 'GPR75-ASB3' | -2.262094845 | 6.77E-15 |
| 'ZNF839' | -1.023612557 | 6.77E-15 |
| 'PRDM11' | -1.04872604 | 7.03E-15 |
| 'IFT140' | -1.195688098 | 8.01E-15 |
| 'TSHZ2' | -3.584962501 | 8.31E-15 |
| 'BCL6' | -1.565333694 | 8.68E-15 |
| 'JMY' | -1.161463423 | 9.75E-15 |
| 'ADGRA2' | -2.754887502 | 1.08E-14 |
| 'VIPR1' | -1.598018654 | 1.10E-14 |
| 'TRIB2' | -1.21081607 | 1.13E-14 |
| 'CREB3L1' | -1.13597767 | 1.66E-14 |
| 'EPHB3' | -1.078387899 | 1.70E-14 |
| 'POLR3G' | 1.129661131 | 1.73E-14 |
| 'DNAH6' | -2.093109404 | 1.82E-14 |
| 'LTBP2' | -1.331843564 | 2.10E-14 |
| 'LOC100129484' | -1.740031897 | 2.36E-14 |
| 'LOC102724200' | 6.339850003 | 2.37E-14 |
| '105379045' | -1.652076697 | 2.37E-14 |
| 'RASA4B' | -2.601450624 | 2.42E-14 |
| 'ZKSCAN2' | -1.076621282 | 2.46E-14 |
| 'LHPP' | -1.643497712 | 3.13E-14 |
| 'DOC2A' | -2.393663848 | 3.18E-14 |
| 'DNAJB4' | 1.633129783 | 3.51E-14 |
| 'BANP' | 1.132260929 | 3.72E-14 |
| 'ACVR2B' | -1.069421401 | 3.85E-14 |
| 'LOC101060341' | -1.177873755 | 3.93E-14 |
| 'CEMIP' | -2.523561956 | 4.20E-14 |
| 'PCMTD1' | -1.187278568 | 4.20E-14 |
| 'RAB26' | -3.093109404 | 4.91E-14 |
| 'FBXL8' | -2.191531458 | 5.31E-14 |
| 'KRT7' | 1.195991106 | 6.30E-14 |
| 'ALDOC' | -1.582346553 | 6.90E-14 |
| 'GSAP' | -1.289186694 | 7.49E-14 |
| 'TMEM189-UBE2V1' | 1.155541083 | 8.49E-14 |
| 'ARHGAP33' | -1.441915883 | 8.92E-14 |
| 'C6orf222' | -2.795859283 | 8.97E-14 |
| 'CDKN1C' | -2.487665299 | 1.03E-13 |
| 'UPK3BL2' | -1.31024629 | 1.13E-13 |
| 'ZNF608' | -1.13876407 | 1.19E-13 |
| 'LOC112268114' | -2.492768803 | 1.26E-13 |
| 'NPIPB6' | -1.078517209 | 1.42E-13 |
| 'FGF20' | -3.188883159 | 1.64E-13 |
| 'GFPT2' | 2.15277028 | 1.81E-13 |
| 'NPY4R2' | -5.807354922 | 1.83E-13 |
| 'IFT122' | -1.175442006 | 1.87E-13 |
| 'CA13' | -1.156342029 | 1.92E-13 |
| 'DLG4' | -1.833706249 | 1.99E-13 |
| 'SLC40A1' | -1.286881148 | 2.69E-13 |
| 'TBC1D3G' | -1.839535328 | 2.73E-13 |
| 'POLR3GL' | -1.914270126 | 2.88E-13 |
| 'SPTB' | -1.422691072 | 2.99E-13 |
| 'SEMA7A' | 1.027691734 | 3.55E-13 |
| 'APBB3' | -1.384990363 | 4.32E-13 |
| 'KRT6B' | 1.974374192 | 4.72E-13 |
| 'VGLL1' | -1.393565969 | 5.19E-13 |
| 'CPT1C' | -1.045442971 | 5.29E-13 |
| 'PAGE4' | -1.03157596 | 5.33E-13 |
| 'SLC26A9' | -3.68182404 | 5.46E-13 |
| 'SLC9A4' | -2.546488353 | 6.00E-13 |
| 'CPT1B' | -1.213734061 | 6.94E-13 |
| 'ELOVL7' | -1.256595845 | 7.53E-13 |
| 'SYTL4' | 1.037637657 | 7.82E-13 |
| 'GRB14' | -1.29072614 | 8.42E-13 |
| 'LRRC37A3' | -1.244702583 | 9.18E-13 |
| 'RWDD2B' | -1.018223759 | 1.19E-12 |
| 'PCDHGB7' | -2.282035368 | 1.22E-12 |
| 'TBX4' | -3 | 1.30E-12 |
| 'OSBPL5' | -1.689659879 | 1.36E-12 |
| 'LRP4' | -1.240051088 | 1.48E-12 |
| 'COL18A1' | -1.708537186 | 1.58E-12 |
| 'TTC7B' | -1.165174602 | 1.65E-12 |
| 'LOC112268238' | -1.562766755 | 1.87E-12 |
| 'CELSR3' | -1.30580843 | 2.22E-12 |
| 'VILL' | -1.032158668 | 2.65E-12 |
| 'RNF144B' | -1.723482365 | 2.74E-12 |
| 'GLS2' | -2.86507042 | 2.90E-12 |
| 'ACAD10' | -1.452858965 | 3.02E-12 |
| 'HAGHL' | -1.175086707 | 3.44E-12 |
| 'ARHGAP44' | -1.15417093 | 3.44E-12 |
| 'C2CD4A' | -1.966052668 | 3.69E-12 |
| 'NOX1' | -4.123382416 | 3.83E-12 |
| 'CNTRL' | -1.017629894 | 4.24E-12 |
| 'ACCS' | -1.366381693 | 4.82E-12 |
| 'MITF' | 1.357069901 | 5.42E-12 |
| 'TSTD3' | -1.255257055 | 5.68E-12 |
| 'UBD' | 2.020037753 | 6.26E-12 |
| 'IFT172' | -1.847996907 | 7.61E-12 |
| 'TRIM54' | 5.741466986 | 8.07E-12 |
| 'ALDH3B1' | -1.086340849 | 8.19E-12 |
| 'PER2' | -1.051074185 | 8.33E-12 |
| 'ATAT1' | -1.228531558 | 8.69E-12 |
| 'CDK19' | -1.00625899 | 1.14E-11 |
| 'SLC31A2' | 1.050626073 | 1.42E-11 |
| 'ANKRD36C' | -1 | 1.54E-11 |
| 'PLP1' | -5.247927513 | 1.56E-11 |
| 'CCDC24' | -1.071364451 | 1.58E-11 |
| 'ODAM' | -2.431339312 | 1.60E-11 |
| 'WDR27' | -1.084268917 | 2.03E-11 |
| 'ITIH4' | -2.561878888 | 2.34E-11 |
| 'EHF' | -1.631132682 | 2.45E-11 |
| 'RPS10-NUDT3' | -6.303780748 | 2.52E-11 |
| 'TGFB3' | -2.765534746 | 2.87E-11 |
| 'NOXA1' | -1.432959407 | 3.04E-11 |
| 'LYSMD3' | 1.030650213 | 3.05E-11 |
| 'GTDC1' | -1.346705668 | 3.19E-11 |
| 'LMCD1' | -1.846087317 | 4.36E-11 |
| 'MICALCL' | -1.356934545 | 4.64E-11 |
| '100996414' | -3.420957471 | 4.93E-11 |
| 'CXCL2' | -1.862496476 | 5.08E-11 |
| 'ALDH3B2' | -6.129283017 | 5.10E-11 |
| 'C20orf96' | -1.315614321 | 5.59E-11 |
| 'AQP5' | -2.811927652 | 5.81E-11 |
| 'APLP1' | -1.443445914 | 6.57E-11 |
| 'RAB40B' | -1.200077835 | 6.59E-11 |
| 'HSD17B2' | -1.698042551 | 6.80E-11 |
| 'CCDC78' | -1.959358016 | 7.66E-11 |
| 'KLHL24' | -1.407657969 | 7.89E-11 |
| 'GDPD3' | 1.453126335 | 7.89E-11 |
| 'TRANK1' | -1.069262662 | 8.09E-11 |
| 'SH3BGRL' | -1.559034036 | 1.06E-10 |
| 'LOC644634' | 5.781359714 | 1.07E-10 |
| 'IZUMO1' | -2.575684687 | 1.12E-10 |
| 'BTN3A1' | -1.578076115 | 1.20E-10 |
| 'ZSCAN4' | -1.266465311 | 1.23E-10 |
| 'ZNF765-ZNF761' | -1.224966365 | 1.31E-10 |
| 'SOCS1' | 1.565400234 | 1.34E-10 |
| 'ALOXE3' | 1.578938713 | 1.34E-10 |
| 'KAT2B' | -1.068947354 | 1.34E-10 |
| 'GPSM3' | -1.694497453 | 1.87E-10 |
| 'SELL' | -2.518728802 | 1.92E-10 |
| 'UNC93A' | -2.115477217 | 1.94E-10 |
| 'PAK6' | -5.523561956 | 2.07E-10 |
| 'KLHL23' | 1.057715498 | 2.08E-10 |
| 'CREB5' | -1.048094288 | 2.11E-10 |
| 'ANG' | -1.802726445 | 2.37E-10 |
| 'SCN1B' | -1.818452631 | 2.43E-10 |
| 'PMCH' | -1.634170025 | 2.47E-10 |
| 'WDCP' | 1.10433666 | 2.55E-10 |
| 'MLLT11' | 1.70435123 | 2.59E-10 |
| 'RAPGEFL1' | -1.155030786 | 3.01E-10 |
| 'KRT16' | 2.511645823 | 3.08E-10 |
| 'SERPIND1' | 2.769387072 | 3.23E-10 |
| 'XAF1' | -1.484121811 | 3.24E-10 |
| 'DENND6B' | -1.301380717 | 3.60E-10 |
| 'ZFAT' | -1.434628228 | 3.61E-10 |
| 'SLC25A35' | -1.304334035 | 3.79E-10 |
| 'SLC9A3' | -1.175638653 | 3.99E-10 |
| 'CCDC170' | -1.401362562 | 4.10E-10 |
| 'NPIPA1' | -1.072083014 | 4.64E-10 |
| 'ALOX5AP' | 2.560360527 | 5.11E-10 |
| 'GP6' | -1.574908836 | 5.45E-10 |
| 'ICOSLG' | 1.035046947 | 7.99E-10 |
| 'APOL4' | -1.761551232 | 8.86E-10 |
| 'KRT14' | 1.704701745 | 1.02E-09 |
| 'LOC102724219' | -3.184424571 | 1.12E-09 |
| 'RASA4' | -1.925999419 | 1.29E-09 |
| 'FBF1' | -1.159656666 | 1.32E-09 |
| 'BBS1' | -1.172389041 | 1.32E-09 |
| 'ZNF547' | 2.546488353 | 1.53E-09 |
| 'NRP2' | -1.441837559 | 1.57E-09 |
| 'PSG4' | -1.334026237 | 1.59E-09 |
| 'CREB3L4' | -1.220473944 | 1.59E-09 |
| 'SPDEF' | -2.867896464 | 1.61E-09 |
| 'FAM117B' | -1.068895081 | 1.70E-09 |
| 'ATP8A2' | 2.459431619 | 1.73E-09 |
| 'APOBEC3H' | -1.759948912 | 1.76E-09 |
| 'TRPV3' | 1.543142325 | 1.82E-09 |
| 'SRGAP3' | -1.761840263 | 2.15E-09 |
| 'SERPINB8' | 1.389042291 | 2.33E-09 |
| 'VMAC' | -1.609624555 | 2.38E-09 |
| 'TMEM53' | -1.062194762 | 2.53E-09 |
| 'BMP2' | 1.14989543 | 2.78E-09 |
| 'ILDR1' | -2.334419039 | 2.85E-09 |
| 'PLCG2' | -1.247927513 | 2.98E-09 |
| 'CPEB2' | 1.422233001 | 3.06E-09 |
| 'LBHD1' | -1.394661363 | 3.38E-09 |
| 'IL33' | -5.930737338 | 3.41E-09 |
| 'TDRD9' | 3.906890596 | 3.55E-09 |
| 'PVRIG' | -1.263034406 | 3.63E-09 |
| 'GPATCH11' | 1.014260693 | 4.04E-09 |
| 'GLDC' | -2.532495081 | 4.38E-09 |
| 'PRIMPOL' | -1.565790191 | 4.66E-09 |
| 'ARPC4-TTLL3' | -2.162271429 | 4.70E-09 |
| 'BCL2L2-PABPN1' | -1.36994961 | 4.97E-09 |
| 'ENO3' | -1.303883919 | 5.94E-09 |
| 'PLA2G4B' | -5.832890014 | 6.93E-09 |
| 'GOLGA8B' | -1.040414268 | 7.19E-09 |
| 'PCDHGA10' | -2.974004791 | 7.46E-09 |
| 'RASSF4' | -1.38502493 | 7.53E-09 |
| 'DUOX2' | -2.554588852 | 7.60E-09 |
| 'NLRP7' | -3.655351829 | 8.40E-09 |
| 'HSD3B1' | -2.924272674 | 9.22E-09 |
| 'LOC105379547' | -1.191481145 | 1.02E-08 |
| 'DNAH2' | -2.807354922 | 1.08E-08 |
| 'JAKMIP2' | -1.807354922 | 1.12E-08 |
| 'HIST2H2BE' | -1.268105395 | 1.17E-08 |
| 'TIRAP' | 1.261232163 | 1.22E-08 |
| 'ATG16L2' | -1.57634937 | 1.25E-08 |
| 'EAF2' | -1.630680801 | 1.25E-08 |
| 'CCDC136' | -1.671767328 | 1.25E-08 |
| 'RGN' | -1.411754915 | 1.27E-08 |
| 'IKZF2' | -2.280107919 | 1.28E-08 |
| 'LOC389831' | 2.36923381 | 1.29E-08 |
| 'VASN' | -1.255761171 | 1.32E-08 |
| 'DNM1' | -1.479469372 | 1.33E-08 |
| 'BTN3A3' | -2.060882242 | 1.34E-08 |
| 'PIGV' | -1.086550032 | 1.45E-08 |
| 'OMA1' | -1.381870635 | 1.53E-08 |
| 'NPIPA3' | -1.769387072 | 1.66E-08 |
| 'SPDYE2' | -1.108181652 | 1.85E-08 |
| 'BUB1B-PAK6' | 5.321928095 | 1.86E-08 |
| 'KBTBD4' | -1.051138849 | 1.86E-08 |
| 'LOC102724788' | -1.554588852 | 1.87E-08 |
| 'AXL' | -1.128007612 | 1.98E-08 |
| 'APOBEC2' | -2.584962501 | 2.10E-08 |
| 'PCYOX1L' | -1.591915261 | 2.24E-08 |
| 'GNAZ' | -1.584962501 | 2.64E-08 |
| 'LOC107983998' | -1.367839767 | 2.68E-08 |
| 'PLA2G6' | -1.781999348 | 2.81E-08 |
| 'NFIB' | -1.125530882 | 2.97E-08 |
| 'KLF9' | -2.115477217 | 3.67E-08 |
| 'RARRES3' | -2.387849488 | 3.98E-08 |
| 'MAML2' | -1.099535674 | 4.14E-08 |
| 'ZDHHC1' | -1.876851769 | 4.38E-08 |
| 'C1orf21' | -1.083416008 | 4.53E-08 |
| 'APOL6' | -1.415037499 | 4.57E-08 |
| 'TRPV6' | -1.40780593 | 4.57E-08 |
| 'NR4A2' | -1.767553914 | 4.62E-08 |
| 'NKPD1' | -2.530514717 | 5.11E-08 |
| 'DOCK4' | -1.192645078 | 5.27E-08 |
| 'GTF2IRD2B' | -1.394531844 | 5.61E-08 |
| 'DACT1' | -5.209453366 | 5.65E-08 |
| 'CBS' | -5.754887502 | 5.65E-08 |
| 'CRYBB3' | -1.394278939 | 5.68E-08 |
| 'APOD' | -1.373340668 | 5.70E-08 |
| 'CTF1' | -1.176077228 | 5.72E-08 |
| 'TBC1D3C' | 2.197939378 | 6.15E-08 |
| 'MED31' | 1.152990902 | 6.16E-08 |
| 'IGF1' | -2.2410081 | 6.57E-08 |
| 'SMIM19' | -1.404066381 | 6.73E-08 |
| 'GNGT2' | 7.247927513 | 6.74E-08 |
| 'MEGF6' | -1.695145418 | 6.99E-08 |
| 'CBR3' | 1.404390255 | 7.68E-08 |
| 'ARHGAP30' | -2 | 7.73E-08 |
| 'PIK3IP1' | -3.239465935 | 8.27E-08 |
| 'IL1R2' | 1.614108846 | 8.79E-08 |
| 'RNASE4' | -2.205904299 | 9.86E-08 |
| 'KIF12' | -1.350281291 | 9.94E-08 |
| 'S100A3' | 1.634715536 | 1.02E-07 |
| 'FOXO6' | -1.682809824 | 1.11E-07 |
| 'LRRC24' | -6.285402219 | 1.14E-07 |
| 'TATDN3' | -1.006919414 | 1.15E-07 |
| 'GSDMB' | -1.560957038 | 1.38E-07 |
| 'PRTG' | -1.070389328 | 1.46E-07 |
| 'PAN2' | -1.085167383 | 1.46E-07 |
| 'ZNF471' | -1.974004791 | 1.49E-07 |
| 'LPAR6' | -1.406625259 | 1.50E-07 |
| 'TESK2' | -1.023083613 | 1.56E-07 |
| 'UNC5B' | -1.278716028 | 1.59E-07 |
| 'NPIPB15' | -1.114367025 | 1.64E-07 |
| 'TCAF2' | -1.334984248 | 1.75E-07 |
| 'SOX6' | -2.087462841 | 1.81E-07 |
| 'EFHC1' | -1.278301162 | 1.96E-07 |
| 'TRIM34' | -1.942918905 | 1.98E-07 |
| 'LY6D' | -1.415037499 | 2.10E-07 |
| 'IRX5' | -1.779874186 | 2.21E-07 |
| 'NTF4' | -1.210452808 | 2.24E-07 |
| 'DNAJC25-GNG10' | 6.459431619 | 2.44E-07 |
| 'PLCH1' | -1.584962501 | 2.46E-07 |
| 'ADAMTS14' | -2.688055994 | 3.02E-07 |
| 'ERV3-1' | -1.356485317 | 3.10E-07 |
| 'CREBRF' | -1.533978572 | 3.22E-07 |
| 'SPRR3' | 3.445559444 | 3.57E-07 |
| 'KCNN4' | -1.316027493 | 3.68E-07 |
| 'ACACB' | -1.075948853 | 3.69E-07 |
| 'ESRRG' | -4.700439718 | 3.83E-07 |
| 'CTSK' | -1.800230488 | 3.96E-07 |
| 'MMRN2' | -1.15324626 | 4.49E-07 |
| 'SMIM11A' | 3.201633861 | 4.70E-07 |
| 'JAG2' | -1.378511623 | 5.42E-07 |
| 'SLC24A1' | -1.371968777 | 5.48E-07 |
| 'DZANK1' | -1.222392421 | 5.61E-07 |
| 'ZNF420' | 1.317970081 | 5.90E-07 |
| 'RCN3' | -1.511252815 | 5.97E-07 |
| 'EME2' | -1.231162631 | 6.27E-07 |
| 'TUBB6' | -2.86507042 | 6.39E-07 |
| 'CPAMD8' | -3.247927513 | 7.73E-07 |
| 'DLX4' | -1.274794119 | 7.77E-07 |
| 'RNF207' | -1.296393003 | 8.31E-07 |
| 'PPM1L' | -1.222392421 | 8.31E-07 |
| 'FADS1' | -2.485426827 | 8.36E-07 |
| 'TMEM38A' | -1.017141133 | 8.41E-07 |
| 'GPX5' | -1.537797393 | 8.44E-07 |
| 'ATP8B1' | -1.075288127 | 8.78E-07 |
| 'ANKRD20A2' | -5.129283017 | 9.19E-07 |
| 'HCAR2' | -5.807354922 | 9.19E-07 |
| 'CDKN2C' | -1.246857662 | 9.29E-07 |
| 'ASB2' | 3.129283017 | 9.74E-07 |
| 'ACSM3' | -2.078002512 | 1.10E-06 |
| 'ARGFX' | -1.765534746 | 1.10E-06 |
| 'NPHP4' | -1.023083613 | 1.18E-06 |
| 'PSAPL1' | 1.362570079 | 1.22E-06 |
| 'SFXN2' | -1.057333175 | 1.24E-06 |
| 'PRRT1' | -1.807354922 | 1.25E-06 |
| 'MED12L' | -1.118644496 | 1.26E-06 |
| 'MCTP1' | -1.295455884 | 1.27E-06 |
| 'LOC112268219' | -1.08813091 | 1.31E-06 |
| 'KLHDC7A' | -2.080919995 | 1.31E-06 |
| 'LOC102724951' | 1.05626822 | 1.37E-06 |
| 'TRERF1' | -1.174497731 | 1.38E-06 |
| 'TTC30B' | -1.556948125 | 1.39E-06 |
| 'KCNJ13' | -4.209453366 | 1.43E-06 |
| 'GOLGA8R' | -3.273018494 | 1.45E-06 |
| 'IL1R1' | -1.551409941 | 1.50E-06 |
| 'TDRKH' | -1 | 1.50E-06 |
| 'AGAP9' | -1.092446249 | 1.73E-06 |
| 'PLEKHF1' | 1 | 1.76E-06 |
| 'RENBP' | -1.139551352 | 1.79E-06 |
| 'ZBTB7C' | -1 | 1.83E-06 |
| 'GOLGA6L3' | -6.108524457 | 1.83E-06 |
| 'TLR5' | -4.700439718 | 1.83E-06 |
| 'CHN2' | -1.126177106 | 1.87E-06 |
| 'KRT71' | 1.399747628 | 1.89E-06 |
| 'ZNF837' | -1.608046114 | 1.93E-06 |
| 'HLA-DMA' | -1.0061002 | 2.04E-06 |
| 'PCDHGA4' | -3.087462841 | 2.22E-06 |
| 'CAPS' | -2.08453351 | 2.42E-06 |
| 'SEMA3G' | -2.426264755 | 2.47E-06 |
| 'RGS6' | -3.192645078 | 2.57E-06 |
| 'C8orf44-SGK3' | -1.505235308 | 2.65E-06 |
| 'ZNF331' | -1.561878888 | 2.67E-06 |
| 'PDE4D' | -1.133855747 | 2.79E-06 |
| 'STARD9' | -1.152003093 | 2.85E-06 |
| 'DUSP13' | 2.454175893 | 2.86E-06 |
| 'LRMDA' | -1.334206425 | 2.89E-06 |
| 'COA5' | -1.003335713 | 3.03E-06 |
| 'EXOC3L4' | -2.852442812 | 3.54E-06 |
| 'NGEF' | -3.017921908 | 3.54E-06 |
| 'HABP2' | -5.209453366 | 3.67E-06 |
| 'GPR143' | 1.428172901 | 3.71E-06 |
| '112268394' | -1.537656786 | 3.74E-06 |
| 'FRAT1' | -1.241660755 | 3.79E-06 |
| 'GPD1' | -3.321928095 | 4.07E-06 |
| 'MYO1A' | -3.392317423 | 4.07E-06 |
| 'CCDC141' | -1.404390255 | 4.15E-06 |
| 'TNS2' | -1.163975735 | 4.25E-06 |
| 'MMP24-AS1-EDEM2' | -2.227805918 | 4.34E-06 |
| 'DNAJC18' | -1.009984089 | 4.51E-06 |
| 'TTLL3' | -1.725825037 | 4.78E-06 |
| 'TSSK4' | -1.253756592 | 4.78E-06 |
| 'RBP5' | -1.704544116 | 4.97E-06 |
| 'LOC100996709' | -1.304153393 | 5.17E-06 |
| 'PCDHGB2' | -2.039528364 | 5.27E-06 |
| 'TRPM6' | -2.736965594 | 5.27E-06 |
| 'TYMSOS' | -1.004843972 | 5.49E-06 |
| 'FGF18' | -1.664396968 | 5.51E-06 |
| 'CDRT1' | 1.0489096 | 5.76E-06 |
| 'TRIM6-TRIM34' | 4.95419631 | 5.91E-06 |
| 'CCL3' | 7.076815597 | 5.91E-06 |
| 'ARIH2OS' | -2.017921908 | 6.02E-06 |
| 'LOC100652901' | -1.14172811 | 6.32E-06 |
| 'SSTR1' | -1.534336428 | 6.65E-06 |
| 'TMEM107' | -1.014260693 | 6.72E-06 |
| 'PPARGC1A' | -2.357552005 | 7.32E-06 |
| 'TNFSF10' | -5.781359714 | 7.32E-06 |
| 'MXRA8' | -1.058893689 | 8.17E-06 |
| 'PDGFRL' | -1.020121596 | 8.20E-06 |
| 'NPIPA2' | -1.218046951 | 8.26E-06 |
| 'STX1B' | -1.765534746 | 8.54E-06 |
| 'VRTN' | -1.462626958 | 8.74E-06 |
| 'MKRN2OS' | -1.025648587 | 8.78E-06 |
| 'FAM122C' | -1.03170886 | 8.96E-06 |
| 'CMPK2' | -1.067744607 | 8.96E-06 |
| 'LDLRAD2' | -1.34191957 | 8.97E-06 |
| 'CYP2U1' | -1.371968777 | 9.01E-06 |
| 'C17orf82' | -1.385821711 | 9.02E-06 |
| 'LOC105374103' | -1.347923303 | 9.02E-06 |
| 'ZNF34' | -1.102361718 | 9.23E-06 |
| 'PRICKLE4' | -1.164535772 | 9.41E-06 |
| 'PLEKHS1' | -3.938599455 | 9.47E-06 |
| 'GP1BA' | -3.700439718 | 9.47E-06 |
| 'ERP27' | -1.479167837 | 1.05E-05 |
| 'SLC26A1' | -1.255944981 | 1.07E-05 |
| 'SLC28A3' | -1.410283969 | 1.08E-05 |
| 'CCL26' | 1.130321799 | 1.08E-05 |
| 'PCDH11X' | -2.887525271 | 1.09E-05 |
| 'COL5A1' | -2.169925001 | 1.14E-05 |
| 'PRF1' | -1.658963082 | 1.15E-05 |
| 'ATP6V1E2' | -1.566700294 | 1.19E-05 |
| 'NT5E' | -2.523561956 | 1.19E-05 |
| 'HTRA3' | 2.014950341 | 1.19E-05 |
| 'ALPI' | -2.022367813 | 1.19E-05 |
| 'SEMA6C' | -1.164744762 | 1.22E-05 |
| 'MAN1C1' | -2.432959407 | 1.23E-05 |
| 'BCAN' | -2.471305719 | 1.23E-05 |
| 'RETREG1' | -1.02774499 | 1.35E-05 |
| 'TGIF2-RAB5IF' | -6.459431619 | 1.46E-05 |
| 'LOC107984115' | -1.430634354 | 1.55E-05 |
| 'GRAPL' | -2.203091865 | 1.57E-05 |
| 'ARMCX4' | -1.237039197 | 1.58E-05 |
| 'EGR3' | -3.273018494 | 1.76E-05 |
| 'SPAG16' | -1.097297201 | 1.81E-05 |
| 'DENND2A' | -2 | 1.86E-05 |
| 'CAB39L' | -1.058102955 | 1.90E-05 |
| 'LOC101927509' | -4.523561956 | 1.95E-05 |
| 'HIVEP3' | -2.807354922 | 2.04E-05 |
| 'GALR2' | 1.658211483 | 2.13E-05 |
| 'RAD51B' | -1.060235107 | 2.22E-05 |
| 'YPEL2' | -1.201633861 | 2.23E-05 |
| 'ADSSL1' | -1.618909833 | 2.31E-05 |
| 'FSBP' | -1.700439718 | 2.38E-05 |
| 'IPP' | -1.019899557 | 2.39E-05 |
| 'LAMP3' | -1.050626073 | 2.43E-05 |
| 'TMEM136' | -2.807354922 | 2.45E-05 |
| 'RNF208' | -1.167599948 | 2.55E-05 |
| 'GPER1' | -1.051138849 | 2.67E-05 |
| 'PCOTH' | 1.001820436 | 2.74E-05 |
| 'SMIM5' | -1.321928095 | 2.74E-05 |
| 'SCHIP1' | -5.832890014 | 2.93E-05 |
| 'CD302' | -1.226275856 | 3.12E-05 |
| 'HNF1A' | -1.540568381 | 3.17E-05 |
| 'LRRC4B' | -2.099535674 | 3.26E-05 |
| 'TET1' | -1 | 3.26E-05 |
| 'PCDHGA6' | -2.765534746 | 3.27E-05 |
| 'LOC105370092' | -3.807354922 | 3.27E-05 |
| 'HHAT' | -1.278535499 | 3.31E-05 |
| 'EVL' | -2.215012891 | 3.31E-05 |
| 'LAMA1' | -2.662965013 | 3.31E-05 |
| 'CACNA1E' | -2.459431619 | 3.51E-05 |
| 'CD37' | -3.672425342 | 3.74E-05 |
| 'TLR3' | -1.516575526 | 3.78E-05 |
| 'PTPN13' | -1.137503524 | 3.81E-05 |
| 'TTC28' | -2.169925001 | 4.01E-05 |
| 'DNAH12' | -1.485426827 | 4.03E-05 |
| 'DPH3P1' | -1.334154397 | 4.03E-05 |
| 'CIDEB' | -1.160464672 | 4.04E-05 |
| 'ZBED2' | 2.765534746 | 4.06E-05 |
| 'CXCL10' | 1.359895945 | 4.32E-05 |
| 'CES3' | -1.100928909 | 4.35E-05 |
| 'WFDC3' | 2.055495113 | 4.39E-05 |
| 'LOC107986035' | -3.154577037 | 4.41E-05 |
| 'ERO1B' | -1.442518236 | 4.58E-05 |
| 'SLC4A4' | -1.552541023 | 4.72E-05 |
| 'RARRES2' | -1.485426827 | 4.72E-05 |
| 'LOXL1' | -1.127755547 | 4.88E-05 |
| 'PCSK4' | -1.376563351 | 4.88E-05 |
| 'LBH' | -1.105610188 | 5.16E-05 |
| 'CYP3A7' | -2.827819025 | 5.23E-05 |
| 'TTLL11' | 1.392317423 | 5.26E-05 |
| 'CGB8' | 1.345328527 | 5.39E-05 |
| 'KRTAP3-1' | 1.329587472 | 5.43E-05 |
| 'PALD1' | -1.920565533 | 5.60E-05 |
| 'SELENBP1' | -1.109624491 | 5.65E-05 |
| 'LOC101928120' | -1.679289883 | 5.66E-05 |
| 'GRAMD1B' | -1.479992941 | 5.81E-05 |
| 'TEX49' | -4.754887502 | 5.81E-05 |
| 'DNAH1' | -2.584962501 | 5.88E-05 |
| 'GAST' | 4.182203331 | 5.95E-05 |
| 'TRIM58' | -3.523561956 | 6.02E-05 |
| 'MMP11' | -3.350497247 | 6.02E-05 |
| 'AGAP5' | 1.697072867 | 6.09E-05 |
| 'TSPAN10' | -1.224560258 | 6.33E-05 |
| 'NSUN7' | -1.145979306 | 6.60E-05 |
| 'POLI' | -1.191298652 | 6.60E-05 |
| 'COL28A1' | -1.415037499 | 6.62E-05 |
| 'C1QL1' | -1.25334101 | 6.62E-05 |
| 'JDP2' | -1.461447964 | 6.70E-05 |
| 'FBXO2' | 1.224317298 | 6.94E-05 |
| 'B3GNT4' | -3.624490865 | 7.10E-05 |
| 'NPHP1' | -1.349584438 | 7.13E-05 |
| 'MAPK8IP2' | -1.788495895 | 7.13E-05 |
| '107986804' | 7.357552005 | 7.50E-05 |
| 'SPOCD1' | 1.271302022 | 7.64E-05 |
| 'GPSM1' | 1.258976189 | 7.93E-05 |
| 'LIPT2' | -1.089005006 | 8.07E-05 |
| 'LOC107985246' | -1.222392421 | 8.08E-05 |
| 'PXDN' | -1.662965013 | 8.68E-05 |
| 'HSPE1-MOB4' | 1.121990524 | 9.35E-05 |
| 'RASL11B' | 1.412125904 | 9.61E-05 |
| 'RHD' | -2.022367813 | 9.84E-05 |
| 'PLGLB1' | -2.502500341 | 9.88E-05 |
| 'FUZ' | -1.321928095 | 1.02E-04 |
| 'NLRP2' | -1.314510623 | 1.04E-04 |
| 'C5orf46' | 2.550197083 | 1.08E-04 |
| 'ELF5' | -3.321928095 | 1.11E-04 |
| 'SHROOM2' | -1.164386818 | 1.15E-04 |
| 'CDK5R2' | 1.435386145 | 1.19E-04 |
| 'SPN' | -1 | 1.21E-04 |
| 'SECTM1' | -1.46529227 | 1.24E-04 |
| 'FADS2' | -1.429987841 | 1.25E-04 |
| 'CACNA1D' | -1.450661409 | 1.26E-04 |
| 'ZNF169' | -1.568842835 | 1.26E-04 |
| 'FST' | -1.89077093 | 1.28E-04 |
| 'SCARF2' | -1.584962501 | 1.33E-04 |
| 'SERPING1' | -1.108706259 | 1.41E-04 |
| 'CEMP1' | 3.882643049 | 1.42E-04 |
| 'TMEM116' | -1.116917752 | 1.48E-04 |
| 'LETM2' | 1.131911676 | 1.49E-04 |
| 'TRAF1' | 2.95419631 | 1.51E-04 |
| 'NR1I3' | 2.770518154 | 1.51E-04 |
| 'MAP3K12' | -1.342392197 | 1.52E-04 |
| 'BORCS8-MEF2B' | -2.669851398 | 1.61E-04 |
| 'ME1' | 1.657112286 | 1.61E-04 |
| 'ITIH5' | -2.247927513 | 1.69E-04 |
| 'CEP85L' | -1.453717967 | 1.77E-04 |
| 'ESR1' | -1.494764692 | 1.79E-04 |
| 'IGF2BP1' | -1.716207034 | 1.80E-04 |
| 'NRIP2' | -1.818161677 | 1.80E-04 |
| 'NAV3' | 1.544320516 | 1.83E-04 |
| 'ATG9B' | 3.36923381 | 1.85E-04 |
| 'SIGLEC6' | -1.231325546 | 1.88E-04 |
| 'ADAMTSL2' | -1.222392421 | 1.99E-04 |
| 'HSD11B1L' | -1.263034406 | 1.99E-04 |
| 'ZNF829' | -1.862496476 | 2.04E-04 |
| 'LRRC36' | -3.40599236 | 2.04E-04 |
| 'C2orf81' | -1.746243408 | 2.13E-04 |
| 'ODF3B' | -1.172579455 | 2.16E-04 |
| 'RAET1G' | -1.093659107 | 2.21E-04 |
| 'BPIFB1' | -1.376854305 | 2.23E-04 |
| 'TMEM163' | -1.84502534 | 2.28E-04 |
| 'L1TD1' | -4.169925001 | 2.31E-04 |
| 'CSF3' | -5.614709844 | 2.31E-04 |
| 'SLAMF6' | -3.906890596 | 2.31E-04 |
| 'APOBEC3D' | -1.654004145 | 2.39E-04 |
| 'OSCP1' | -1.130396637 | 2.40E-04 |
| 'DND1' | -1.014548309 | 2.44E-04 |
| 'COL9A3' | -2.777607579 | 2.52E-04 |
| 'IGFBP7' | -4 | 2.57E-04 |
| 'INHBE' | -3.584962501 | 2.57E-04 |
| 'LOC653513' | -1.392317423 | 2.63E-04 |
| 'FAM167A' | -1.124328135 | 2.66E-04 |
| 'KRT34' | 5.426264755 | 2.67E-04 |
| 'SYNE4' | -2.023083613 | 2.83E-04 |
| 'PCDHB10' | -2.095157233 | 2.83E-04 |
| 'SLAMF9' | 1.95550054 | 3.01E-04 |
| 'APOLD1' | -1.459431619 | 3.19E-04 |
| 'DHRS12' | -1.249476301 | 3.19E-04 |
| 'SAMD11' | -1.836501268 | 3.23E-04 |
| 'LOC107985911' | 1.047048573 | 3.39E-04 |
| 'CLIP3' | -1.584962501 | 3.62E-04 |
| 'LOC107985532' | -3.169925001 | 3.72E-04 |
| 'C6orf226' | -1.27702487 | 3.77E-04 |
| 'ZC3H12D' | -1.691877705 | 3.84E-04 |
| 'C17orf113' | -1.700439718 | 3.84E-04 |
| 'LHX4' | -1.142019005 | 3.94E-04 |
| 'ZNF117' | -1.043068722 | 4.03E-04 |
| 'ACSM1' | -2.554588852 | 4.47E-04 |
| 'AQP1' | -2.772589504 | 4.47E-04 |
| 'PLCB1' | -1.023846742 | 4.47E-04 |
| 'CARMIL3' | -3 | 4.47E-04 |
| 'AKAP12' | -2.807354922 | 4.47E-04 |
| 'LMNTD2' | -1.151065974 | 4.51E-04 |
| 'SYT8' | -1.538866086 | 4.58E-04 |
| 'CDH12' | -4.247927513 | 4.60E-04 |
| 'C4A' | -3.584962501 | 4.60E-04 |
| 'CYP2D6' | -5.554588852 | 4.60E-04 |
| 'PCDHGC3' | -4.523561956 | 4.60E-04 |
| 'SV2A' | -4.459431619 | 4.60E-04 |
| 'LOC105379417' | -2.189824559 | 4.72E-04 |
| 'VWA5A' | -2.852442812 | 4.72E-04 |
| 'LTB4R2' | -1.189033824 | 4.82E-04 |
| 'LOC107987237' | -2.517848305 | 4.82E-04 |
| 'SMPX' | -2.531720479 | 4.82E-04 |
| 'TMEM249' | -2.273018494 | 4.88E-04 |
| 'KCNAB3' | -2.115477217 | 4.88E-04 |
| 'PROB1' | -2.357552005 | 4.88E-04 |
| 'ARHGAP4' | -2.357552005 | 4.88E-04 |
| 'PRXL2A' | -2.437405312 | 5.04E-04 |
| 'USP2' | -1.608809243 | 5.10E-04 |
| 'SLC2A4' | -1.142444265 | 5.12E-04 |
| 'GDPD1' | -1.236067358 | 5.37E-04 |
| 'LOC112268342' | -1.351472371 | 5.46E-04 |
| 'BBOF1' | -1.455194626 | 5.46E-04 |
| 'CFI' | -1.777607579 | 5.81E-04 |
| 'COLCA2' | -1.94596016 | 5.81E-04 |
| 'SNRPN' | -1.690895945 | 5.92E-04 |
| 'CBX7' | -1.046293652 | 5.96E-04 |
| 'DGLUCY' | -1.94753258 | 6.52E-04 |
| 'ITGA10' | -1.765534746 | 6.52E-04 |
| 'PCDHB12' | -1.854149134 | 6.52E-04 |
| 'FBXL13' | -1.754887502 | 6.52E-04 |
| 'PPP5D1' | -2.283792966 | 6.52E-04 |
| '112267923' | -3.129283017 | 6.73E-04 |
| 'HIST1H3G' | -3.163230349 | 6.73E-04 |
| 'RGPD4' | -1.700439718 | 6.90E-04 |
| 'ABHD14A-ACY1' | 1.056034035 | 7.01E-04 |
| 'PDE6A' | -1.165059246 | 7.20E-04 |
| 'TSPOAP1' | -2 | 7.20E-04 |
| 'CDIP1' | -1.925999419 | 7.20E-04 |
| 'BCL2L14' | -1.598637438 | 7.69E-04 |
| 'KPNA7' | 1.762960803 | 7.73E-04 |
| 'PLEKHD1' | -2 | 7.80E-04 |
| 'P2RY2' | -1.765534746 | 7.80E-04 |
| 'AFAP1L2' | -2.64385619 | 7.83E-04 |
| 'RD3' | -2.584962501 | 7.83E-04 |
| 'RUNX1T1' | -2.115477217 | 7.84E-04 |
| 'SSC4D' | -1.162938571 | 7.98E-04 |
| 'LTC4S' | -1.768674454 | 7.99E-04 |
| 'SLC43A1' | -1.38827059 | 8.17E-04 |
| 'ADAMTS6' | -1.30256277 | 8.18E-04 |
| 'LOC100505549' | -2.215012891 | 8.19E-04 |
| '112267922' | -2.078002512 | 8.26E-04 |
| 'ST3GAL3' | -1.234465254 | 8.26E-04 |
| 'LOC107986211' | -1.289506617 | 8.26E-04 |
| 'CCDC74A' | -1.311201688 | 8.40E-04 |
| 'HCFC2' | 1.180572246 | 8.41E-04 |
| 'PRDM16' | -3.459431619 | 9.08E-04 |
| 'TREM1' | -3.222392421 | 9.08E-04 |
| 'SERPINA5' | -4.044394119 | 9.09E-04 |
| 'CTTNBP2' | -3.700439718 | 9.09E-04 |
| 'ADCY2' | -3.169925001 | 9.09E-04 |
| 'PRR5-ARHGAP8' | -4.95419631 | 9.09E-04 |
| 'LOC107985728' | -4.087462841 | 9.09E-04 |
| 'ARNT2' | -3.169925001 | 9.10E-04 |
| 'KRT36' | -5.209453366 | 9.10E-04 |
| 'USP17L15' | -5.209453366 | 9.10E-04 |
| 'SOX5' | -3.700439718 | 9.10E-04 |
| 'HOXA3' | -4.169925001 | 9.10E-04 |
| 'ZIC2' | -4.321928095 | 9.10E-04 |
| 'SYT2' | -3.459431619 | 9.10E-04 |
| 'KRTAP2-3' | 1.062378785 | 9.29E-04 |
| 'FGF1' | 3 | 9.58E-04 |
| 'TBC1D3H' | -1.211504105 | 9.75E-04 |
| 'NUTM2A' | -1.022367813 | 9.97E-04 |

Supplementary Table 10. Identification of the AQP5 protein complex by mass spectrometry

| Protein_ID | Description | iBAQ |
| --- | --- | --- |
| NP_001642.1 | aquaporin-5 | 30556485.39 |
| NP_006112.3 | keratin, type II cytoskeletal 1 | 20555871.14 |
| NP_000412.4 | keratin, type I cytoskeletal 10 isoform 1 | 14504326 |
| NP_000217.2 | keratin, type I cytoskeletal 9 | 7186341.324 |
| NP_000414.2 | keratin, type II cytoskeletal 2 epidermal | 6644792.324 |
| NP_878912.1 | dehydrogenase/reductase SDR family member 2, mitochondrial isoform 1 | 2427465.631 |
| NP_000468.1 | albumin preproprotein | 2241150.119 |
| NP_000415.2 | keratin, type II cytoskeletal 5 | 1272116.766 |
| NP_001029249.1 | histone H4 | 1107569.379 |
| NP_000962.2 | 60S ribosomal protein L7 isoform 1 | 1094066.268 |
| NP_000509.1 | hemoglobin subunit beta | 876282.3075 |
| NP_001287783.1 | dermcidin isoform 2 preproprotein | 835260.3967 |
| NP_077017.1 | UPF0184 protein C9orf16 | 804233.7139 |
| NP_000517.3 | keratin, type I cytoskeletal 14 | 690814.1854 |
| NP_000508.1 | hemoglobin subunit alpha >NP_000549.1 hemoglobin subunit alpha | 612912.7387 |
| NP_003840.2 | succinate--CoA ligase [ADP/GDP-forming] subunit alpha, mitochondrial precursor | 470086.5928 |
| NP_000972.1 | 60S ribosomal protein L19 isoform 1 | 453735.5341 |
| NP_821133.1 | tubulin beta chain isoform b | 401189.7461 |
| NP_000286.3 | alpha-1-antitrypsin | 375355.2544 |
| NP_005338.1 | endoplasmic reticulum chaperone BiP precursor | 357967.2088 |
| NP_001243222.1 | keratin, type II cytoskeletal 8 isoform 2 | 350740.511 |
| NP_001002858.1 | annexin A2 isoform 1 | 339591.4009 |
| NP_001092.1 | actin, cytoplasmic 1 | 338681.626 |
| NP_001121188.1 | electron transfer flavoprotein subunit alpha, mitochondrial isoform b | 306501.084 |
| NP_004951.1 | RNA-binding protein FUS isoform 1 | 297619.7397 |
| NP_001393.1 | elongation factor 1-alpha 1 | 297120.1652 |
| NP_001924.2 | dihydrolipoyllysine-residue succinyltransferase component of 2-oxoglutarate dehydrogenase complex, mitochondrial isoform 1 precursor | 294834.7986 |
| NP_000413.1 | keratin, type I cytoskeletal 17 | 292422.8025 |
| NP_001036041.2 | ribosome-binding protein 1 isoform 2 | 286228.5012 |
| NP_004125.3 | stress-70 protein, mitochondrial precursor | 264485.3401 |
| NP_005309.1 | histone H1.0 | 262142.8516 |
| NP_003970.1 | retinoic acid-induced protein 3 | 230736.4437 |
| XP_016881278.1 | ATP synthase subunit alpha, mitochondrial isoform X1 | 229740.7137 |
| NP_005548.2 | keratin, type I cytoskeletal 16 | 227430.1758 |
| NP_002128.1 | heterogeneous nuclear ribonucleoproteins A2/B1 isoform A2 | 224118.4244 |
| NP_254280.1 | histone H2A type 3 | 223960.8966 |
| NP_001317178.1 | heterogeneous nuclear ribonucleoprotein A3 isoform a | 196893.9322 |
| NP_000030.1 | apolipoprotein A-I isoform 1 preproprotein | 189805.3449 |
| NP_005545.1 | keratin, type II cytoskeletal 6A | 185708.8462 |
| NP_006588.1 | heat shock cognate 71 kDa protein isoform 1 | 179289.6213 |
| XP_016885614.1 | uncharacterized protein LOC102723930 | 179048.0926 |
| NP_001231867.1 | thioredoxin isoform 2 | 161359.3372 |
| NP_001258901.1 | heat shock protein HSP 90-beta isoform c | 155894.3193 |
| NP_001369692.1 | fibrinogen beta chain isoform 7 preproprotein | 148015.2217 |
| NP_005204.1 | cystatin-A | 132892.5525 |
| NP_001243728.1 | glyceraldehyde-3-phosphate dehydrogenase isoform 2 | 124902.5581 |
| NP_005773.3 | THO complex subunit 4 | 122407.0162 |
| NP_000968.2 | 60S ribosomal protein L13 isoform 1 | 120834.9667 |
| NP_001743.1 | catalase | 115338.6668 |
| NP_000215.1 | keratin, type I cytoskeletal 18 | 113739.8275 |
| NP_005546.2 | keratin, type II cytoskeletal 6B | 104216.307 |
| NP_003371.2 | vimentin | 102375.5461 |
| NP_003132.2 | E3 ubiquitin-protein ligase TRIM21 | 101302.1283 |
| NP_001419.1 | alpha-enolase isoform 1 | 99857.86709 |
| NP_068656.2 | fibrinogen gamma chain isoform gamma-B precursor | 99473.67639 |
| NP_005134.1 | haptoglobin isoform 1 preproprotein | 96008.96062 |
| NP_001070911.1 | heterogeneous nuclear ribonucleoproteins C1/C2 isoform b | 95100.42987 |
| NP_064583.2 | coiled-coil domain-containing protein 47 precursor | 93647.60662 |
| NP_002127.1 | heterogeneous nuclear ribonucleoprotein A1 isoform a | 92436.1569 |
| NP_005150.1 | actin, alpha cardiac muscle 1 | 92015.68755 |
| NP_006003.1 | ATP-dependent Clp protease proteolytic subunit, mitochondrial precursor | 91179.55477 |
| NP_001257419.1 | 60S ribosomal protein L18 isoform 2 | 89605.30117 |
| NP_001435.1 | fatty acid-binding protein 5 | 89356.95872 |
| NP_001193943.1 | serpin H1 precursor | 84838.50409 |
| NP_001351170.1 | heterogeneous nuclear ribonucleoprotein H isoform f | 84508.56682 |
| NP_006796.1 | heterogeneous nuclear ribonucleoprotein A0 | 82451.59649 |
| XP_011523095.1 | keratin, type I cuticular Ha4 isoform X1 | 81117.2185 |
| NP_001634.1 | apolipoprotein A-II preproprotein | 78874.46661 |
| NP_002147.2 | 60 kDa heat shock protein, mitochondrial | 78491.52787 |
| NP_002267.2 | keratin, type I cytoskeletal 19 | 76244.66368 |
| NP_002268.2 | keratin, type I cuticular Ha1 | 72588.37744 |
| NP_000998.1 | 40S ribosomal protein S4, X isoform | 71231.47774 |
| NP_005304.3 | protein disulfide-isomerase A3 precursor | 70808.57391 |
| NP_077739.1 | desmocollin-1 isoform Dsc1a preproprotein | 68979.4483 |
| NP_001020241.1 | 40S ribosomal protein S14 | 68337.20998 |
| NP_002270.1 | keratin, type I cuticular Ha3-II | 66004.68277 |
| NP_001143.2 | ADP/ATP translocase 2 | 61973.02282 |
| NP_000975.2 | 60S ribosomal protein L23a | 61325.36007 |
| NP_001348.2 | ATP-dependent RNA helicase A | 60981.16328 |
| NP_000230.1 | lysozyme C precursor | 60324.13579 |
| NP_001352289.1 | elongation factor Tu, mitochondrial isoform 2 precursor | 59524.37379 |
| NP_005372.2 | nucleolin | 59026.87975 |
| NP_001933.2 | desmoglein-1 preproprotein | 57995.77237 |
| NP_001677.2 | ATP synthase subunit beta, mitochondrial precursor | 57600.31634 |
| NP_000959.2 | 60S ribosomal protein L4 | 55548.90697 |
| NP_001254629.1 | prohibitin-2 isoform 3 | 54400.28729 |
| NP_068657.1 | fibrinogen alpha chain isoform alpha precursor | 54112.18349 |
| NP_000981.1 | 60S ribosomal protein L27a | 53247.79962 |
| NP_001054.2 | serotransferrin isoform 1 precursor | 50024.18956 |
| NP_072045.1 | 40S ribosomal protein S18 | 49735.60736 |
| NP_001269554.1 | lamin isoform C | 47077.49851 |
| NP_001294857.1 | serpin B12 isoform 1 | 46556.13414 |
| XP_011514996.1 | putative maltase-glucoamylase-like protein FLJ16351 isoform X3 | 46050.05446 |
| NP_001306127.1 | protein S100-A8 isoform c | 45933.56807 |
| NP_005057.1 | splicing factor, proline- and glutamine-rich | 45273.25272 |
| NP_000598.2 | alpha-1-acid glycoprotein 1 precursor | 43343.35426 |
| NP_003290.1 | endoplasmin precursor | 42867.37187 |
| NP_000911.2 | pyruvate carboxylase, mitochondrial precursor | 39796.48328 |
| XP_011519466.1 | ATP-dependent Clp protease ATP-binding subunit clpX-like, mitochondrial isoform X1 | 38853.30577 |
| NP_001339702.1 | junction plakoglobin | 38657.55443 |
| NP_009140.1 | 60S ribosomal protein L35 | 37813.14592 |
| NP_001019820.1 | calnexin isoform d precursor | 36536.90797 |
| NP_000997.1 | 40S ribosomal protein S3a isoform 1 | 36351.37951 |
| NP_001356414.1 | protein LSM12 homolog isoform 2 | 34934.62652 |
| NP_001176.1 | zinc-alpha-2-glycoprotein precursor | 34710.90863 |
| NP_000963.1 | 60S ribosomal protein L7a | 34603.41286 |
| NP_004406.2 | desmoplakin isoform I | 32998.30554 |
| NP_001341935.1 | nucleophosmin isoform 1 >NP_002511.1 nucleophosmin isoform 1 | 31814.97546 |
| NP_149034.2 | keratin, type II cuticular Hb4 | 30677.58783 |
| NP_001001.2 | 40S ribosomal protein S6 | 30528.56125 |
| NP_001017963.2 | heat shock protein HSP 90-alpha isoform 1 | 30345.48109 |
| NP_002272.2 | keratin, type II cuticular Hb1 | 28771.91723 |
| NP_778253.2 | keratin, type II cytoskeletal 1b | 28454.5049 |
| NP_001011709.2 | pancreatic lipase-related protein 3 precursor | 28422.65576 |
| XP_016875261.1 | GTP-binding nuclear protein Ran isoform X1 | 28025.80657 |
| NP_053733.2 | RNA-binding protein EWS isoform 1 | 27940.53623 |
| NP_005336.3 | heat shock 70 kDa protein 1A | 27836.2359 |
| NP_001013.1 | 40S ribosomal protein S19 isoform 1 | 26967.37149 |
| NP_001255.4 | corneodesmosin precursor | 26787.10878 |
| NP_061848.2 | telomeric repeat-binding factor 2-interacting protein 1 | 26744.91599 |
| XP_011536312.1 | keratin, type II cytoskeletal 78 isoform X1 | 25919.50417 |
| NP_001531.1 | heat shock protein beta-1 | 24891.90632 |
| XP_011511897.1 | transcriptional adapter 2-beta isoform X1 | 24396.44906 |
| NP_001231367.1 | arginase-1 isoform 1 | 23579.20546 |
| NP_003236.3 | protein-glutamine gamma-glutamyltransferase E | 22774.36046 |
| NP_001365399.1 | serine/threonine-protein kinase B-raf isoform 8 | 22700.41186 |
| NP_001306006.1 | high mobility group protein HMG-I/HMG-Y isoform b | 22500.34977 |
| NP_570843.2 | leucine-rich repeat-containing protein 15 isoform b precursor | 21475.53336 |
| NP_001009931.1 | hornerin | 21335.20386 |
| NP_001349460.1 | creatine kinase B-type isoform 2 | 21246.4729 |
| NP_114032.2 | heterogeneous nuclear ribonucleoprotein U isoform a | 20934.68926 |
| NP_001280202.1 | methylcrotonoyl-CoA carboxylase subunit alpha, mitochondrial isoform 2 | 20823.39428 |
| NP_001035810.1 | glucose-6-phosphate 1-dehydrogenase isoform b | 20128.34655 |
| NP_005557.1 | L-lactate dehydrogenase A chain isoform 1 | 20107.55518 |
| NP_005312.1 | histone H1.4 | 18594.19426 |
| NP_001014402.1 | katanin p60 ATPase-containing subunit A-like 1 | 16892.85684 |
| NP_006079.1 | tubulin beta-4B chain | 16725.33626 |
| NP_036246.1 | caspase-14 precursor | 15933.79777 |
| NP_002941.1 | dolichyl-diphosphooligosaccharide--protein glycosyltransferase subunit 1 precursor | 15830.18115 |
| NP_004930.1 | ATP-dependent RNA helicase DDX1 | 15445.39597 |
| NP_000599.1 | alpha-1-acid glycoprotein 2 precursor | 15140.23406 |
| NP_001026854.1 | serine/arginine-rich splicing factor 7 isoform 1 | 15063.29927 |
| NP_116093.1 | tubulin alpha-1C chain isoform c | 14120.47173 |
| NP_000377.1 | bleomycin hydrolase | 13995.1543 |
| XP_016868070.1 | zonadhesin isoform X1 | 13600.11023 |
| NP_000933.1 | peptidyl-prolyl cis-trans isomerase B precursor | 13452.45112 |
| NP_006818.3 | transmembrane emp24 domain-containing protein 10 precursor | 13310.42722 |
| NP_000350.1 | protein-glutamine gamma-glutamyltransferase K | 12488.86509 |
| NP_004896.1 | peroxiredoxin-6 | 11970.668 |
| XP_016865666.1 | heterogeneous nuclear ribonucleoprotein Q isoform X2 | 11159.11236 |
| NP_002130.2 | RNA-binding motif protein, X chromosome isoform 1 | 10943.72482 |
| NP_001171597.1 | immunoglobulin lambda-like polypeptide 5 isoform 1 | 10933.00114 |
| NP_001307526.1 | probable ATP-dependent RNA helicase DDX5 isoform b | 10280.36279 |
| NP_001306115.1 | hepatoma-derived growth factor isoform d | 10209.81587 |
| XP_016885267.1 | ubiquitin-like modifier-activating enzyme 1 isoform X2 | 9693.466497 |
| NP_002274.1 | keratin, type II cuticular Hb5 isoform 1 | 9564.586726 |
| NP_000055.2 | complement C3 preproprotein | 9455.221212 |
| NP_877963.1 | 1-phosphatidylinositol 4,5-bisphosphate phosphodiesterase gamma-1 isoform b | 8765.966987 |
| NP_002464.1 | myosin-9 | 8687.705446 |
| NP_008850.1 | serpin B3 | 8668.029106 |
| NP_000977.1 | 60S ribosomal protein L24 | 8613.178767 |
| NP_000993.1 | 60S acidic ribosomal protein P0 | 8478.889377 |
| NP_066964.1 | X-ray repair cross-complementing protein 5 | 8220.567306 |
| NP_001317121.1 | nuclear pore complex protein Nup107 isoform 2 | 7968.309689 |
| NP_066289.3 | polyubiquitin-C | 7878.96518 |
| XP_011534757.1 | serpin A12 isoform X2 | 7641.650091 |
| NP_000961.2 | 60S ribosomal protein L6 isoform 1 | 7620.979141 |
| NP_001189360.1 | peroxiredoxin-1 | 7335.871472 |
| NP_001273149.1 | aspartate aminotransferase, mitochondrial isoform 2 precursor | 7289.146016 |
| NP_001207423.1 | glycine N-acyltransferase-like protein 1 isoform 2 | 7086.419488 |
| NP_001627.2 | ADP/ATP translocase 3 | 6938.102765 |
| XP_024307077.1 | transcription intermediary factor 1-beta isoform X1 | 6759.366318 |
| NP_001596.2 | alanine--tRNA ligase, cytoplasmic | 6569.630128 |
| NP_001189399.1 | RPS10-NUDT3 protein | 6242.990953 |
| NP_001447.2 | filamin-A isoform 1 | 5874.968408 |
| NP_006176.2 | nuclear mitotic apparatus protein 1 isoform 1 | 5839.073173 |
| NP_006784.1 | thioredoxin-dependent peroxide reductase, mitochondrial isoform a precursor | 5366.540022 |
| NP_001020402.1 | keratinocyte proline-rich protein | 5332.996416 |
| XP_006713564.1 | ceruloplasmin isoform X5 | 5319.97145 |
| NP_000971.1 | 60S ribosomal protein L18a | 5212.977375 |
| NP_001029025.1 | 60S ribosomal protein L3 isoform b | 5178.539044 |
| NP_001609.2 | poly [ADP-ribose] polymerase 1 | 5121.485894 |
| XP_016882223.1 | protein arginine N-methyltransferase 1 isoform X1 | 5106.967994 |
| NP_689714.2 | pyrin and HIN domain-containing protein 1 isoform alpha 1 | 4976.511554 |
| NP_000031.1 | apolipoprotein C-III precursor | 4488.712732 |
| NP_001030168.1 | 60S ribosomal protein L14 | 4356.229814 |
| NP_001427.2 | rRNA 2'-O-methyltransferase fibrillarin | 4212.592043 |
| XP_016880661.1 | SRC kinase signaling inhibitor 1 isoform X4 | 4071.812249 |
| XP_011512637.1 | leukocyte elastase inhibitor isoform X2 | 3897.61494 |
| NP_001001414.1 | F-box only protein 50 | 3572.291981 |
| NP_001091674.1 | heterogeneous nuclear ribonucleoprotein F | 3565.861054 |
| NP_000290.2 | plakophilin-1 isoform 1b | 3432.886261 |
| NP_001129171.1 | 14-3-3 protein zeta/delta | 3359.894157 |
| NP_001350789.1 | 40S ribosomal protein S16 isoform 3 | 3274.213508 |
| NP_001003810.1 | heterogeneous nuclear ribonucleoprotein D0 isoform d | 3092.978664 |
| NP_001338962.1 | DAZ-associated protein 1 isoform c | 3047.045796 |
| NP_001269582.1 | stress-induced-phosphoprotein 1 isoform c | 2997.994593 |
| NP_001074961.1 | keratin, type II cytoskeletal 80 isoform K80.1 | 2935.201861 |
| NP_004484.1 | 3-hydroxyacyl-CoA dehydrogenase type-2 isoform 1 | 2899.58585 |
| NP_060090.2 | interleukin enhancer-binding factor 3 isoform d | 2722.153104 |
| NP_001014364.1 | filaggrin-2 | 2682.029718 |
| NP_066953.1 | peptidyl-prolyl cis-trans isomerase A isoform 1 | 2608.520718 |
| NP_001297244.1 | tubulin beta-2A chain isoform 2 | 2604.792741 |
| NP_003762.1 | keratin, type I cuticular Ha6 | 2600.374078 |
| NP_005653.3 | voltage-dependent anion-selective channel protein 3 isoform 1 | 2598.755873 |
| NP_005264.2 | guanine nucleotide-binding protein G(I)/G(S)/G(T) subunit beta-2 | 2596.730133 |
| NP_001193726.1 | pyruvate kinase PKM isoform d | 2556.83736 |
| NP_000996.2 | 40S ribosomal protein S3 isoform 1 | 2096.233714 |
| NP_000909.2 | protein disulfide-isomerase precursor | 2045.938637 |
| NP_001309108.1 | contactin-associated protein-like 4 isoform 4 precursor | 1948.634178 |
| NP_001335874.1 | ATP-dependent translocase ABCB1 isoform 1 | 1923.109765 |
| NP_004334.1 | calreticulin precursor | 1868.608591 |
| XP_024305886.1 | hemicentin-1 isoform X3 | 1713.491427 |
| NP_112480.2 | heterogeneous nuclear ribonucleoprotein M isoform b | 1686.454168 |
| NP_006422.1 | T-complex protein 1 subunit beta isoform 1 | 1623.032322 |
| NP_079197.3 | protein POF1B isoform 1 | 1578.243843 |
| NP_005207.3 | dolichyl-diphosphooligosaccharide--protein glycosyltransferase 48 kDa subunit precursor | 1341.769616 |
| XP_005251038.1 | plectin isoform X7 | 1334.617935 |
| NP_476429.2 | keratin, type II cytoskeletal 3 | 1265.313947 |
| XP_016884445.1 | clathrin heavy chain 2 isoform X4 | 1139.954108 |
| NP_036565.2 | splicing factor 3B subunit 1 isoform 1 | 1017.97529 |
| NP_006182.2 | proliferation-associated protein 2G4 | 990.6232288 |
| NP_001129676.1 | protein RCC2 | 798.8861281 |
| NP_002435.1 | moesin | 791.8120659 |
| NP_001263382.1 | importin subunit beta-1 isoform 2 | 726.4282608 |
| NP_006187.2 | poly(rC)-binding protein 1 | 711.2351778 |
| NP_001275905.1 | X-ray repair cross-complementing protein 6 isoform 1 | 625.6266603 |
| NP_112598.3 | epiplakin | 600.1279145 |
| NP_001182461.1 | spectrin alpha chain, non-erythrocytic 1 isoform 3 | 535.4470122 |
| XP_005259654.1 | polypyrimidine tract-binding protein 1 isoform X1 | 466.9709662 |
| NP_004519.1 | microsomal glutathione S-transferase 3 | 438.6011089 |
| NP_001287843.1 | dnaJ homolog subfamily B member 1 isoform 2 | 411.3280344 |
| NP_001316019.1 | ephrin type-A receptor 2 isoform 2 | 352.084128 |
| NP_000080.2 | collagen alpha-2(I) chain precursor | 267.0757576 |
| NP_001257328.1 | tubulin alpha-1A chain isoform 1 | 260.8171457 |
| NP_001333374.1 | neuroblast differentiation-associated protein AHNAK isoform 1 | 245.4679407 |
| NP_006436.3 | pre-mRNA-processing-splicing factor 8 | 232.3959869 |
| NP_001310353.1 | RNA-binding protein 39 isoform g | 153.3499452 |
| NP_009123.1 | FACT complex subunit SPT16 | 49.35108641 |
| XP_024307775.1 | helicase with zinc finger domain 2 isoform X2 | 23.52758793 |
| NP_853631.1 | keratin-associated protein 13-4 | 0 |
| NP_003283.2 | nucleoprotein TPR | 0 |
| NP_001158255.1 | 2-oxoisovalerate dehydrogenase subunit alpha, mitochondrial isoform 2 precursor | 0 |
| NP_149972.1 | L-lactate dehydrogenase A-like 6B | 0 |
| NP_001018077.1 | plasminogen activator inhibitor 1 RNA-binding protein isoform 1 | 0 |
| NP_003365.1 | voltage-dependent anion-selective channel protein 1 | 0 |
| NP_006089.1 | receptor of activated protein C kinase 1 | 0 |
| NP_005879.1 | phosphate carrier protein, mitochondrial isoform a precursor | 0 |
| NP_001055.1 | transketolase isoform 1 | 0 |
| NP_001010942.1 | ras-related protein Rap-1b isoform 1 precursor | 0 |
| NP_000079.2 | collagen alpha-1(I) chain preproprotein | 0 |
| NP_001342150.1 | putative tubulin-like protein alpha-4B | 0 |
| NP_001395.1 | elongation factor 1-gamma | 0 |
| NP_001171752.1 | voltage-dependent anion-selective channel protein 2 isoform 2 | 0 |
| NP_001305116.1 | heterogeneous nuclear ribonucleoprotein K isoform d | 0 |
| NP_056016.1 | ribosome biogenesis protein BOP1 | 0 |
| NP_060530.3 | isoleucine--tRNA ligase, mitochondrial precursor | 0 |
| NP_057018.1 | nucleolar protein 58 | 0 |
| NP_001121089.1 | fructose-bisphosphate aldolase A isoform 1 | 0 |
| NP_853630.2 | keratin-associated protein 13-1 | 0 |
| NP_001132913.1 | B-cell receptor-associated protein 31 isoform b | 0 |
| XP_024309179.1 | nuclear pore membrane glycoprotein 210 isoform X1 | 0 |
| NP_001305904.1 | 2-oxoisovalerate dehydrogenase subunit beta, mitochondrial isoform 2 | 0 |
| NP_001120700.1 | chromobox protein homolog 1 | 0 |
| XP_006719137.1 | L-lactate dehydrogenase B chain isoform X1 | 0 |
| NP_000989.1 | 60S ribosomal protein L37a | 0 |
| NP_001123631.1 | storkhead-box protein 1 isoform b | 0 |
| NP_001307540.1 | importin subunit alpha-1 | 0 |
| NP_001243439.1 | single-stranded DNA-binding protein, mitochondrial precursor | 0 |
| NP_001349.2 | pre-mRNA-splicing factor ATP-dependent RNA helicase DHX15 | 0 |
| NP_001365185.1 | small nuclear ribonucleoprotein-associated protein N isoform c | 0 |
| XP_011539528.1 | D-3-phosphoglycerate dehydrogenase isoform X1 | 0 |
| NP_001076.2 | alpha-1-antichymotrypsin precursor | 0 |
| NP_055121.1 | RNA-splicing ligase RtcB homolog | 0 |
| NP_001152759.1 | triosephosphate isomerase isoform 2 | 0 |
| NP_003519.1 | histone H2B type 2-E | 0 |
| NP_001281074.1 | lupus La protein | 0 |
| NP_005800.3 | peroxiredoxin-2 | 0 |
| NP_005909.2 | malate dehydrogenase, mitochondrial isoform 1 precursor | 0 |
| NP_001186911.1 | NADH-ubiquinone oxidoreductase 75 kDa subunit, mitochondrial isoform 3 | 0 |
| NP_001268425.1 | prohibitin isoform 1 | 0 |
| NP_001137232.1 | eukaryotic translation initiation factor 5A-1 isoform A | 0 |
| NP_001016.1 | 40S ribosomal protein S23 | 0 |
| XP_011512592.1 | forkhead box protein P4 isoform X2 | 0 |
| NP_001122384.1 | poly(rC)-binding protein 2 isoform e | 0 |
| XP_005270602.1 | lipoamide acyltransferase component of branched-chain alpha-keto acid dehydrogenase complex, mitochondrial isoform X1 | 0 |
| XP_016877210.1 | alpha-actinin-1 isoform X2 | 0 |
| NP_001900.1 | cathepsin D preproprotein | 0 |
| XP_005251560.1 | WD40 repeat-containing protein SMU1 isoform X1 | 0 |
| NP_853652.1 | keratin-associated protein 13-2 | 0 |
| NP_000282.1 | phosphoglycerate kinase 1 | 0 |
| NP_001316363.2 | nuclear pore complex protein Nup205 isoform 2 | 0 |
| NP_001958.2 | eukaryotic initiation factor 4A-II | 0 |
| NP_001243839.1 | nucleolar RNA helicase 2 isoform 2 | 0 |
| NP_002937.1 | replication protein A 32 kDa subunit isoform 1 | 0 |
| NP_001332797.1 | translation factor GUF1, mitochondrial isoform 3 | 0 |
| NP_001524.2 | heterogeneous nuclear ribonucleoprotein L isoform a | 0 |
| XP_005261192.1 | ATP-dependent 6-phosphofructokinase, liver type isoform X3 | 0 |
| NP_037460.2 | pyrroline-5-carboxylate reductase 2 isoform 1 | 0 |
