# Supplementary material for: AQP5 complements LGR5 to determine the fates of gastric cancer stem cells through regulating ULK1 ubiquitination
Source: J Exp Clin Cancer Res. 2022 Nov 14;41:322. doi: 10.1186/s13046-022-02532-w (PMC9661769; doi:10.1186/s13046-022-02532-w)
Supplement: Supplementary file 3 — Additional file 3. [file 13046_2022_2532_MOESM3_ESM.docx]

**Supplementary Methods**

**Single-cell suspension preparation**

Fresh gastric cancer and matched adjacent tissues were collected. Under sterile conditions, the cells were washed twice with pre-cooled RPMI 1640+0.04% BSA medium. The tissues were cut into small pieces of about 0.5 mm^3^ with surgical scissors, placed into freshly prepared enzymatic hydrolysate (enzyme solution composition, concentration and brand), digested in a constant temperature of 37 ℃ for 60 minutes, and mixed every 10 minutes. BD 40 was used to digest the cell suspension. A cell sieve was used to filter the tissues 2 times, and the samples were centrifuged at 300 g at 4 °C for 5 min. After resuspending the precipitates with an appropriate amount of medium, an equal volume of erythrocyte lysis buffer (MACS, Catalog No. 130-094-183) was added. After mixing, the cells were incubated at 4 ℃ for 10 minutes and centrifuged at 300 × g for 5 minutes, and the supernatant was discarded. The pellet was washed once with the culture medium and centrifuged for 5 min at 300 g. The supernatant was discarded, 100 μl of the cell precipitate suspension was added to culture medium, and the cell concentration and viability were calculated by a Luna cell counter.

**On-board, library construction, sequencing**

On-board and library construction were performed according to the 10× Genomics Chromium Next GEM Single Cell 3ʹ Reagent Kits v3.1 (Cat. No. 1000268) following the manufacturer’s instructions. The constructed library was used for high-throughput sequencing on the Illumina Nova 6000 PE150 platform.

**Sequencing data quality control and gene quantification**

Cellranger preliminary quality control and the Seurat software package were used for further quality control of the data. Low-quality cells were filtered based on the distribution of nUMI, nGene and percent.mito (The number of genes per cell was retained, and UMI within the median +/- 2 times the absolute median difference and the proportion of mitochondrial transcripts were determined. Cells below 20% were considered high-quality cells, and doublet cells were removed using DoubletFinder software).

**Dimensionality reduction and cluster analysis**

The findvariablegenes function in the Seurat package was used to screen highly variable genes (HVGS). Principal component analysis (PCA) dimensionality reduction was performed by using the expression profile of hypervariable genes. The results were visualized in two-dimensional space through umap (nonlinear dimensionality reduction).

**Cell culture and transfection**

The AGS and HGC-27 gastric cancer (GC) cell lines and the HEK 293 T cell line were purchased from Procell. The cells were grown in DF-12 medium (Gibco) supplemented with 10% fetal bovine serum (Gibco). The cells were incubated at 37 °C in a humidified atmosphere with 5% CO_2_.

The cells were transfected with pSLenti-AQP5 and control vector according to the manufacturer's instructions. Stably transfected cell lines were obtained after selection with 1.5 μg/mL puroMycin (Gibco) for 6 days. The AQP5/LGR5 shRNA sequences of the control and target-specific genes are listed in Supplementary Table 4. Transfection of the WT-ULK1, WT-TRIM2, WT-Ubiquitin, Ubiquitin-K63R plasmids and control empty vector was performed using Lipofectamine 3000 reagent (Invitrogen) according to the manufacturer's instructions. The transfection of TRIM21, ATG7, or UBB/UBC siRNAs and negative control siRNAs (NC) were performed using Lipofectamine 3000 reagent (Invitrogen) according to the manufacturer's instructions. The corresponding sequences are shown in Supplementary Table 4. The cells were harvested 48–72 h post-transfection for various assays.

**Tissue Single-Cell Suspension Preparation**

First, the fresh GC tissues were washed with PBS and the clot and necrotic tissues were excised.Then, the enzyme mixture was prepared according to the manufacturer's requirements (Miltenyi Biotec). The tissues were cut into small pieces and placed in the enzyme mixture. The tissues were chopped, repeatedly shaken vigorously, and placed in a shaker for digestion at 37 °C for 2 h. After adding 10 µl DNase, the samples were incubated at room temperature for 5 min, and the homogenates were then filtered through a 70-μm MACS Smart Strainer into a new 15-mL centrifuge tube and centrifuged to collect the precipitate. PBS was added to wash the pellets, and the cells were resuspended in medium to generate a single-cell suspension.

**Immunomagnetic bead sorting**

The suspensions generated above or cultured cell suspensions were added to PBS and centrifuged to collect the precipitate. Then, 80 µl buffer and 20 µl magnetic beads per 10^7^ cells were added, and the mixtures were incubate in the dark at 4 °C for 15 min. The cell pellets were collected by centrifugation and resuspended in 1 ml of buffer. The sorting column was installed according to the manufacturer's instructions (Miltenyi Biotec). Buffer was slowly added to wash the sorting column and the cell suspension was added. Then, the positive cells were bound to the sorting column, and the negative cells flowed out.

**Electron microscopy to identify autophagosomes**

The cells were digested with trypsin and centrifuged at 3000 r/min for 10 min. The supernatant was discarded, and 2.5% glutaraldehyde was slowly added to incubate for 24 h. Then, the cells were prepared according to the electron microscope section preparation procedure. The cells were observed under an electron microscope.

**Immunofluorescence**

A total of 1×10^5^ cells were seeded in a 24-well culture plate (slides were placed in advance), and the culture medium was discarded after 48 h of culture. Then, the cells were fixed in 4% paraformaldehyde and permeabilized with 0.5% Triton X-100. The cells were blocked with in 5% BSA. Primary antibodies were added and incubated overnight at 4 °C on a shaker. The fluorescent secondary antibody was diluted with blocking solution and incubated in the dark for 1 h at room temperature. Then, the nuclei were stained with DAPI for 5 min. Then, the slides were mounted with fluorescent mounting medium. An upright fluorescence microscope was used to observe and assess the staining index of positive cells.

**RNA extraction and quantitative real-time PCR**

Total RNA was extracted and purified using TRIzol (Invitrogen) according to the manufacturer's instructions. Then, 1 μg of total RNA was reverse transcribed using the PrimeScript RT kit (Vazyme). Real-time quantitative RT-PCR (qRT-PCR) was conducted using the QuantiTect SYBR Green PCR kit (Toyobo, Japan). The primer sequences are listed in Supplementary Table 3. β-Tubulin was used as the reference gene for normalization. The primers used for qRT-PCR are listed in Table S3.

**Coimmunoprecipitation and Western blotting**

Cells were lysed with WB&IP buffer (Beyotime) supplemented with protease inhibitors (Boster Biological Technology). The cell lysates were immunoprecipitated with the indicated primary antibodies overnight at 4 °C, followed by protein A/G precipitation for 2 h or direct incubated with magnetic beads conjugated with tagged antibodies (Biomake). The beads were washed 3 times with lysis buffer and eluted in SDS sample buffer. Eluted immune complexes were separated by SDS-PAGE followed by Western blotting.

Equal amounts of total protein were separated on 7.5/10/12.5% SDS-polyacrylamide gels and transferred to nitrocellulose membranes (Millipore). The membranes were incubated overnight with primary antibodies against the target proteins (Table S1). The membranes were washed 3 times with 1 x TBST buffer and incubated with secondary antibodies (Table S2) for 1 h at room temperature. The signals were visualized using Luminata crescendo western horseradish peroxidase substrate (Vazyme).

**Cell growth assay**

For cell proliferation, 2000 cells per well were seeded in 96-well plates. Cell viability was measured for five consecutive days using a Cell Counting Kit-8 (GLPBIO) according to the manufacturer's instructions. The absorbance at OD450 was used to generate cell growth curves. For clonogenic assays, 1000 cells were seeded in 6-well plates and maintained in DMEM/DF-12 medium supplemented with 10% FBS. After 14 days, the cells were washed with PBS and stained with 1% crystal violet.

**In vivo tumor xenograft models**

Cells were injected subcutaneously into NVSG mice (4-6 weeks, IL-2 knockout), obtained from Beijing Viewsolid Biotech Co. Ltd. The mice were anesthetized and sacrificed when they had lost approximately 20% of their body weight and when the tumor diameter reached a maximum of approximately 2 cm. The xenografts of each group were removed, weighed, and fixed in formalin. All the experimental protocols were approved by the Animal Ethics Committee of the Affiliated Hospital of Jining Medical University (No. 2022B041).

**RNA sequencing**

High-throughput RNA sequencing was performed with AQP5-overexpressing or knockdown AGS cells and negative control cells using standard procedures. Briefly, total RNA was extracted using TRIzol reagent, and Illumina high-throughput sequencing libraries were constructed and sequenced according to the manufacturer's instructions (Beijing Genomics institution).

**Immunochemistry**

Paraffin-embedded mouse and human tissues were deparaffinized in xylene, and antigens were recovered by boiling the samples for 20 min. The samples were incubated with primary antibodies overnight at 4 °C. The sections were stained with secondary antibodies for 30 min at room temperature and then stained with an EnVision-HRP kit (Dako).

**Construction of tissue microarrays**

Gastric cancer and adjacent normal tissues were used to construct tissue microarrays (Shanghai Biochip Co., Ltd., Shanghai, China). Tissue microarrays were stained for AQP5. Two pathologists independently scored the staining intensity of the arrays and the extent of protein expression across the sections.

**Mining the TCGA datasets**

RNAseq data in level 3 HTSeq-FPKM format from TCGA (https://portal.gdc.cancer. gov/) STAD (Stomach Cancer) project, RNAseq data in FPKM (Fragments Per Kilobase per Million) format were converted into TPM (transcripts per million reads) format, and log_2_ conversion was performed. Spearman's correlation coefficient was used to indicate the correlation between the expression of different genes.

**Flow cytometry analysis**

Tissues or adherent cells were processed into single-cell suspensions and stained using the antibodies listed in Table S1. The cells were analyzed by flow cytometry in a CytoFLEX Flow Cytometer. The data were analyzed using FlowJo software. The appropriate isotype controls were used. The control antibodies were used for gating.

**Cell migration**

Assays were performed in 24-well Transwell plates with 8-µm polyethylene terephthalate membrane filters (Falcon cell culture insert; Becton-Dickinson). Cells were seeded in serum-free DMEM at a density of 10^5^ cells per well in the upper chamber, and the lower chamber was filled with DMEM supplemented with 15% FBS. The cells were allowed to migrate for 18 hours. The cells that migrated to the lower surface of the filter were fixed with 4% paraformaldehyde, stained with 1% crystal violet, and counted in three random areas by microscopic imaging.

**GFP-LC3 reporter gene assay**

Different groups of cells were transfected with GFP-LC3 constructs according to the manufacturer's (Weizhen Biotechnology) protocol and subjected to cell culture. Then, 24 h later, live cells were imaged using a confocal microscope. The green spots were observed to assess autophagic flux.

**Statistical analysis**

All the data are presented as the mean ± standard deviation. Student’s t tests were used to analyze differences between groups. p < 0.05 was considered statistically significant.
